# Selection profiles in RNA viruses reflect the characteristics of viruses more than individual proteins

**DOI:** 10.1371/journal.ppat.1014457

**Published:** 2026-07-24

**Authors:** Laura Muñoz-Baena, Hugo G. Castelán-Sánchez, Sareh Bagherichimeh, Paula Magbor, Jorge Rojas-Vargas, Amjad Khan, Abayomi S. Olabode, Art F. Y. Poon

**Affiliations:** 1 Department of Microbiology & Immunology, Western University, London, Canada; 2 Department of Biology, University of Oxford, Oxford, United Kingdom; 3 Department of Pathology & Laboratory Medicine, Western University, London, Canada; 4 Department of Biology, Western University, London, Canada; 5 Department of Mathematics and Natural Sciences, Prince Mohammad Bin Fahd University, Al-Khobar, Saudi Arabia; 6 Department of Computer Science, Western University, London, Canada; The University of Arizona, UNITED STATES OF AMERICA

## Abstract

Proteins that are exposed on the surface of a virus are frequently subject to strong selection to escape from neutralizing antibodies. To investigate whether surface-exposed (SE) and non-exposed (NE) proteins encoded by RNA viruses exhibit different patterns of evolution under selection, we analyzed 244 protein-coding genes from 28 species of RNA viruses representing 15 taxonomic families. First, we show that gene-wide rates of non-synonymous (*dN*) and synonymous (*dS*) substitutions do not differentiate between SE and NE proteins. To incorporate variation in substitution rates among codon sites, we inferred the posterior distribution over a fixed grid of *dN* and *dS* rates for each alignment. This ‘evolutionary fingerprint’ provides a common framework for comparing the selection profiles of non-homologous genes. Next, we computed the Wasserstein distance for every pair of fingerprints, which is analogous to amount of work required to reshape one distribution to another. After compensating for differences in genetic variation among alignments, we found a small but significant difference between the fingerprints of SE and NE proteins (PERMANOVA, *P* = 0.03). However, we observed larger and more significant effects of whether the virus is enveloped (*P* < 10^-5^) and the interaction between these factors ($P=6.9\times 10^{-4}$). The latter effects were driven by high levels of purifying selection in capsid proteins of Picornaviruses. Furthermore, greater amounts of variation in fingerprints were explained by significant differences among virus families and modes of transmission (*P* < 10^-5^). These results imply the pattern of selection on a virus protein is shaped more by characteristics of the virus than the protein itself.

## Author summary

RNA viruses like influenza A virus and HIV-1 are well known for their ability to adapt rapidly to changing host environments. Much of this adaptation manifests in proteins on the surface of the virus that are responsible for recognizing and binding to host cells. In this study, we investigate whether these surface-exposed proteins can be distinguished from other virus proteins solely by how they evolve. We collected and processed over 42,000 genome sequences from 28 different RNA virus species. To compare fine-grained patterns of evolution in different proteins, even if they come from completely unrelated species, we use a technique called ‘evolutionary fingerprinting’. Our analysis reveals that surface-exposed proteins are only different within non-enveloped viruses, and that this is largely a characteristic of the family Picornaviridae, which includes poliovirus. In fact, the fingerprints of proteins are differentiated more by characteristics of the virus, such as taxonomic family or mode of transmission, than properties of the individual proteins like surface exposure.

## Introduction

Viruses have remarkably high rates of molecular evolution [1]. In particular, elevated mutation rates in RNA viruses, attributed to the low replication fidelity of the virus-encoded RNA-dependent RNA polymerase [2], can provide an abundance of raw material for a rapid response to selection. Selection in virus populations is predominantly shaped by their host environments. This environment may include the host cell receptor proteins targeted by a virus for binding [3], cellular components that are incorporated into the virus replication cycle [4], and both innate and adaptive immune responses [5]. Of these potential factors, the host adaptive immune response is arguably the most diverse and capable of changing at the compressed time scale of RNA virus evolution. Much of what we understand about selection in viruses comes from protein-coding genes [6]. In general, the proportion of RNA virus genomes that encodes proteins (*i.e.*, the coding density) is relatively high. The proteins encoded by a virus genome can be broadly categorized into structural and non-structural proteins, depending on whether the protein becomes part of the viral particle or remains within the cell. Some structural proteins comprise the outer capsid of non-enveloped viruses, or become embedded in the membrane of enveloped viruses. These surface-exposed proteins are the primary interface between the virus and the extracellular host environment. For instance, surface envelope glycoproteins such as HIV-1 gp120 [7] and influenza A virus hemagglutinin [8] are well-characterized targets of selection by neutralizing antibodies. Consequently, comparative studies of selection in viruses have tended to focus on the surface-exposed proteins, *e.g.*, [9,10]. On the other hand, significant positive selection has also been reported for genes encoding non-structural proteins or structural proteins that are not exposed on the surface of the virus particle [11].

Motivated by examples of diversifying selection targeting specific sites in surface-exposed proteins in RNA viruses, this study endeavours to determine whether different categories of virus proteins undergo distinguishable patterns of selection. Selection in protein-coding genes is typically identified by comparing the rates of amino acid-replacing (non-synonymous) and silent (synonymous) substitutions. When adjusted for the expected numbers of non-synonymous and synonymous substitutions, these rates become normalized quantities denoted respectively as *dN* and *dS* [12]. A relative excess of non-synonymous substitutions (*dN* > *dS*) provides evidence of positive selection, where selection promotes amino acid changes. Conversely, *dN* < *dS* implies negative (purifying) selection removing mutations away from the current amino acid sequence. Typically, we find substantial variation in *dN* and *dS* rates among sites in a protein-coding gene [13]. A plot of these site-specific rates along the length of the gene is frequently called its ‘selection profile’ [14,15], although there are other uses of this term in similar contexts [16]. In a constant and uniform selective environment, positive directional selection is a transient phenomenon that is resolved when a beneficial mutation becomes fixed in the population [17]. If we follow a single virus lineage through different host environments over time, the varying immune responses may lead to an excess of non-synonymous within-host polymorphisms [18]. Kistler and Bedford [19] recently demonstrated that lagging partial herd immunity can drive a sustained excess of non-synonymous substitutions (*dN* > *dS*) at a subset of sites in surface-exposed virus proteins. However, their analysis was limited to viruses with longitudinal samples of infections related by a single trunk lineage, *i.e.*, a ladder-like tree, because it relied on a comparison between substitutions (relative to a reference sequence in the past) and polymorphisms within a lineage (and its transient descendants) over time [20].

A more conventional approach to measuring selection in protein-coding genes is to fit a codon-substitution model across many divergent lineages that descend from a common ancestor [21]. In this context, positive selection driven by variation in selective environments is known as diversifying selection. This approach enables us to evaluate a broader selection of viruses. In this study, we examine whether different categories of virus proteins, including surface-exposed and non-exposed proteins, experience significantly different types of selection. Both synonymous and non-synonymous substitution rates can vary substantially among codon sites in a gene sequence [22]. Estimating these codon site-specific rates with reasonable accuracy requires a substantial amount of genetic variation in the sequence alignment. As a result, we focused specifically on a curated set of twenty-eight RNA virus species with a sufficient number of publicly available full-length genome sequences with an adequate level of evolutionary divergence. We first demonstrate that standard methods that reduce each alignment to a summary statistic, *e.g.*, the gene-wide dN/dS ratio, do not resolve significant differences between surface-exposed and non-exposed proteins. This implies that we require a more detailed method to compare site-specific patterns of selection between genes. The primary obstacle to this approach is that it is not obvious how one should compare site-level quantities between genes with no homology; for instance, HIV-1 envelope glycoprotein gp120 and enterovirus A71 helicase.

**Fig 1:**
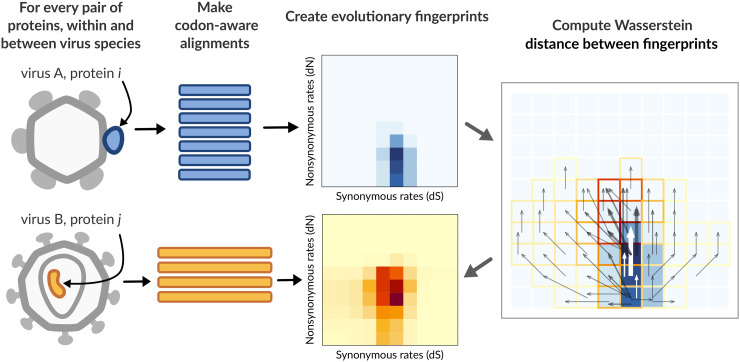
Comparing protein-coding genes via evolutionary fingerprints. We start with a multiple alignment of codon sequences for each protein-coding gene. The alignments vary in the number of codon sites (alignment length) and number of sequences. For each alignment, we model the variation in substitution rates among codon sites as a posterior probability distribution over a fixed grid of synonymous (*dS*) and non-synonymous (*dN*) codon substitution rates. This distribution, depicted here as a heatmap, is the gene’s ‘evolutionary fingerprint’. Lastly, we calculate the Wasserstein distance between every pair of fingerprints for genes *i* and *j* from viruses *A* and *B*, respectively; in some cases, the genes are from the same virus. This distance is the amount of work required to transform one distribution (shaded squares) to another (outlines), according to the optimal transport plan, which is represented by arrows of varying thickness for the amount of mass transported.

Pond *et al.* [23] described a statistical method to overcome this problem, which they dubbed ‘evolutionary fingerprinting’. The basic premise is that the rate variation among sites for a protein-coding gene alignment can be modeled as a latent discrete bivariate probability distribution over an *a priori* fixed grid of *dN* and *dS* rates. A flat prior distribution over this grid is updated by the phylogenetic likelihood of the codon alignment. The resulting posterior distribution over the grid is the evolutionary fingerprint of that alignment. Hence, the evolutionary fingerprint provides a common framework for comparing unrelated genes (Fig 1). We identify significant challenges that arise in applying fingerprinting to alignments from a broad diversity of rapidly-evolving species and genes, and develop methods to address these issues that have not been described in previous work [e.g., 24–26]. Using this approach, we determine that there is not evidence that surface-exposed proteins overall undergo selection any differently than other virus proteins. However, we observe that surface-exposed proteins associated with non-enveloped viruses have fingerprints that are significantly different from their non-exposed counterparts, and that this trend is largely driven by purifying selection on exposed capsid proteins in picornaviruses. Finally, we test whether associations exist between evolutionary fingerprints and virus-level characteristics, specifically their taxonomic relationships and modes of transmission.

## Methods

### Data collection

We manually queried the NCBI Genbank database to select candidate RNA virus species on the basis of two criteria: the availability of at least 100 complete or near-complete genome sequences, and the existence of an annotated reference genome, *i.e.*, RefSeq record [27]. In addition, we excluded sequences associated with patents, laboratory clones or modified nucleic acids. For each candidate species, we filtered the search results by taxonomic identifier and minimum sequence length based on the expected genome length, and then downloaded a list of accession numbers. If the virus had a segmented genome, *e.g.*, influenza A virus, then we manually composed queries including gene identifiers and exported separate lists of accession numbers. We anticipated that the genetic diversity captured in human immunodeficiency virus type 1 (HIV-1) sequences would be disproportionately greater than other viruses due to extensive sequencing of HIV-1 infections at a global scale. Consequently, we restricted our analysis to infections classified as sub-subtype A1, which is predominantly found in east Africa and central Asia [28]. We queried the Los Alamos National Laboratory HIV Sequence Database (https://www.hiv.lanl.gov) for sub-subtype A1, limiting the search results to one record per individual, and then extracted the Genbank accession numbers for subsequent steps. For influenza A virus (IAV), sequences from human hosts (predominantly subtypes H3N2 and H1N1) tend to induce highly ladder-like trees due to short infectious periods and transient cross-immunity in the host population [29]. This scenario is not consistent with diversifying selection as measured by comparative dN/dS methods [17]. Consequently, we restricted our search to subtype H9N2 infections isolated from avian hosts, where multiple co-circulating lineages with low pathogenicity have become endemic in commercial poultry [30].

For virus genomes in which genes were annotated separately as ‘mat_peptide’ features, we used the BioPython [31] interface to the NCBI Entrez API to retrieve all protein-coding sequences (CDSs) associated with the records corresponding to a given set of accession numbers. This yielded a FASTA file containing multiple sequence records for every genome, with sequence labeled with protein name, genome strand and genome coordinates. We used the same script to extract sample metadata from the SeqRecord object, *e.g.*, sample collection date. Next, we used MAFFT (version 7.49) [32] to align the amino acid translation of each sequence against the set of mature peptides from the reference genome. We assigned each sequence to the reference peptide that attained the highest alignment score, given a match score of +1, a mismatch penalty of −1 and a linear gap penalty of −3. For virus genomes in which proteins are derived from a polyprotein encoded by a single open reading frame, *e.g.*, hepatitis C virus, we used a similar pairwise method to align the polyprotein sequence pairwise to every mature peptide feature in the reference genome, and extracted the corresponding nucleotide substring to a separate FASTA file for each feature.

We classified each protein-coding gene as ‘surface-exposed’ if any portion of the mature peptide was documented (*e.g.*, ViralZone [33]) to be exposed on the outer surface of the virus particle released into the extracellular environment. Proteins from plant viruses were not labeled as ‘surface-exposed’ even if they are exposed on the surface of the virus particle, because host plants do not have an adaptive immune system. In addition, we annotated genes that encode enzymatic proteins with polymerase or protease activity, or structural proteins. We annotated virus species by whether they are enveloped or non-enveloped.

### Phylogenetic analysis

For each FASTA file produced in the preceding step, we used a Python script to generate a multiple alignment of the amino acid translations of the sequences using MAFFT, and then applied the gaps in this alignment to the original nucleotide sequences to obtain a codon alignment preserving the reading frame. We used AliView [34] to visually inspect the resulting alignment, and manually removed problematic sequences, *e.g.*, CDS records labeled with the wrong protein. Positions where a majority of sequences contained a gap were removed from the alignment in a codon-aware manner. Incomplete sequences that were shorter than half of the alignment length were excluded. We used FastTree (version 2.1.11, compiled for double precision) [35] to reconstruct a preliminary maximum likelihood tree from the resulting alignment. The tree was visually inspected for excessively long branches, *i.e.*, exceeding two standard deviations above the mean. Any outlier sequences identified at this step were removed and the tree was rebuilt from the updated alignment.

### Selection analysis

Many virus genomes contain overlapping genes in different reading frames as a potential mechanism for increasing the information content of a compact genome [36]. Overlaps of protein-coding genes cause problems for measuring selection because a substitution that is synonymous in one reading frame may be non-synonymous in another [37]. To reduce the influence of overlapping genes, we manually removed codon sites affected by overlaps in each alignment. Genes encoding multiple products due to alternate initiation or termination sites were not modified (*e.g.*, VP7 in rotavirus A), unless one of those alternate products involved splicing a frame-shifted portion of the gene (*e.g.*, influenza A virus PB2-S1). Any intervals involved in an overlap with different reading frames was removed from all affected gene alignments. Detailed reference coordinates of the final gene alignments are provided in Supporting Information (S1 Table).

For each gene alignment, we reconstructed a maximum likelihood phylogeny using FastTree and then fit a Muse-Gaut codon substitution model crossed with a general time-reversible model of nucleotide substitutions in HyPhy to measure the gene-wide *dN*/*dS* ratio as a global parameter. We used the single likelihood ancestor counting (SLAC) method to estimate individual codon site-specific *dN* and *dS* values. Because gene-wide *dN*/*dS* ratios were right-skewed and strictly positive, we used a gamma regression model with a log-link function to evaluate the effects of whether the *i*-th virus in family *j* is enveloped ($e_{ij}$) and the *k*-th protein of the virus is surface-exposed ($x_{ijk}$) on *dN*/*dS*. To account for variation in dN/dS=ω among virus species, we fit a mixed-effects log-link model using the R package *lme4* [38]:


$$\begin{array}{c} \log\omega_{ijk}=\alpha_{j}+\alpha_{ij}+\beta_{1}e_{ij}+\beta_{2}x_{ijk}+\beta_{3}e_{ij}x_{ijk}+\epsilon \end{array}$$

where $\alpha_{j}$ and $\alpha_{ij}$ are family-specific and species within family random intercept terms, $\beta_{•}$ are fixed effects, and ϵ represents residual (error) variance. We generated 95% confidence intervals (CI) for model parameters by bootstrap resampling, and reported these intervals alongside the *P*-values where applicable. A term was considered to have a significant fixed or random effect if the 95% CI did not include zero. In addition, we used a binomial regression model with a logit link function to analyze the proportion $p_{ik}$ of codon sites in gene *k* for virus *i* under significant (α=0.1) diversifying (*dN* > *dS*) or purifying (*dN* < *dS*) selection:


$$\begin{array}{c} \log\left(\frac{p_{ik}}{1-p_{ik}}\right)=\alpha_{i}+\beta_{1}e_{i}+\beta_{2}x_{ik}+\beta_{3}e_{i}x_{ik}+\epsilon . \end{array}$$

Note that we simplified this model to virus-specific random effects ($\alpha_{i}$) only, as the full model with nesting with families did not confer a significant improvement in fit.

### Evolutionary fingerprinting

We used the Fast Unconstrained Bayesian Approximation [FUBAR; 39] method in *HyPhy* [version 2.5.60; 40] to estimate the site-specific synonymous (*dS*) and non-synonymous (*dN*) substitution rate parameters for each codon alignment. This method approximates a latent bivariate distribution of continuous *dN* and *dS* values with a discrete posterior probability distribution *f* over a fixed 20×20 grid of values, which Pond *et al.* [23] dubbed the ‘evolutionary fingerprint’ for the alignment. Following Murrell *et al.* [25], we replaced the default grid values in FUBAR with a smoother distribution of rates generated by the formula $(50\times k^{5})/19^{5}$ for k={0,1,…,19}. In addition, we increased the length of the chain sample from the default $2\times 10^{6}$ to 10^7^ steps to improve sample convergence. The spread of a fingerprint $f_{ij}$ was quantified by the determinant of the covariance matrix:


$$\begin{array}{c} \det(\begin{array}{cc} \sum_{i}i^{2}f_{i}-\mu_{i}^{2} & \sum_{ij}ijf_{ij}-\mu_{i}\mu_{j}\\ \sum_{ij}ijf_{ij}-\mu_{i}\mu_{j} & \sum_{j}j^{2}f_{j}-\mu_{j}^{2} \end{array}) \end{array}$$

where i,j∈{1,…,20} are integer indices for *dS* and *dN* grid values, $f_{i}=\sum_{j}f_{ij}$ is the marginal probability for *i*, and $\mu_{i}=\sum_{i}if_{i}$ is the mean for *i*. This quantity is also known as the generalized variance.

We used the Wasserstein distance — also known as the Monge-Kontorovich distance [23] and more descriptively as the earth mover’s distance — as implemented in the R package *transport* [41] to compare fingerprints obtained from two different alignments. This distance is analogous to the minimum amount of work required to reshape one distribution to another, accounting for the distance between points on the grid (Fig 1). We used the Euclidean norm for calculating the distance between points on the grid. Each grid point was represented by the log-transformed rate offset by a small constant (ϵ=0.05) to accommodate the zero rate class, *i.e.*, $\tilde{r}=\log(r+\epsilon )$ where *r* represents either *dN* or *dS*. This distance is represented by the following formula:


$$\begin{array}{c} W(f,g)={\left(\inf_{\pi \in \Pi (f,g)}\sum_{r_{f},s_{f}}\sum_{r_{g},s_{g}}\left[({\tilde{r}}_{f}-{\tilde{r}}_{g}{)}^{2}+({\tilde{s}}_{f}-{\tilde{s}}_{g}{)}^{2}\right]\pi (f,g)\right)}^{1/2} \end{array}$$

where *f* and *g* are two discrete probability distributions (evolutionary fingerprints), π is a plan for transporting probability mass between points to reshape *f* to *g*, Π(f,g) is the set of all possible plans to transform *f* into *g*, $\tilde{r}$ and $\tilde{s}$ are the lo*g*-offset values associated with the grid points for *dS* and *dN* rates, and the infimum (inf) identifies the most efficient plan out of all valid options. Similar results were obtained using the inte*g*er indices {i,j}={1,2,…,20} associated with grid points instead of the log-offset *dS* and *dN* rates.

The distribution of evolutionary fingerprints induced by a Wasserstein distance matrix was visualized by multidimensional scaling (MDS) in two dimensions using the R function *cmdscale*. To test whether factors such as surface exposure induced a significant partition of variance in the corrected Wasserstein distances among proteins, we used the *adonis2* implementation of permutational analysis of variance (PERMANOVA) in the R package *vegan* [42]. We ran each test with at least 10^4^ permutations.

### Adjusting for genetic variation

Our preliminary analyses indicated that measuring evolutionary fingerprints was sensitive to the amount of genetic variation captured by the alignment. We used the tree length, *i.e.*, sum of branch lengths, to quantify this genetic variation. In addition, we observed that fingerprints were affected by alignment length. Intuitively, the number of codon sites in the alignment (Supporting Information, S1A Fig) roughly corresponds to the sample size for capturing genetic variation. For the purpose of visualization, we employed two different methods to separate the effect of sampled genetic variation from the association between the evolutionary fingerprint and intrinsic properties of the virus protein. First, we fit linear regressions of the two sets of MDS coordinates onto the tree lengths and alignment lengths. Log-transformations of these two covariates yielded substantially improved goodness-of-fit for the linear models. Next, we generated a new distance matrix from the residuals of the two regression models. Finally, we produced a new MDS from this distance matrix with the main effects of tree length and alignment length removed. This approach is similar to partial distance-based redundancy analysis used frequently in community ecology [43].

Second, we used downsampling to reduce variation in both tree length and alignment length among viruses and proteins. To determine the lowest acceptable tree length, we used INDELible version 1.03 [44] to simulate codon sequence alignments with known site-specific *dN* and *dS* values. We seeded the simulation with a random tree relating 100 tips that was generated under a constant size coalescent model. This input tree was rescaled to different lengths and the resulting alignments were analyzed using the FUBAR method. We calculated the root mean square error (RMSE) between the known and estimated *dN*/*dS* ratios. Based on the initial distribution of tree lengths for actual data (Supporting Information, S1B Fig) and the association between RMSE and tree lengths for simulated data (S2 Fig), we chose a target range of 0.5 to 2.0 expected nucleotide substitutions per site (ESS; S1C Fig).

To normalize tree lengths across alignments, we progressively removed the shortest terminal branches from the starting tree until the length fell below a cutoff of 2.0 ESS. This pruning approach maximized the amount of genetic variation for a given subset of sequences. In many cases, it was not possible to prune the tree down to the cutoff length because of long internal branches in the tree. For these alignments, we arbitrarily selected a starting terminal branch and proceeded toward the root until we reached an internal node that rooted a monophyletic group with a total length below the cutoff. If the starting tree length was already between 0.5 and 2.0 ESS, then the alignment was passed to next steps without modification. Alignments with a starting tree length below 0.5 ESS were discarded from further analysis. To address the effect of alignment length, we generated 10 replicate subsets by sampling *L* = 50 codon sites from each alignment at random without replacement. We processed the sampled alignments using the same workflow, and then calculated the centroid for each set of replicates by averaging their coordinates in the multidimensional scaling projection. This analysis was also repeated with a higher threshold of *L* = 100 codon sites.

We used permutational multivariate analysis of variance (PERMANOVA), as implemented in the *adonis2* function in R package *vegan* [45], to test whether statistically significant amounts of variation in Wasserstein distances among evolutionary fingerprints can be attributed to characteristics of the corresponding proteins, such as surface exposure. This method was applied directly to the Wasserstein distance matrix, instead of the residualized distances used for visualization, to preserve the relationships among fingerprints in the original high dimensional space. We used sequential addition of terms to remove the confounding effects of (log-transformed) alignment and tree lengths before evaluating the significance of subsequent terms. We ran a minimum of 9,999 permutations for each analysis.

All sequence data at different stages of processing have been deposited into a public online repository at https://doi.org/10.5281/zenodo.16320684 under a permissive license (Creative Commons Attribution 4.0 International). Python and R scripts implemented for this study have been published under the MIT license at https://github.com/PoonLab/surfaces.

## Results

### Gene-wide selection

**Fig 2:**
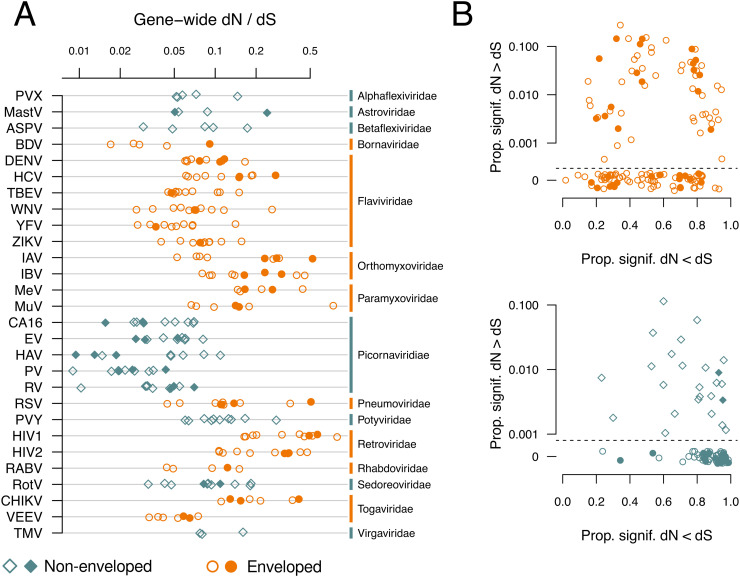
Summary of dN and dS estimates for protein-coding gene alignments. **(A)** Gene-wide dN/dS estimates per protein and virus. Each point represents a gene alignment, grouped by virus (left labels) and by family (right labels). A point is filled if the protein is located on the surface of the virus particle and potentially exposed to an adaptive immune response. Colour and shape are used to distinguish proteins from enveloped (circle, orange) and non-enveloped (diamond, blue) viruses. **(B)** Scatterplots of the proportions of codon sites under statistically significant (α=0.1) purifying (*dN* < *dS*, *x*-axis) and diversifying (*dN* > *dS*, *y*-axis) selection for enveloped (top) and non-enveloped (bottom) viruses. Each point represents a gene alignment, using the same shape and colour scheme as **(A)**. The *y*-axis was log-transformed to accommodate skewed distributions in the proportions of *dN* > *dS* sites, with an axis break for zero counts, including random noise to reduce overlap.

We obtained protein-coding sequences from over 42,000 genomes for 28 different RNA virus species representing 15 different families (Table 1). These data largely comprised human viruses that pose a significant threat to human health, but we did not limit our analysis to viruses from human hosts — we also obtained data for several agriculturally-significant plant RNA viruses such as potato virus Y [46]. Fig 2A displays the mean estimates of dN/dS for each alignment of protein-coding gene sequences. As expected, all mean dN/dS values were well below 1, indicating that a majority of codon sites in any given gene were under purifying selection. Mean dN/dS values were generally lower in proteins associated with non-enveloped viruses (average 0.067 versus 0.158 in enveloped viruses; Welch two-sample *t*-test, $P=3.87\times 10^{-11}$), which were disproportionately represented by members of the family Picornaviridae.

**Table 1:** Summary of viruses analyzed in this study. *Abbrv.* = conventional abbreviation used for figures. *Env?* = is enveloped virus? *Genomes* = initial number of genome records obtained prior to filtering. *Proteins* = number of protein-coding genes analyzed for selection; note this number excludes genes of insufficient length or with extensive overlaps with other genes.

Family	Virus	Abbrv.	Env?	Genomes	Proteins
Alphaflexiviridae	potato virus X	PVX	No	409	5
Astroviridae	mamastrovirus	MastV	No	125	4
Betaflexiviridae	apple stem pitting virus	ASPV	No	166	5
Bornaviridae	Borna disease virus	BDV	Yes	118	5
Flaviviridae	dengue virus 2	DENV	Yes	1,630	10
	hepatitis C virus (genotype 1a)	HCV	Yes	3,911	10
	tick-borne encephalitis virus	TBEV	Yes	440	11
	West Nile virus	WNV	Yes	2,313	11
	Yellow fever virus	YFV	Yes	1,304	10
	Zika virus	ZIKV	Yes	664	10
Orthomyxoviridae	influenza A virus (H9N2)	IAV	Yes	2,220*	10
	influenza B virus	IBV	Yes	13,224*	10
Paramyxoviridae	measles virus	MeV	Yes	825	6
	mumps virus	MuV	Yes	721	7
Picornaviridae	coxsackievirus A16	CA16	No	514	10
	enterovirus A71	EV	No	1261	10
	hepatitis A virus	HAV	No	362	9
	polio virus	PV	No	270	10
	rhinovirus A	RV	No	1347	10
Pneumoviridae	respiratory syncytial virus	RSV	Yes	1,021	11
Potyviridae	potato virus Y	PVY	No	911	10
Retroviridae	human immunodeficiency virus type 1 (subtype A)	HIV1	Yes	256	12
	human immunodeficiency virus type 2	HIV2	Yes	121	12
Rhabdoviridae	rabies virus	RABV	Yes	2,229	5
Sedoreoviridae	rotavirus A	RotV	No	4,635*	11
Togaviridae	Chikungunya virus	CHIKV	Yes	978	7
	Venezuelan equine encephalitis virus	VEEV	Yes	220	9
Virgaviridae	tobacco mosaic virus	TMV	No	108	4

*For viruses with segmented genomes, we reported the maximum number of sequences for any segment.

Next, we evaluated support for the hypothesis that surface-exposed virus proteins have relatively more sites under diversifying selection, which would drive up the mean dN/dS ratio. We note that surface-exposed proteins for viruses infecting plants were excluded from this category because plants do not have an adaptive immune response that would drive diversifying selection among hosts. The effect of relaxing this assumption is evaluated in a later section. Ignoring variation in mean dN/dS values among viruses, we found no significant effect of surface exposure in a gamma regression model (*P* = 0.095, 95% CI = −0.036,0.59), where we used a log-link function to account for the skew of this ratio outcome. If we switched to a mixed-effects log-link model to address the inherent structure in these data (*i.e.*, repeated measures from each virus species nested within families), we observed significant variation among viruses (standard deviation 95% CI = 0.40, 0.83) and families (95% CI = $9.1\times 10^{-6}$, 0.95). However, there was still no significant effect of surface exposure (*P* = 0.087, 95% CI = −0.068,0.36). Adding an interaction between surface exposure and being an enveloped virus conferred an improved model fit (ΔAIC=3.7), but the main effect of surface exposure (95% CI = −0.54,0.16) becomes absorbed into the interaction term (95% CI = 0.033, 0.88). This significant interaction implies that the selective regimen experienced by surface-exposed proteins depends on whether the virus is enveloped.

Measuring selection at the level of whole genes may obscure more significant effects on a small number of codon sites. Typically only a fraction of the amino acids in a protein are actually exposed on the surface of the virus, for instance [7]. Fig 2B summarizes the proportions of codon sites under significant site-specific diversifying (*dN* > *dS*) or purifying (*dN* < *dS*) selection for each gene. We fit a mixed-effects model to the number of sites with significant diversifying selection (*dN* > *dS*) as a binomial outcome accounting for the total number of sites, with virus species as a random effect, and with surface-exposure and enveloped virus as main and interaction fixed effects. Models dropping any of these independent variables were rejected (ΔAIC≥14.1). As before, there was significant variation among virus species (standard deviation 95% CI = 1.34, 2.67). However, the model did not support nesting species with family (ΔAIC=−0.9). There was a significant interaction effect ($P=6.2\times 10^{-4}$), where a protein being both surface-exposed and in an enveloped virus increased the log-odds of positively-selected sites by 1.78 (95% CI = 0.83, 3.0). On its own, exposure had a significant negative effect (*P* = 0.026, 95% CI = −2.36, −0.21), which is in the opposite direction that we would expect, *i.e.*, surface-exposure is hypothesized to promote diversifying selection. Being enveloped had no significant effect on the log-odds (*P* = 0.84, 95% CI = −1.45, 1.72).

We repeated the same mixed-effects model analysis on the number of sites with significant purifying selection (*dN* < *dS*). Again, there was significant variation among virus species (standard deviation 95% CI = 0.34, 0.58). There was no significant effect of surface exposure on this outcome (*P* = 0.84, 95% CI = −0.04, 0.05) and no significant interaction effect between exposure and enveloped (*P* = 0.12, 95% CI = −0.101, 0.012). However, being associated with an enveloped virus significantly decreased the log-odds of negatively-selected sites (*P* = 0.015, 95% CI −0.75, −0.07). Overall, these results suggest that the relationship between surface-exposure and selection regime experienced by a protein is dependent on the type of virus encoding that protein (*i.e.*, enveloped versus non-enveloped), although this may be confounded by taxonomic grouping.

### Evolutionary fingerprints

Ideally, we want to compare sets of site-specific estimates of *dN* and *dS* between two genes, rather than comparing a single number, such as the gene-wide average *dN*/*dS* (Fig 2A) or proportion of sites with significant diversifying or purifying selection (Fig 2B). These gene alignments will usually be non-homologous and can differ substantially in length. Consequently, it is not feasible to directly match rate estimates at codon sites from different genes in a meaningful way. Pond *et al.* [23] proposed a method to characterize a gene by assuming that the site-specific rates are drawn from a latent bivariate probability distribution of *dN* and *dS*, dubbed the ‘evolutionary fingerprint’. By constraining this distribution over a fixed grid of *dN* and *dS* values, this fingerprint provides a common framework in which one can compare completely unrelated genes. We employed this method to generate the fingerprints for the protein-coding gene alignments in our study. To analyze the evolutionary fingerprints in a quantitative framework, we calculated the Wasserstein distance between every pair of fingerprints (Fig 1). This distance roughly corresponds to the amount of ‘work’ required to transform one distribution into another. An important advantage of the Wasserstein distance over alternatives such as the angular distance (related to the Pearson correlation coefficient [25]) is that the latter penalizes differences in probability masses irrespective of where the masses are located. For example, consider three fingerprints (*A*, *B* and *C*) that respectively have 90% probability at grid points (1, 9), (1, 10) and (20, 20), and the remaining probability is otherwise uniformly distributed among points. The angular distance between *A* and *B* will be exactly the same as *A* and *C*, but the Wasserstein distance (*W*) recognizes that reshaping *B* to *A* requires much less work than *C* to *A*, resulting in $W_{AB}<W_{AC}$.

Our preliminary analyses of the Wasserstein distances between evolutionary fingerprints revealed that the shape of a fingerprint is sensitive to the number of codon sites (alignment length; Supporting Information, S1A Fig). For instance, there was a significant positive correlation (Pearson’s *r* = 0.81, *P* < 10^-6^) between Wasserstein distance between fingerprints and the difference in log-transformed alignment lengths (S3 Fig). Fingerprints are also affected by the extent of evolutionary divergence among sequences, which we quantified by tree length (S1B Fig). Specifically, larger trees corresponding to more diverse sequence alignments tend to yield more granular fingerprints for the same protein (S4 Fig). This confounding effect of genetic variation is more visible when we use multidimensional scaling (MDS) to project the Wasserstein distance matrix into two dimensions (S5 Fig). Alignment lengths were strongly correlated with both the first (Spearman’s ρ=0.91, *P* < 10^-12^) and second MDS coordinates (ρ=−0.26, $P=6.3\times 10^{-5}$). In contrast, tree lengths were significantly correlated with the second coordinate (ρ=−0.16, *P* = 0.01), but not the first (ρ=0.06, *P* = 0.32).

For the purpose of visualizing associations between evolutionary fingerprints and biological characteristics, we used two strategies to control for the effects of genetic variation on the fingerprints. First, we regressed out the effects of alignment length and tree lengths from the MDS coordinates, and generated a new distance matrix from the residuals. Second, we downsampled each alignment by removing sequences associated with the longest terminal branches in the corresponding phylogeny, until the tree length approached a target of 2.0 expected nucleotide substitutions per site (Supporting Information, S1C Fig); alignments with an initial tree length below 0.5 were discarded. Following this downsampling step, we sampled *L* = 50 or *L* = 100 codon sites at random without replacement from each alignment. Reconstructing trees and evolutionary fingerprints from these samples yielded two sets of 10 replicate distance matrices. Replicate samples of codons from the same downsampled alignment were tightly clustered with respect to Wasserstein distances (S6 Fig), indicating that the inherent differences among virus proteins were retained despite downsampling. The results of both strategies to correct for genetic variation are summarized by MDS plots in S7 Fig. Although the two methods are quite different, the residualized and downsampled distance matrices were significantly correlated (Mantel test *r* = 0.693, $P\leq \;10^{-5}$ for *L* = 100; *r* = 0.645, $P\leq \;10^{-5}$ for *L* = 50). Moreover, the subsequent analytical results were qualitatively the same. For brevity, we will report the results from the first method (residualized distances) in the main text, with results from downsampled distances provided as Supporting Information (S8 and S9 Figs).

**Fig 3:**
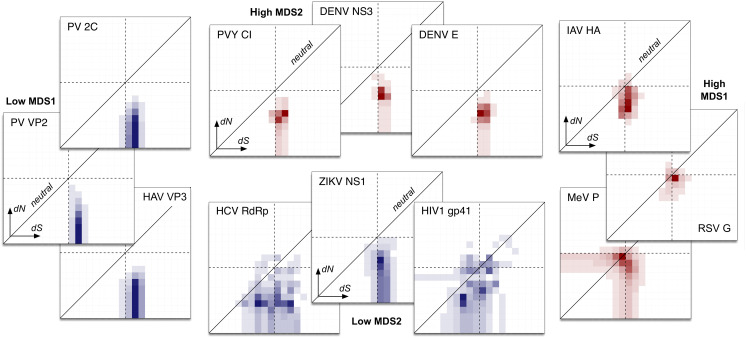
Representative samples of evolutionary fingerprints as heatmaps. Each heatmap represents the evolutionary fingerprint of a virus protein as a probability distribution on a fixed grid of *dS* (*x*-axis) and *dN* (*y*-axis) values. A diagonal line marks rates associated with neutral evolution (*dN* = *dS*), and dashed lines are drawn at the midpoints to facilitate comparison between heatmaps. These fingerprints are arranged to approximate their locations in the MDS projection of residualized Wasserstein distances (Fig 4). The leftmost (PV 2C, PV VP2, HAV VP3) and rightmost (IAV HA, RSV G, MeV P) groups have low and high positions on the first coordinate (MDS1), while being roughly centered along the second coordinate (MDS2). Conversely, the lower (HCV RdRp, ZIKV NS1, HIV1 gp41) and upper (PVY CI, DENV NS3, DENV E) groups have low and high positions on MDS2 while being centered on MDS1. Colours are used as a visual cue for fingerprints that are associated with high (red) and low (blue) MDS coordinate values, respectively.

Representative samples of fingerprints at the lowest and highest limits of each coordinate in the MDS projection of the residualized Wasserstein distance matrix are displayed in Fig 3. The first coordinate (MDS1) was positively correlated with mean gene-wide *dN*/*dS* ratios (Spearman’s ρ=0.89, *P* < 10^-6^), while the second coordinate (MDS2) was not correlated (ρ=0.004, *P* = 0.95). The correlation with MDS1 was confirmed by visual assessment of evolutionary fingerprints at low and high positions along this coordinate (Fig 3). Fingerprints from the ‘left’ side of the MDS projection tended to have posterior probabilities concentrated at grid points with lower *dN* relative to *dS*. On the opposite ‘right’ side, the peak posterior probabilities approached the *dN* = *dS* line of neutral evolution at the center of the grid. Fingerprints sampled from the center of MDS1 were intermediate of these two extremes; these fingerprints were chosen from the extremes of MDS2. Based on our visual assessment of evolutionary fingerprints, MDS2 is negatively associated with variation in the posterior distribution over *dS* and *dN* rates, with ‘flatter’ fingerprints associated with lower positions on this coordinate. This was confirmed by a correlation test of the generalized variance of fingerprints against this MDS coordinate (ρ=−0.23, $P=2.96\times 10^{-4}$).

### Differences among groups

**Fig 4:**
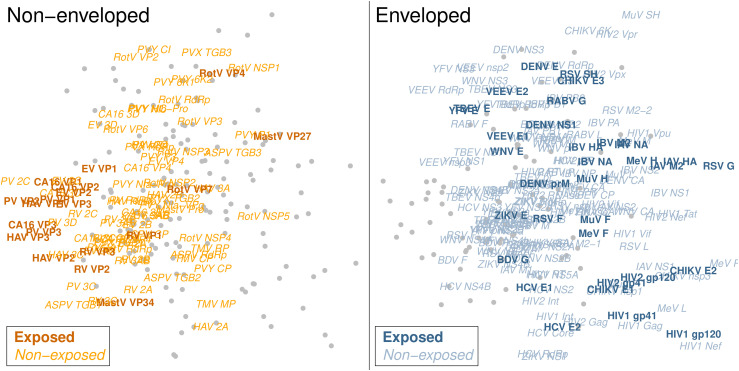
Multidimensional scaling plots of residualized Wasserstein distances. Each point represents the evolutionary fingerprint of a gene alignment. The x− and *y*-axes capture 79.2% and 20.8% of the variance, respectively. Points were labeled with the respective virus and protein (abbreviations defined in Table 1 and Supporting Information, S1 Table) for enveloped (left) and non-enveloped (right) viruses. We varied label style and colours to differentiate surface-exposed (bold, darker) and non-exposed (italicized, lighter) proteins.

Fig 4 illustrates the MDS projection for the residualized distance matrix, highlighting proteins that are surface-exposed or non-exposed, respectively. A key issue with the residualization method is that the Wasserstein distances have been projected into a two-dimensional MDS space, discarding about 34% of the variation before regressing out the confounding factors. As a result, the residuals are extracted from a 2D plane and the subsequent MDS is simply a rotation, which is why the two components seem to explain 100% of the variation. A simpler approach to isolate associations between fingerprints and biological features from the confounding effects of genetic variation is to perform permutational analysis of variance (PERMANOVA) tests directly on the original Wasserstein distance matrix. With the sequential addition of terms, significant effects of alignment length (*R*^2^ = 0.60, *P* < 10^-5^) and tree length (*R*^2^ = 0.025, *P* < 10^-5^) were first removed as technical confounders. We obtained a relatively small but significant effect of exposed (*R*^2^ = 0.005, *P* = 0.030), a stronger effect of enveloped (*R*^2^ = 0.032, *P* < 10^-5^), and a highly significant interaction between these factors (*R*^2^ = 0.012, $P=6.9\times 10^{-4}$). If we substituted polymerase activity for surface exposure, we obtained a small but significant main effect (*R*^2^ = 0.0089, *P* = 0.004), and no significant interaction with being enveloped (*R*^2^ = 0.0014, *P* = 0.34). Similar results were obtained for the distance matrices from samples of *L* = 50 and 100 codons from the downsampled alignments (Supporting Information, S8 and S9 Figs), with the exception that the main effect of surface exposure was no longer significant (*P* > 0.2).

The preceding PERMANOVA analyses indicate that surface exposure, on its own, has only a small effect on evolutionary fingerprints relative to being associated with an enveloped or non-enveloped virus. Put another way, it shows that exposure must be interpreted in the context of whether the virus is enveloped, which is consistent with our results from conventional *dN*/*dS* analysis. Non-enveloped viruses in our data set were disproportionately represented by members of the family Picornaviridae (Table 1). This raises the possibility that the preceding results were primarily driven by differences among virus families, rather than differences between proteins. Indeed, we found significant clustering of evolutionary fingerprints by virus family (*R*^2^ = 0.11, *P* < 10^-5^). This is illustrated for the six families with multiple species in Supporting Information (S10 Fig). Furthermore, the proportion of variance explained by virus family was an order of magnitude greater than the combined effects of exposed and its interaction with enveloped (total *R*^2^ = 0.011). We note that incorporating this factor into the PERMANOVA analysis caused the main effect of enveloped to be automatically dropped due to its collinearity with family.

Furthermore, we ran family-wise PERMANOVA tests to assess whether proteins within each family were significantly clustered relative to all other proteins (‘one versus rest’). After adjusting for multiple comparisons, we found that Orthomyxoviridae, Paramyxoviridae, Picornaviridae and Retroviridae were significantly clustered (adjusted P≤0.002, Table 2). Our power to detect clustering at this level was affected by sample size; for example, Virgaviridae was represented by three protein-coding genes, limiting statistical power. On the other hand, the virus family with the largest sample size (Flaviviridae) was not significantly clustered (adjusted *P* = 0.17).

**Table 2:** Summary of family-wise PERMANOVA tests on the Wasserstein distance matrix. The confounding effects of alignment and tree lengths were sequentially isolated as log-transformed terms in the PERMANOVA model. ‘One versus rest’ tested the grouping of all proteins in each virus family against all other proteins. ‘Within (exposed)’ tested the grouping of surface-exposed and non-exposed proteins in each family. Some ‘within’ tests could not be run for families with an insufficient number of proteins.

		One versus rest			Within (exposed)			
Family	*N*	*R*^2^ (%)	*P*	*P*_adj_	*n*_ex_	*R*^2^ (%)	*P*	*P*_adj_
Alphaflexiviridae	5	0.13	0.41	0.47	0			
Astroviridae	4	0.11	0.50	0.53	2			
Betaflexiviridae	5	0.27	0.17	0.31	0			
Bornaviridae	5	0.28	0.15	0.31	1	5.3	0.54	0.57
Flaviviridae	62	0.42	0.067	0.17	9	0.64	0.20	0.51
Orthomyxoviridae	19	1.40	$5\times 10^{-4}$	**0.002**	6	2.71	0.12	0.51
Paramyxoviridae	25	1.52	$2\times 10^{-4}$	**0.001**	4	1.93	0.56	0.57
Picornaviridae	52	5.16	10^-4^	$7.5\times 10^{-4}$	15	3.08	0.0079	0.079
Pneumoviridae	17	0.23	0.20	0.31	3	1.76	0.56	0.57
Potyviridae	10	0.22	0.24	0.31	0			
Retroviridae	22	2.33	10^-4^	$7.5\times 10^{-4}$	4	2.84	0.17	0.51
Rhabdoviridae	5	0.10	0.53	0.53	1	4.81	0.57	0.57
Sedoreoviridae	11	0.64	0.024	0.071	2	2.73	0.33	0.56
Togaviridae	7	0.20	0.25	0.31	5	2.03	0.29	0.56
Virgaviridae	3	0.23	0.21	0.31	0			

*N* = the total number of proteins for all viruses in the family, *n*_ex_ = the number of surface-exposed proteins, *R*^2^ = proportion of variance explained by partition, *P* = unadjusted *P*-value, *P*_adj_ = Benjamini-Hochberg adjusted *P*-value.

Next, we used permutation tests to determine whether surface-exposed proteins were separable from non-exposed proteins within the same family. Overall, fingerprints remained significantly clustered by family (PERMANOVA, *R*^2^ = 0.11, *P* < 10^-4^) and surface exposure (*R*^2^ = 0.005, *P* = 0.012). There was a marginally significant amount of variation explained by the interaction between exposure and family (*R*^2^ = 0.018, adjusted *P* = 0.077). Using family-wise PERMANOVA tests (Table 2, within), we determined that Picornaviridae was the only case in which surface exposure had a marginally significant effect on grouping within this family (*R*^2^ = 0.031, adjusted *P* = 0.079). This suggests that the effect of surface-exposure is not a result of confounding due to variation among virus families, *i.e.*, because Picornaviridae have distinct fingerprints from other viruses. However, we need to be cautious that Picornaviridae is also one of the largest families in our dataset (second only to Flaviviridae) so the absence of significant associations within other families may be due to a lack of statistical power, rather than a genuine biological difference.

**Fig 5:**
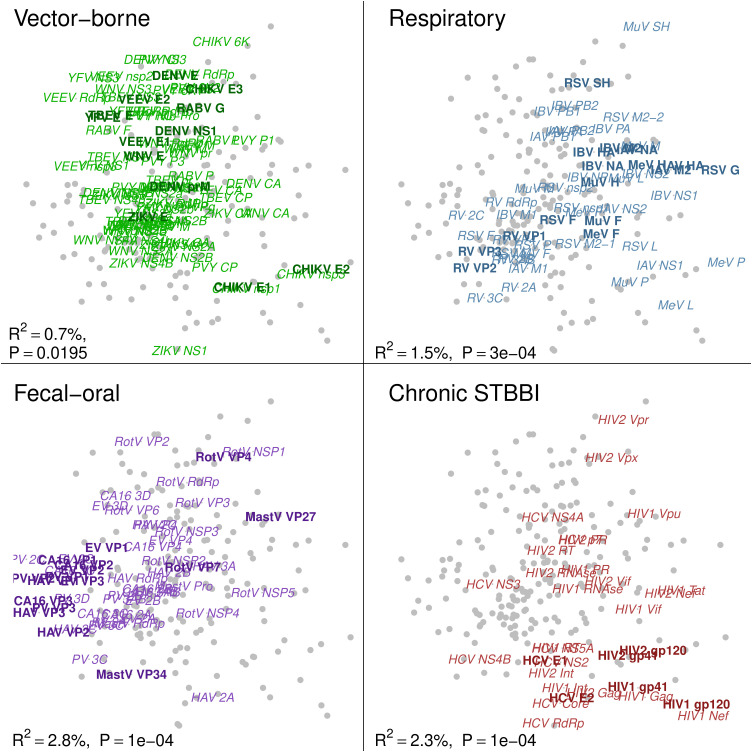
Association between evolutionary fingerprints and modes of transmission. Each plot depicts the same MDS projection of the residualized Wasserstein distance matrix as Fig 4, except that proteins associated with viruses are highlighted with coloured labels for different modes of transmission. Bold and italicized labels differentiate surface-exposed and non-exposed proteins. Results from one-versus-rest PERMANOVA tests on the raw Wasserstein distances for each group are summarized in the lower-left corner of each plot. ‘Vector-borne’ corresponds to viruses that are transmitted by mosquitoes, ticks or aphids. STBBI = sexually transmitted or blood-borne infections. Points corresponding to Borna disease virus (BDV) were not labelled as its mode of transmission is not well characterized [91]. An additional plot for plant viruses transmitted by contact, *e.g.*, contaminated tools, is provided as Supporting Information (S11 Fig) due to space constraints.

Overall, these results imply that virus-level attributes may have a greater influence on selective regimes than the characteristics of individual proteins encoded by a virus. We evaluated this hypothesis by annotating evolutionary fingerprints with the predominant modes of transmission of their respective viruses. This yields some visually distinguishable groups (Fig 5). For example, sexually transmitted and blood-borne infections (STBBI) tend to have broader fingerprints, whereas the fingerprints for proteins associated with vector-borne viruses tend to be more compact. Collectively, fingerprints differed significantly among modes of transmission (PERMANOVA, $P\leq 10^{-5}$), explaining 6.1% of variation in Wasserstein distances (15.9% of variation after removing confounders). When we added surface exposure to this permutation test, we obtained a significant interaction between mode of transmission and exposure (PERMANOVA *R*^2^ = 0.012, *P* = 0.014). The main effect of transmission mode remained significant (*R*^2^ = 0.061, *P* < 10^-5^), while exposure itself was marginally significant (*R*^2^ = 0.004, *P* = 0.04).

We need to be cautious about interpreting this outcome, however, because transmission modes are confounded with taxonomy, *i.e.*, viruses in the same family usually have the same mode of transmission. There are some specific features that are consistent with the hypothesis that fingerprints are shaped by modes of transmission irrespective of taxonomy. For instance, hepatitis C virus (HCV) is a member of Flaviviridae but a majority of its fingerprints are more similar to other STBBIs (HIV-1 and HIV-2, Retroviridae) than the other flaviviruses, which are vector-borne. This is the most evident with respect to the second MDS coordinate on residualized distances (Fig 4), where there is a significant difference between fingerprints from HCV and other flaviviruses (Wilcoxon test, *P* = 0.0026), but not between HCV and Retroviridae (*P* = 0.32). Fingerprints from potato virus Y (Potyviridae), which is transmitted by aphids, and the highly zoonotic rabies virus (Rhabdoviridae) are similar to those vector-borne flaviviruses, although they also overlap with respiratory and fecal-oral groups. Additionally, the group of respiratory viruses covers several different virus families. Fingerprints for measles and mumps virus (Paramyxoviridae) and respiratory syncytial virus (Pneumoviridae) are similar to those of influenza viruses (Orthomyxoviridae; Fig 5). On the other hand, fingerprints associated with rhinovirus (RV) cluster more with other members of Picornaviridae, suggesting that characteristics other than transmission mode (such as virion structure, see above) exert a greater effect in this case.

## Discussion

The scientific literature is rich with examples of strong positive selection acting on specific sites of surface-exposed proteins of RNA viruses [7,8,10,47,48]. This selection is often attributed to non-synonymous mutations that escape recognition and binding by neutralizing antibodies. As a result, comparative studies of selection in viruses are frequently framed as a comparison between surface-exposed and non-exposed proteins [9,19,49]. Although the humoral immune response is a major cause of selection in the host environment, there are many other factors that also contribute to virus evolution. The cellular immune response, for instance, can act on any protein produced from the virus genome, and is also an important part of the adaptive immune response. For example, an analysis of a large longitudinal dataset of HIV-1 genome sequences within a single subject [50] found that about half of codon sites under directional or diversifying selection were associated with genes other than *env*, and about 40% of these sites were associated with cytotoxic T-cell lymphocyte epitopes. Our results demonstrate that the effect of surface exposure on the selection profiles of virus proteins is not so simple. Contrary to expectations, surface-exposed proteins did not form a distinct cluster when we projected virus gene alignments into a space that represents the differences in their respective distributions of site-specific *dN* and *dS* rates, *i.e.*, their evolutionary fingerprints. A significant and substantial effect of surface exposure was resolved only when we stratified proteins by their associations with enveloped or non-enveloped viruses.

### What is different about Picornaviruses?

Our finding of a significant interaction between exposure and enveloped factors was largely driven by a cluster of fingerprints representing surface-exposed proteins from non-enveloped viruses (Fig 4). These viruses were predominantly members of the Picornaviridae family. Picornaviruses have a spherical capsid with no distinct spike structures [51]. The surface-exposed proteins are the major capsid proteins VP1, VP2 and VP3. These proteins are structurally similar, sharing a common β-sandwich jelly roll fold, and contribute jointly to the formation of the outer capsid in equal numbers. VP1 forms most of the ‘canyon’ at the centre of the pentameric subunit that is responsible for host receptor-binding. Moreover, VP1 is highly exposed with variable loops that are important targets for neutralizing antibodies [52,53]. Based on their evolutionary fingerprints, however, these major capsid proteins were subject to lower site-specific *dN*/*dS*, either due to stronger purifying selection or weaker diversifying selection. This is the opposite of the expected trend. A possible explanation is that maintaining the structural and functional integrity of the virus capsid may involve a greater number of conserved protein-protein interactions in this virus family. For example, a recent study [54] found that naturally-occurring viral capsids have more protein-protein interactions than synthetically-engineered capsids, or capsid-like structures produced by the overexpression of a structural protein. In contrast, surface-exposed proteins that are embedded in a viral envelope may have comparably fewer protein-protein interactions.

There were a few outliers relative to this cluster of surface-exposed, non-enveloped proteins under strong purifying selection: specifically, VP27 from mamastrovirus (Astroviridae), and VP4 and VP7 from rotavirus A (Sedoreoviridae). VP27 forms a dimer with VP25, a shorter gene product derived from the same precursor polypeptide following proteolytic cleavage by trypsin [55]. This precursor is encoded by the hypervariable central region of the genome [56]. The resulting dimer forms a spike that protrudes from the core structure of the virus particle and is highly antigenic [57]. Rotavirus is a double-shelled virus with inner and intermediate capsid structures that are surrounded by an outer shell comprising the hemagglutinin VP4 and glycoprotein VP7. Both VP4 and VP7 independently induce neutralizing antibodies and have been used to define rotavirus serotypes [58]. Moreover, VP4 is cleaved into two subunits that assemble into a spike that produces from the capsid surface [59]. These characteristics imply that localization of these proteins into external structures, *i.e.*, a spike or outer shell, may reduce the number of essential protein-protein contacts relative to the major capsid proteins of Picornaviruses.

### Differences among viruses, not proteins

The main hypothesis evaluated in this study implicitly assumes that the selective regimen imposed on a virus protein is shaped by that protein’s function. A protein that is exposed on the surface of the virus particle is generally responsible for recognizing and binding to host receptors, or mediating viral entry into the host cell. Consequently, our expectation was that the evolutionary fingerprints, as detailed quantitative measures of selective regimens, should cluster into groups of exposed and non-exposed proteins, irrespective of their taxonomic relationships. Instead, we found that the distribution of fingerprints was more significantly associated with characteristics of the virus, specifically whether it is enveloped, its taxonomic family (S10 Fig) and its mode of transmission (Fig 5). These effects are difficult to separate; for example, members of the same virus family usually have the same mode of transmission. Put another way, the effect of transmission mode on evolutionary fingerprints is confounded by phylogenetic relationships.

Typically, comparative studies that aim to find associations between evolving characteristics and external factors must contend with confounding due to phylogenetic non-independence. For example, many virus proteins contain linear epitopes (short amino acid motifs) that lead to lysis of the infected cell when these peptides are recognized by human leukocyte antigen (HLA) molecules and presented to cytotoxic T lymphocytes (CTLs). CTL escape mutations can be identified from associations between polymorphisms in viral epitopes and the HLA genotypes of hosts. However, the same mutation may appear in a group of infections because they share a recent common ancestor, and not because they were exposed to similar immune environments [60]. Several phylogenetic methods have been developed to isolate the true association from confounding due to identity by descent, such as independent contrasts [61] and phylogenetic regression [62].

Applying these concepts to evolutionary fingerprints is not straight-forward. First, we are comparing quantities derived from alignments of genes that usually share no evolutionary homology. For example, influenza A virus neuraminidase (IAV NA) does not share a common ancestor with coxsackievirus A16 major capsid protein VP3. This is also generally true when the genes derive from the same virus, *e.g.*, IAV NA and PB2. On the other hand, our dataset contains some genes that are distantly related. For example, VP3 is represented from five different members of Picornaviridae. The fingerprints for VP3 form a tight cluster along with VP1 and VP2 capsid proteins, which share a high degree of structural similarity with VP3 and may be the result of gene duplication [63]. However, these duplication events would be associated with the common ancestor of Picornaviridae, with an evolutionary history that is likely on the scale of millions of years [64], which dwarfs the time scale of the genetic variability from which our evolutionary fingerprints were derived. Furthermore, gene duplication cannot explain why fingerprints are so similar across all proteins encoded by the same virus, such as rotavirus A or influenza B virus (Fig 4).

Second, there is limited precedence for thinking about evolutionary fingerprints as an evolving character state [23]. An evolutionary fingerprint is a probabilistic representation of variation in non-synonymous and synonymous substitution rates among codon sites in a protein-coding gene (Fig 1). There is precedent, however, for modeling variation in the overall rate of evolution as an evolving character state with relaxed molecular clock models. For example, autocorrelated clock models constrain the rate associated with a branch in the phylogeny to be similar to the rate of its parent branch [65]. This rate variation is generally assumed to be driven by changes in the environment over time. Shifts in an evolutionary fingerprint, which is essentially an ensemble of rate categories for non-synonymous and synonymous codon substitutions, can thus be viewed as an extension of this model. It is intuitive that shifts in non-synonymous rates can arise from changing targets of selection on proteins as the virus moves through different host environments.

There is also abundant evidence that selection on synonymous variation can affect many sites within protein-coding genes [66–68]. We note that persistent variation among sites in both non-synonymous and synonymous substitution rates are captured by evolutionary fingerprints. On the other hand, comparative methods to detect episodic changes in site-specific synonymous rates over time are less established [22]. Selection on synonymous substitutions can be attributed in part to conserved secondary structures in RNA virus genomes [69]. For instance, Nicholson and White [70] observed that RNA viruses tend to fall into one of two categories: those with structurally compact genomes due to long-range RNA interactions, *e.g.*, hepatitis C virus, and those with elongated conformations with localized secondary RNA structures, *e.g.*, HIV-1. Another potential source of selection on synonymous variation is adapting codon usage in the virus genome to the tRNA repertoire of the current host species [71]. Codon usage biases can vary substantially among potential host species [72]. Thus, the zoonotic transfer of a virus to a new host species may induce shifts in synonymous rates.

This is not the first time that differences in selection among groups of viruses have been documented. For example, Lin *et al.* [6] noted that RNA viruses from the same family tended to have similar gene-wide *dN*/*dS* ratios, although they did not provide a statistical test to support this observation. They suggested that this pattern could be caused by shared characteristics such as transmission dynamics, host environments, genome structure or effective population size. Similarly, Woelk and Holmes [9] reported that selection on virus proteins was associated with modes of transmission. They observed that mean *dN*/*dS* ratios for genes encoding surface-exposed proteins were significantly lower for vector-borne than non-vector-borne viruses. In contrast, there was no significant difference for genes encoding internal structural proteins.

Our study incorporates variation in substitution rates among sites, expanding comparisons from a single dimension (the mean *dN*/*dS* ratio) to a multi-dimensional space induced by the Wasserstein distance between evolutionary fingerprints. This enabled us to resolve significant differences in patterns of selection among groups of proteins defined by exposure, taxonomy, and transmission. One of the interesting results from this analysis is that fingerprints associated with STBBIs (HIV-1, HIV-2, and HCV) tend to be ‘flatter’, with more variation in site-specific rates. Another feature of this group is that these viruses establish persistent chronic infections. This association suggests that prolonged exposure to the immune response specific to each host increases the variation in substitution rates among sites. Conversely, the selective regimes acting on acute viral infections may become averaged out as the virus is transmitted rapidly through a succession of hosts [18].

### Inclusion of plant viruses

In contrast to previous work [6,9,19], we chose to include viruses that infect plant hosts. The four plant viruses in our dataset (potato viruses X and Y, tobacco mosaic virus, and apple stem pitting virus) have a filamentous or rod-like structure formed by the oligomeric assembly of coat protein around genomic RNA. Unlike vertebrates, plants do not have circulating immune cells and rely on intrinsic innate immunity, along with systemic signals from infected cells [73,74]. Innate antiviral mechanisms in plants include physical barriers and induced responses such as the production of phytoalexins and activation of defense genes [75]. Plants encode pattern recognition receptor proteins that bind to conserved motifs in pathogen-derived molecules, activating downstream defense signaling pathways that can alter gene expression within minutes of infection [73,76]. Previous infections can precondition plants to raise a faster and more robust response to secondary infections, known as innate immune memory [75,77]. Another defense mechanism in plants involves small interfering RNAs (siRNAs) that suppress virus gene expression. These siRNA are processed from host-mediated cleavage of double-stranded viral RNA produced during virus replication [78].

Our study focuses on detecting both purifying and diversifying selection. For viruses, diversifying selection is driven by the transmission of lineages through different host environments. In vertebrates, the components of the adaptive immune response are encoded by highly variable regions of the genome including the major histocompatibility loci. Neutralizing antibodies are known to be an important driver of diversifying selection on specific sites in some surface-exposed viral proteins, such as IAV hemagglutinin [8] and HIV-1 gp120 [79]. As noted above, these observations have influenced the comparative study of selection in human viruses [9,19]. It is less clear whether a similar trend could be expected to influence selection on protein-coding genes in plant viruses. Comparative studies of plant viruses have found sites under significant diversifying selection in both coat proteins and nonstructural proteins [80–82]. Moreover, Murray *et al.* [83] observed that genes encoding suppressors of host siRNA — generally genes other than those encoding coat/capsid proteins — had the most evidence of episodic diversifying selection among genes encoded by the majority of plant viruses in their study.

Given the substantial differences in immune systems between plant and vertebrate hosts, it is not obvious which proteins from plant viruses should be categorized alongside the surface-exposed proteins of viruses that infect animal hosts. However, any combination of classifying the coat proteins or viral suppressors of RNA silencing (VSRs) as either surface-exposed or non-exposed, or omitting all plant viruses from the data entirely, had little impact on our permutation test results on evolutionary fingerprints (Supporting Information, S3 Table). In addition, the mean dN/dS values associated with coat proteins or VSRs were not significantly different from other plant virus proteins (Wilcoxon test, *P* = 0.87). Compared to viruses infecting vertebrate, especially human, hosts, there has been less research on characterizing patterns of selection in plant viruses. As growing numbers of full-length genomes become available for plant viruses [84], we anticipate that this will be an interesting area for further work.

### Comparison to previous work

Previous studies testing the hypothesis that surface-exposed proteins evolve under a different selective regime than other virus proteins have largely been based on the McDonald-Kreitman (MK) test [85]. This test compares the observed numbers of non-synonymous and synonymous substitutions (differences, $D_{N}$ and $D_{S}$) against the corresponding numbers of polymorphisms ($P_{N}$ and $P_{S}$). Sites where $D_{N}/D_{S}$ significantly exceeds $P_{N}/P_{S}$ are interpreted to be cases of adaptive evolution. The significance of this outcome is generally determined by a $\chi^{2}$ test or Fisher’s exact test on the 2×2 contingency table of differences and polymorphisms.

Bhatt *et al.* [20] (BKP10) extended the MK test to account for different types of polymorphisms, and then applied this test to 95 protein-coding gene alignments representing 82 virus species. Substitutions were identified as differences from a reference sequence, which was the sample with the earliest collection date. They did not explicitly test for the effect of surface exposure. However, the published materials provided sufficient information to carry out this test. There was no association between significant adaptive evolution (MK test *P* < 0.05/95) and surface exposure, irrespective of whether plant viruses were excluded (Fisher’s exact test, odds ratio 1.41, *P* = 0.63) or if coat proteins from plant viruses (*n* = 24) were classified as surface-exposed (odds ratio 1.41, *P* = 0.51). Foregoing adjustment for multiple comparisons did not change this outcome (*P* > 0.22). Subsequently, Bhatt and colleagues performed a follow-up study [49] (BKP11) that focused on proteins of human influenza A virus subtypes H3N2 and H1N1 over three decades of evolution. They observed stronger adaptive evolution in hemagglutinin and neuraminidase than the other IAV proteins for both subtypes. In their previous study (BKP10), significant adaptive evolution was not detected for hemagglutinin for human IAV H3N2. However, the number of H3N2 sequences used in BKP10 was also much smaller (*n* = 50) than BKP11 (*n* = 1,674).

Kistler and Bedford [19] (KB23) extended BKP10’s method to account for multiple substitutions at the same site. This was accomplished by updating the reference sequence at successive intervals, taking the consensus of the preceding time interval as the new reference. Consequently, their approach required the virus sequences to be related by a ladder-like tree, with short-lived lineages derived from a single trunk lineage over time. Substitutions are measured relative to a reference genome in the past, and polymorphisms are measured for lineages sampled within a given time interval before most of them go extinct. KB23 applied this method to 19 RNA and DNA virus species, some of which were represented by multiple subtypes. They found significant adaptive evolution in 10 out of 28 receptor-binding proteins (8 out of 18 from enveloped viruses). In contrast, adaptive evolution was not detected in any of 27 polymerase proteins.

A key difference between these studies and the present work is that they are measuring different forms of positive selection, *i.e.*, selection promoting amino acid replacements. The *dN*/*dS* method employed in our study measures diversifying positive selection, which arises when co-circulating lineages are transmitted through different selective host environments. This method is not designed to detect directional positive selection from longitudinal samples of a single population [17]. Directional selection in a population is generally a transient phenomenon: eventually, the mutations with a selective advantage in the current environment reach fixation, and subsequent mutations at those sites are removed by purifying selection. The analogous scenario for an infectious disease is one where all lineages are exposed to the same selective environment across hosts. We made efforts to avoid this scenario *a priori* while collecting data for this study, *e.g.*, targeting influenza A virus (IAV) subtype H9N2 sequences from avian hosts rather than human IAV subtype H3N2.

The MK-type methods employed by BKP11 and KB23 are implicitly designed for longitudinal samples of a single dominant (trunk) lineage over a substantial amount of time. In other words, sequences are assumed to be related by a ladder-like phylogeny [29]. These requirements limit the range of viruses to which the method can be applied [19]. On the other hand, MK-type methods are especially well-suited for respiratory viruses like human influenza A virus. Seasonal human IAV outbreaks lead to a proliferation of lineages from the trunk, until the host population develops a short-lived immune response that restricts further infection by any strain [29]. Over the long term, the selective environment changes as the host population’s immune repertoire adapts to current and past exposures [86], which drives ongoing virus adaptation. These evolutionary and immunological dynamics may be a characteristic feature of respiratory viruses due to the properties of the respiratory tract [87].

### Limitations and future directions

Evolutionary fingerprints can provide a useful framework for comparing unrelated protein-coding genes with respect to how their diversities have been shaped by selection [23]. This method enables the investigator to compare site-specific rate estimates between unrelated genes through the common framework of their latent distributions. However, evolutionary fingerprinting is challenging to implement in practice. In this study, we have observed that fingerprints are sensitive to the amount of genetic variation in the data, which is quantified by tree length (also known as phylogenetic diversity). The length of the alignment has the same effect because it increases the sample size with respect to substitution events. Hence, both quantities determine the extent by which the likelihood can reshape the prior distribution. The original developers of the evolutionary fingerprinting method [23] also noted that resolving the distribution of site-specific rates was affected by sample size and genetic divergence. Instead of a posterior distribution on a fixed grid of *dN* and *dS* values used in the FUBAR method, however, variation in substitution rates among sites was modeled by a general bivariate discrete distribution. This distribution comprises *K* rate classes that are each represented by three parameters: the class probability $p_{k}$ and class-specific $dN_{k}$ and $dS_{k}$ rates. These parameters were estimated from the data by maximum likelihood under the constraints $\sum_{k=1}^{K}p_{k}=1$ and $\sum_{k=1}^{K}dS_{k}/K=1$. Furthermore, the issue of sample size was mitigated by approximating the posterior distribution around these point estimates with sampling-importance-resampling [23]. Our findings indicate that even adopting a fully Bayesian approach is not sufficient for rapidly-evolving RNA viruses. In contrast, the study by Murrell *et al.* [25], on which we modeled our approach to evolutionary fingerprinting, used this method to characterize the coevolution between primate retroviruses and host restriction factors. Because their analysis comprised genes encoding restriction factors from the same set of mammalian genomes, it was not necessary to normalize the amount of genetic variation in their data.

We developed two methods to remove the confounding effect of genetic variation from visual representations of fingerprints: first, by regressing out the alignment and tree lengths from the multidimensional scaling (MDS) coordinates and generating a new distance matrix from the residual values; and second by normalizing the amount of genetic variation among datasets by downsampling sequences. Using either residualization or downsampling methods obtained similar results, despite large differences in sampled genetic variation among RNA viruses. One of the disadvantages of downsampling is that some alignments were not sufficiently long to sample a specific number of codons without replacement, causing some alignments to be discarded. In addition, normalizing the amount of genetic variation to a consistent level could also drastically reduce our ability to detect sites under diversifying selection, or the precision to which an evolutionary fingerprint could be resolved. Residualization allows one to retain the entire content of the original sequence alignments. On the other hand, a limitation of residualization is that regression assumes a linear relationship between the MDS coordinates and confounding variables.

Another limitation of evolutionary fingerprinting is that it relies on a codon substitution model that is time homogeneous, *i.e.*, with constant *dN* and *dS* rates at each codon site. This means that selection pressures must be consistently maintained across multiple lineages over time to be detectable. However, site-specific selection pressures can change over time. This shift can be detected by branch-site or episodic selection models [88], although there is an inherent limit to their statistical power. Extending the evolutionary fingerprint to accommodate time-heterogeneous rates remains an open problem. Furthermore, fingerprinting causes the variation in *dN* and *dS* rates among sites to be collapsed into a bivariate distribution, which discards information about the relative locations of these codon sites in the gene. For example, positively-selected sites tend to cluster in the tertiary structures of proteins [89], and this rate variation is associated with solvent accessibility and residue contacts [90]. Incorporating positional information is difficult because of variation in sequence lengths among different genes. One possible solution might be to allow a gene to have multiple bivariate *dN*-*dS* distributions. These distributions could be mapped to codon sites by a hidden Markov model. We would then need to extend the Wasserstein distance to compare fixed lengths of subsequences of fingerprints between two gene alignments. Extending the evolutionary fingerprinting method in these directions will be a compelling area for future research.

## Supporting information

S1 Fig Distributions of alignment and tree lengths among gene alignments. These histograms summarize the distributions of (A) alignment lengths (number of codon sites) and tree lengths (expected number of substitutions per nucleotide site) before (B) and after (C) pruning the longest terminal branches to reduce the tree length to the target range from 0.5 to 2.0 (depicted by shaded region). Note that some tree lengths after pruning (C) were slightly above our target of 2.0 substitutions per nucleotide site because removing the next branch would result in a tree length that was even further from this target.
(PDF)

S2 Fig Accuracy in estimating site-specific dN/dS improves with tree length. Sequence alignments were simulated from a coalescent tree that was rescaled to different lengths, expressed in units of expected substitutions per codon site. These can be converted to expected substitutions per *nucleotide* site by dividing the value by 3, *e.g.*, 6.0 = 2.0 expected substitutions per nucleotide site. We calculated the root mean square error (RMSE) between the known dN/dS values and the estimated values across codon sites using two methods: FUBAR (left) and FEL (fixed effects likelihood, right). Each point represents the RMSE for one of ten replicates per tree length, for varying lengths. Larger points connected by line segments represent the mean RMSE over replicates. A dashed red line represents the proportion of alignments with a tree length below the threshold (*x*-axis) after conversion from nucleotide to codon units. This indicates that raising the threshold is limited by the availability of alignments with sufficient genetic variation. The shaded region represents the target range of tree lengths (0.5 to 2.0 expected substitutions per nucleotide site) used for downsampling.
(PDF)

S3 Fig Correlation between Wasserstein distances and the difference in log-transformed alignment lengths. Each point in this scatterplot represents a pairwise comparison between two gene alignments. The *x*-axis represents the absolute difference in the log-transformed lengths (number of codons) between two gene alignments. The *y*-axis represents the Wasserstein distance between the evolutionary fingerprints of the alignments. Contours from a bivariate normal kernel density is superimposed on the plot to clarify the distribution of points in the denser regions.
(PNG)

S4 FigEffect of tree length of evolutionary fingerprints.Each fingerprint depicts the posterior probability distribution over a fixed grid of 20×20 *dS* (x-axis) and *dN* (y-axis) values. Darker cell shades correspond to higher posterior probabilities. These fingerprints were derived from progressively smaller numbers of HIV-1 *env* sequences, resulting in shorter tree lengths (as measured by the expected number of nucleotide substitutions, upper left).(PNG)

S5 Fig Confounding of Wasserstein distances and genetic variation. Both plots represent the same multidimensional scaling (MDS) projection of the Wasserstein distance matrix for the full gene alignments, *i.e.*, without tree pruning. Point area is scaled in proportion to alignment length (left) or with an affine transformation (fixed minimum area) on tree length (right).
(PDF)

S6 Fig Clustering of fingerprints derived from random samples of alignments. Each plot is derived from the same multidimensional scaling projection of the Wasserstein distance matrix for samples of 100 codon sites from gene alignments. Points (open circles, black) corresponding to the 10 replicate samples from a given gene alignment are highlighted for a random selection of viruses and protein-coding genes (labels).
(PDF)

S7 Fig Multidimensional scaling (MDS) projections of corrected distances. These plots depict MDS projections of the Wasserstein distance matrices in which the confounding effects of genetic variation were removed by one of two methods: (1) by regressing out the effects of alignment length and tree length (residualized, top row), or; (2) by progressively removing sequences associated with the longest terminal branches in the phylogeny, and sampling codon sites from the remaining sequences at random without replacement (bottom row). Each point represents a single gene alignment or the centroid of 10 random samples of 100 codon sites from each alignment; alignments fewer than 100 codon in length were excluded from the latter. Point area is scaled to the number of codon sites (alignment length) in the original alignment, or to tree length (as in S5 Fig).
(PNG)

S8 FigMultidimensional scaling plots of Wasserstein distances from downsampled alignments.Each point represents the centroid of ten replicate samples of *L* = 100 codon sites from a gene alignment that was downsampled to normalize tree lengths. The x− and *y*-axes capture 51% and 15% of the variance, respectively. Replicate samples formed distinct clusters when visualizing the entire distance matrix (S6 Fig). Requiring a minimum of 100 codons excluded 41 (16.8%) out of 244 gene alignments from our analysis; the median alignment length was 255.5 (interquartile range, IQR: 130−466 codons; S1A Fig). Proteins from enveloped viruses are labeled on the left side, and non-enveloped viruses on the right. Each point is labeled with the respective virus and protein, and styled to indicate surface-exposed and non-exposed states as in Fig 4. Results from PERMANOVA are provided in Supporting Information (S2 Table).(PDF)

S9 Fig Reducing alignment length from 100 to 50 codons does not qualitatively affect results. Like S8 Fig, these plots depict the multidimensional scaling projection of the Wasserstein distance matrix for downsampled alignments. Each point represents the centroid of 10 random samples of 50 codon sites from each alignment, labeled with abbreviations for virus and gene product. Labels are highlighted in bold for surface-exposed proteins and italics for enveloped viruses. The distance matrices of centroids for *L* = 50 and *L* = 100 codons were significantly correlated (Mantel test, *r* = 0.96, *P* < 10^-5^). Results from PERMANOVA tests are provided in Supporting Information (S2 Table).
(PDF)

S10 Fig Clustering of virus proteins by family. Each plot depicts the same MDS projection as Fig 4, except points are highlighted for proteins associated with viruses in one of the six families with multiple species in our data set. Points are filled for surface-exposed proteins, and open otherwise.
(PDF)

S11 Fig Fingerprints for plant viruses transmitted by contact. Each plot depicts the same MDS projection as Fig 4, except points are highlighted for proteins associated with viruses with different modes of transmission. The four plots on the left-hand side reproduce Fig 5, with filled circles for surface-exposed proteins and open circles otherwise. The remaining plot highlights the evolutionary fingerprints associated with proteins from plant viruses that are predominantly transmitted by direct contact, *e.g.*, contaminated farm equipment or grafting. Fingerprints were not significantly different for this group versus all other data (PERMANOVA *R*^2^ < 0.01, *P* = 0.23).
(PDF)

S1 Table Summary of viral protein names and characteristics. *Virus* = abbreviated virus name (see Table 1). Followed by the Genbank accession of the reference genome used for determining gene coordinates; if the genome is segmented, then the accessions are provided alongside the respective gene products (*Protein*). *Abbrv* = abbreviation of protein name for figures. *Ex?* = is the protein classified as surface-exposed? *L* = the number of codon sites prior to normalizing alignment lengths by random sampling. *Coords* = nucleotide coordinates in reference genome, determined by pairwise alignment of the consensus sequence of the curated data set. Multiple ranges are given for products of spliced exons, to remove indels with respect to the reference, or when an overlapping open reading frame was removed from the alignment. *N* = the number of sequences after normalizing tree length. *TL* = total tree length (expected substitutions per nucleotide site) after normalization by pruning.
(PDF)

S2 Table PERMANOVA results for downsampled datasets. Wasserstein distances were averaged between replicates for every pair of virus proteins to obtain a reduced distance matrix for the centroids. Tests were run for 10,000 permutations using the *adonis2* function in the R package *vegan*. The term ‘exposed:enveloped’ represents the interaction between the respective factors. *R*^2^(%) represents the percentage of variation explained by each factor. Results are provided for Wasserstein distance matrices calculated for fingerprints on both log-offset and integer indexed coordinates on the grid of *dS* and *dN* rates.
(PDF)

S3 Table PERMANOVA results for different treatments of proteins associated with plant viruses. The following proteins were identified as viral suppressors of RNA silencing (VSRs) according to the literature: TGB1 for potato virus X [Aguilar *et al.* (2015) J Virol 89(4): 2090–2103], HC-Pro and NIa-VPg for potato virus Y [Cheng and Wang (2017) J Virol 91: e01478-16], RP for tobacco mosaic virus [Vogler *et al.* (2007) J Virol 81(19): 10379–10388], and CP for apple stem pitting virus [Ma *et al.* (2019) Virology J 16: 20]. Tests were run for 10,000 permutations on the residualized Wasserstein distance matrix using the *adonis2* function in the R package *vegan*. The term ‘exposed:enveloped’ represents the interaction between the respective factors. *R*^2^(%) represents the percentage of variation explained by each factor.
(PDF)
