# Supplementary figures and images for: Selection profiles in RNA viruses reflect the characteristics of viruses more than individual proteins
Source: PLoS Pathog. 2026 Jul 24;22(7):e1014457. doi: 10.1371/journal.ppat.1014457 (PMC13432152; doi:10.1371/journal.ppat.1014457)

**A**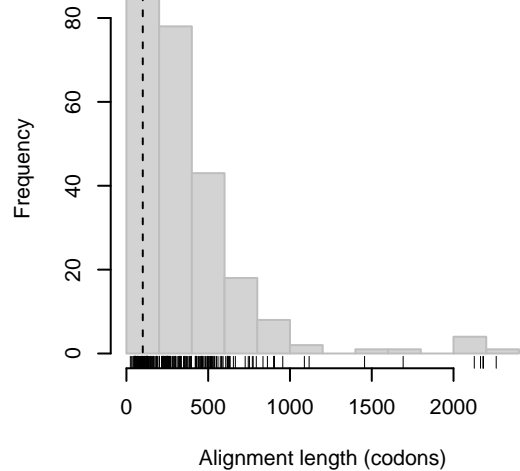**B**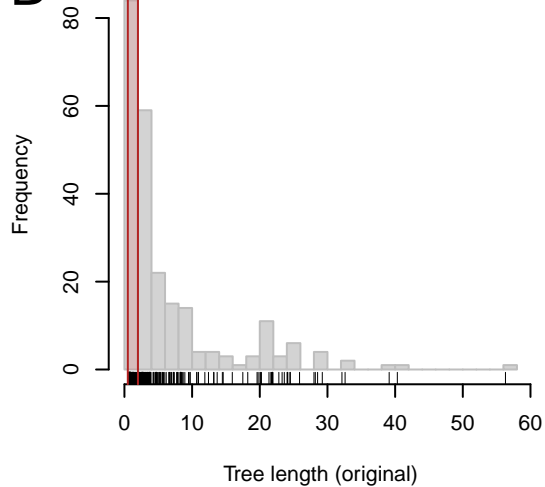**C**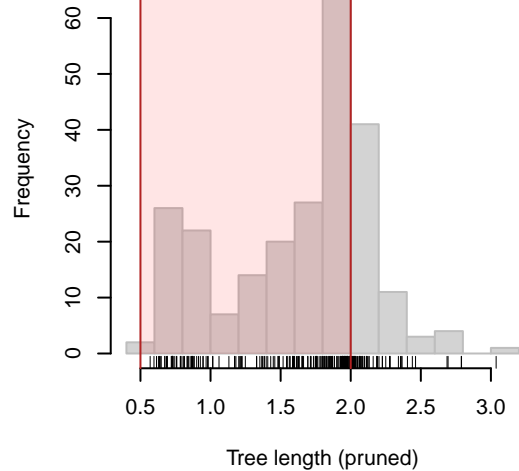

Supplement: S1 Fig — These histograms summarize the distributions of (A) alignment lengths (number of codon sites) and tree lengths (expected number of substitutions per nucleotide site) before (B) and after (C) pruning the longest terminal branches to reduce the tree length to the target range from 0.5 to 2.0 (depicted by shaded region). Note that some tree lengths after pruning (C) were slightly above our target of 2.0 substitutions per nucleotide site because removing the next branch would result in a tree length that was even further from this target. (PDF) [file ppat.1014457.s001.pdf]

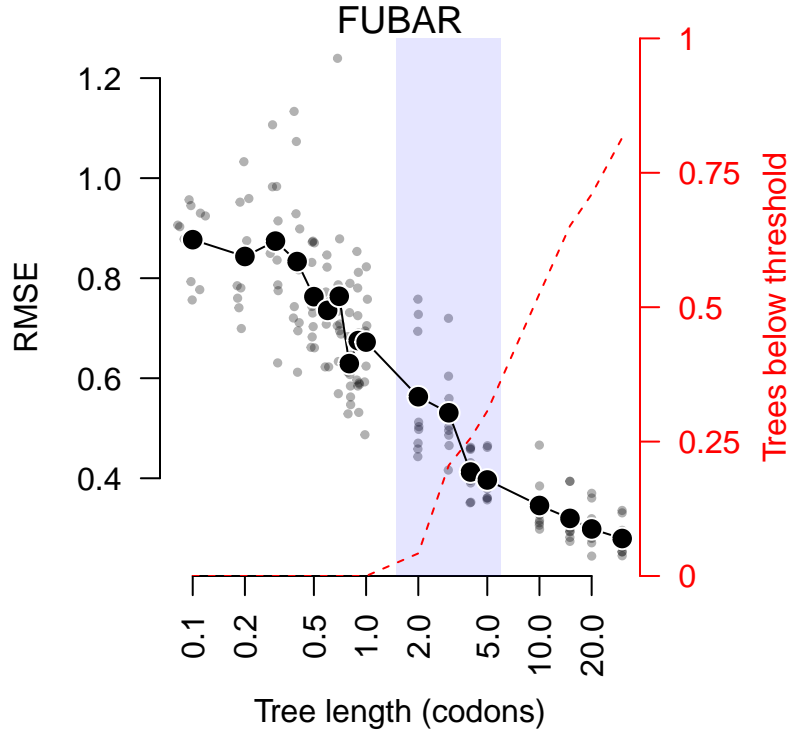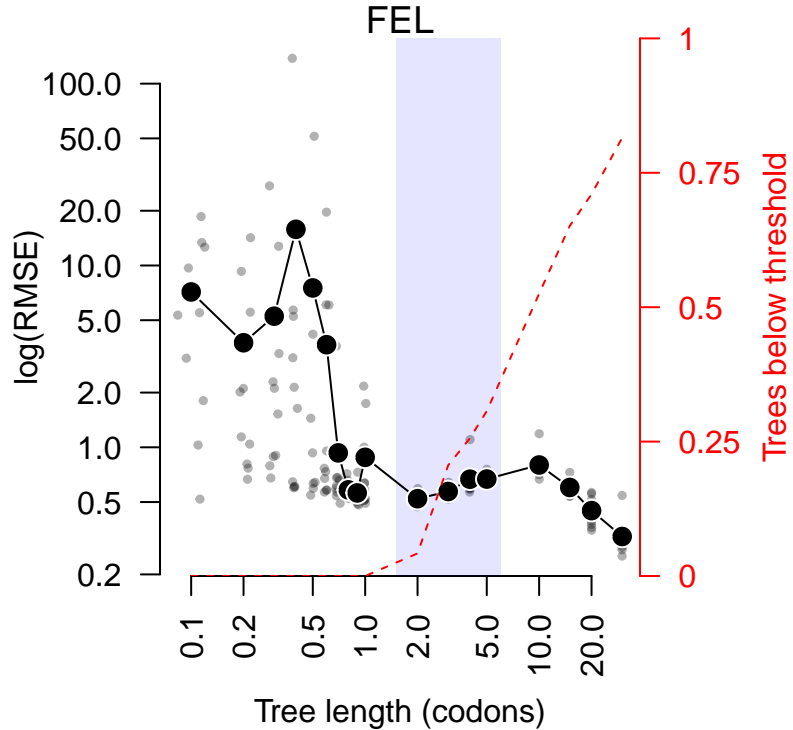

Supplement: S2 Fig — Sequence alignments were simulated from a coalescent tree that was rescaled to different lengths, expressed in units of expected substitutions per codon site. These can be converted to expected substitutions per nucleotide site by dividing the value by 3, e.g., 6.0 = 2.0 expected substitutions per nucleotide site. We calculated the root mean square error (RMSE) between the known dN/dS values and the estimated values across codon sites using two methods: FUBAR (left) and FEL (fixed effects likelihood, right). Each point represents the RMSE for one of ten replicates per tree length, for varying lengths. Larger points connected by line segments represent the mean RMSE over replicates. A dashed red line represents the proportion of alignments with a tree length below the threshold (x-axis) after conversion from nucleotide to codon units. This indicates that raising the threshold is limited by the availability of alignments with sufficient genetic variation. The shaded region represents the target range of tree lengths (0.5 to 2.0 expected substitutions per nucleotide site) used for downsampling. (PDF) [file ppat.1014457.s002.pdf]

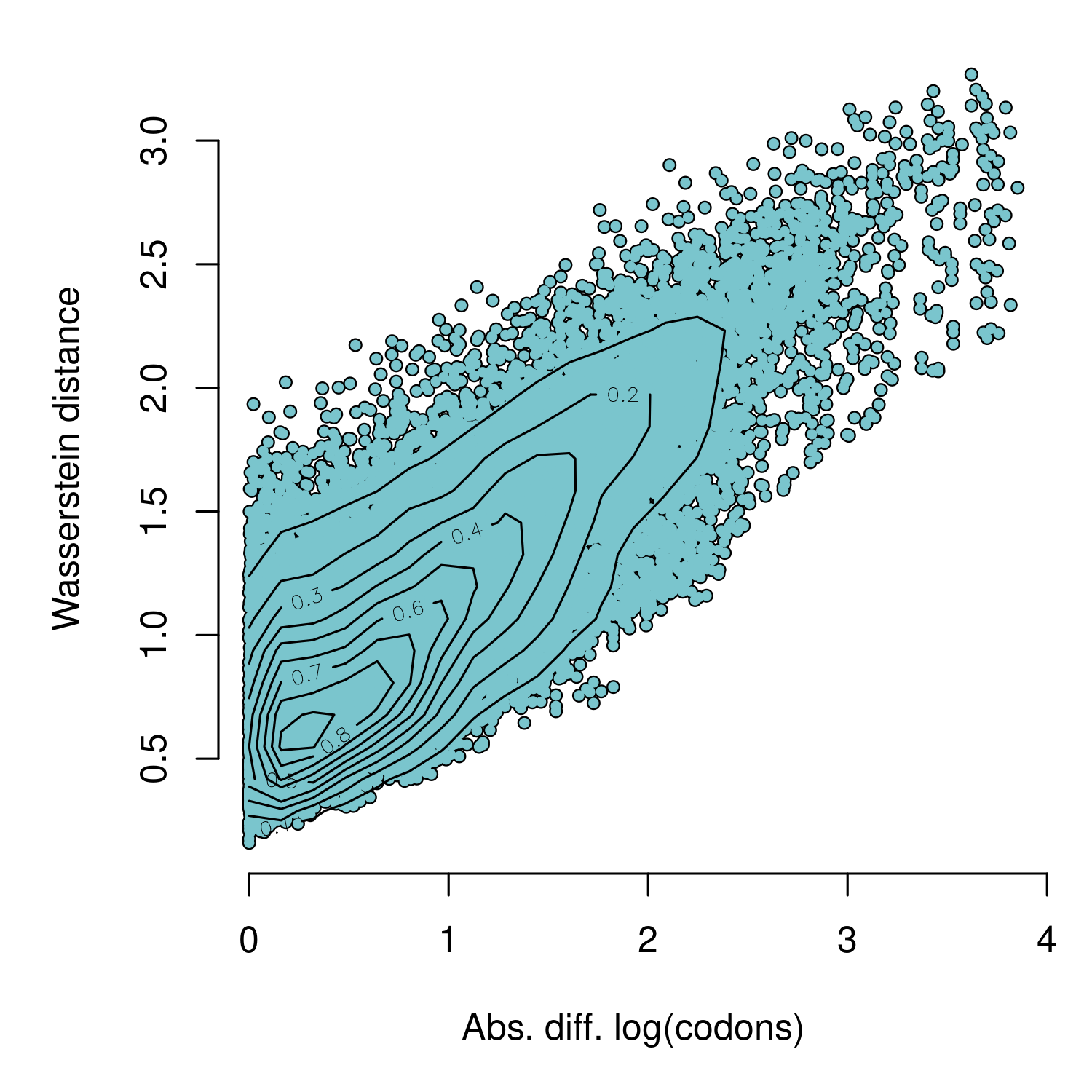

Supplement: S3 Fig — Each point in this scatterplot represents a pairwise comparison between two gene alignments. The x-axis represents the absolute difference in the log-transformed lengths (number of codons) between two gene alignments. The y-axis represents the Wasserstein distance between the evolutionary fingerprints of the alignments. Contours from a bivariate normal kernel density is superimposed on the plot to clarify the distribution of points in the denser regions. (PNG) [file ppat.1014457.s003.png]

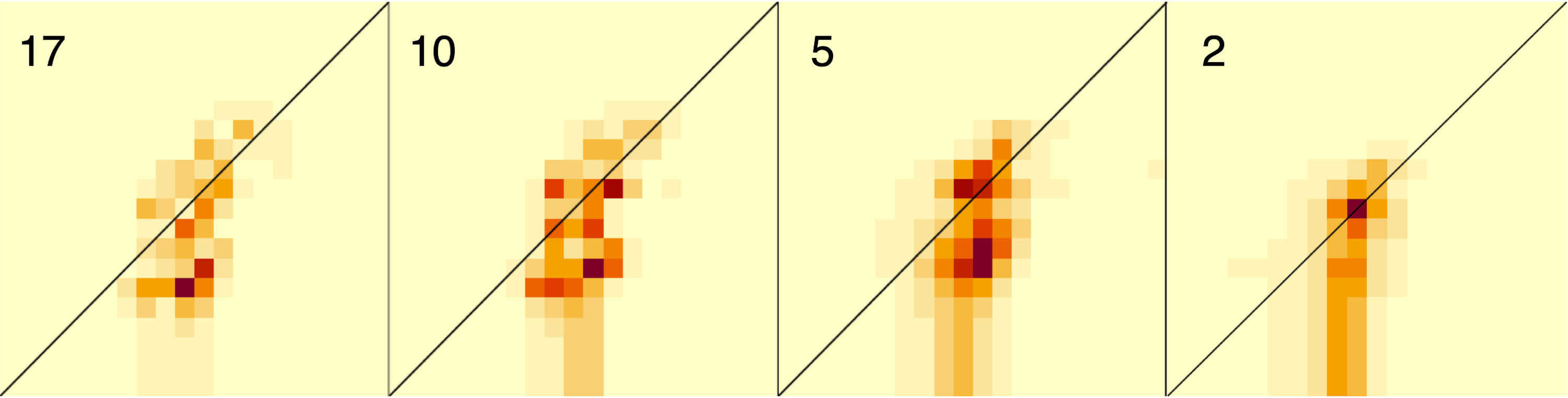

Supplement: S4 Fig — Each fingerprint depicts the posterior probability distribution over a fixed grid of 20×20 dS (x-axis) and dN (y-axis) values. Darker cell shades correspond to higher posterior probabilities. These fingerprints were derived from progressively smaller numbers of HIV-1 env sequences, resulting in shorter tree lengths (as measured by the expected number of nucleotide substitutions, upper left). (PNG) [file ppat.1014457.s004.png]

Alignment length

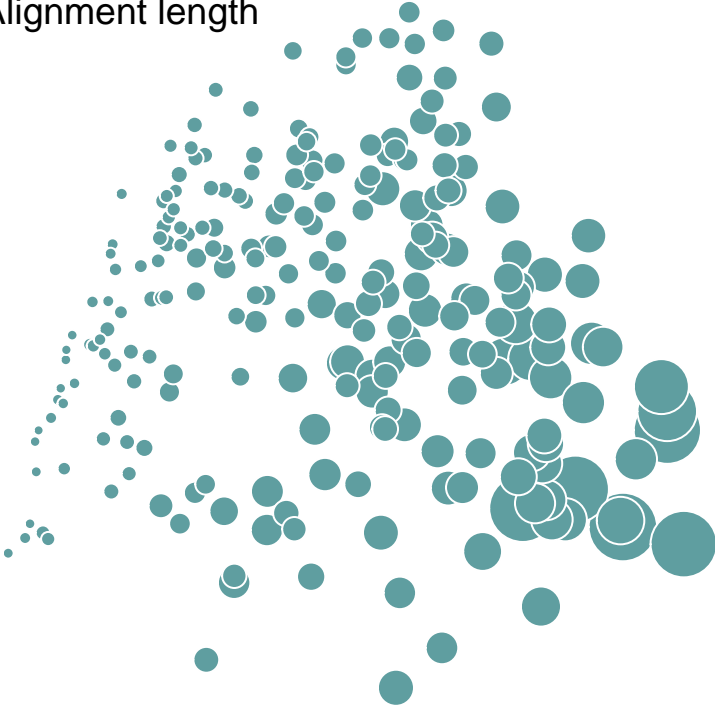

Tree length

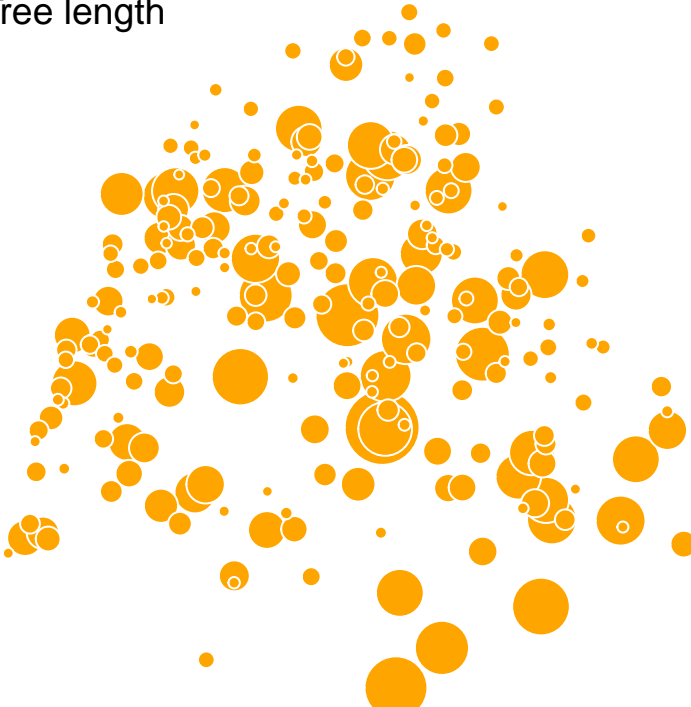

Supplement: S5 Fig — Both plots represent the same multidimensional scaling (MDS) projection of the Wasserstein distance matrix for the full gene alignments, i.e., without tree pruning. Point area is scaled in proportion to alignment length (left) or with an affine transformation (fixed minimum area) on tree length (right). (PDF) [file ppat.1014457.s005.pdf]

Alignment length

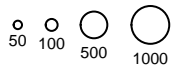

Residualized

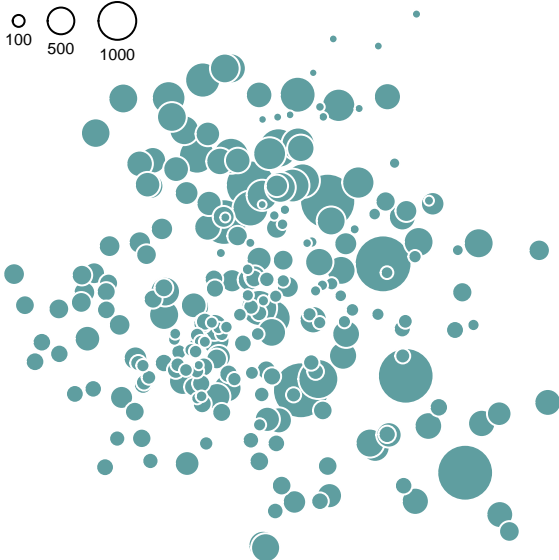

Tree length

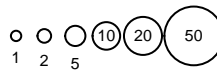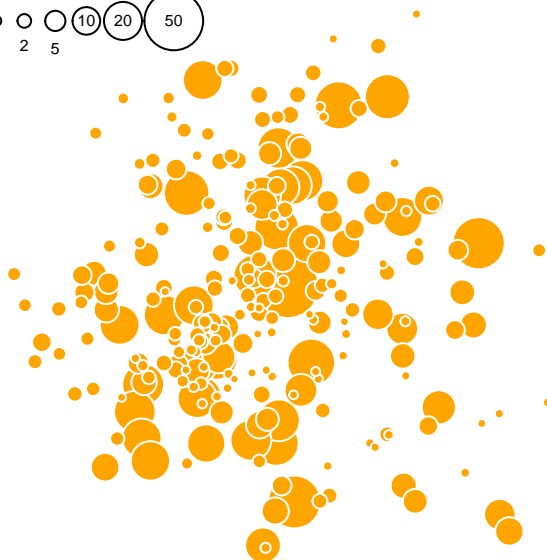

Downsampled

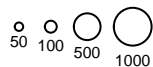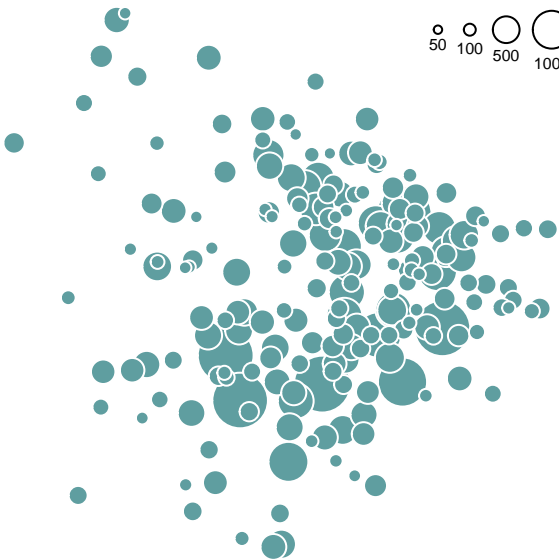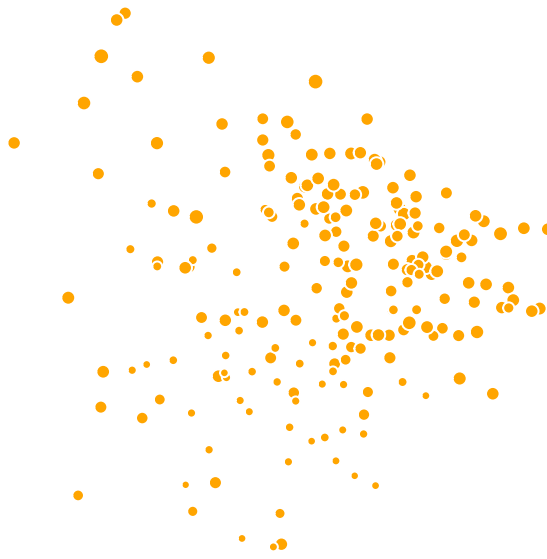

Supplement: S6 Fig — Each plot is derived from the same multidimensional scaling projection of the Wasserstein distance matrix for samples of 100 codon sites from gene alignments. Points (open circles, black) corresponding to the 10 replicate samples from a given gene alignment are highlighted for a random selection of viruses and protein-coding genes (labels). (PDF) [file ppat.1014457.s006.pdf]

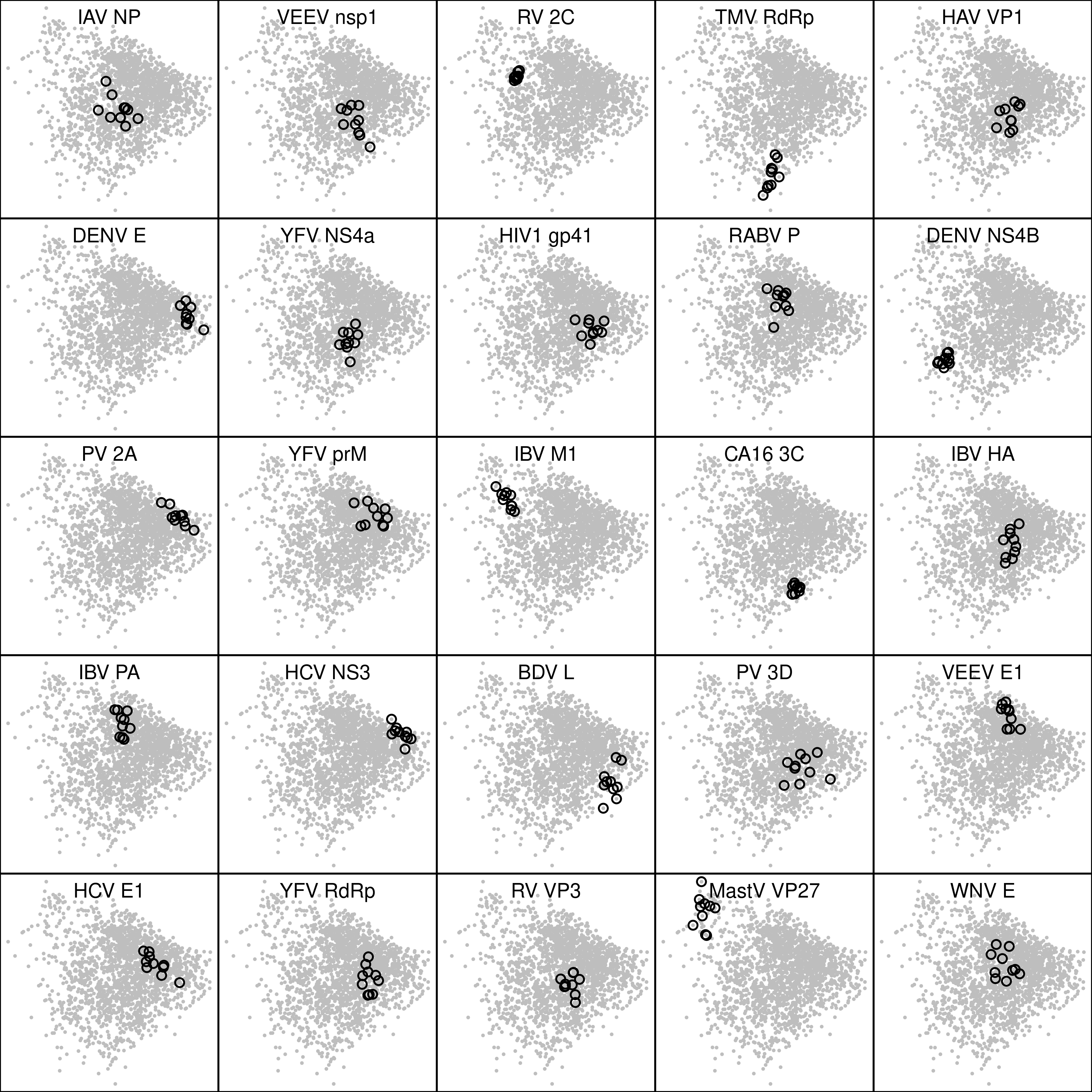

Supplement: S7 Fig — These plots depict MDS projections of the Wasserstein distance matrices in which the confounding effects of genetic variation were removed by one of two methods: (1) by regressing out the effects of alignment length and tree length (residualized, top row), or; (2) by progressively removing sequences associated with the longest terminal branches in the phylogeny, and sampling codon sites from the remaining sequences at random without replacement (bottom row). Each point represents a single gene alignment or the centroid of 10 random samples of 100 codon sites from each alignment; alignments fewer than 100 codon in length were excluded from the latter. Point area is scaled to the number of codon sites (alignment length) in the original alignment, or to tree length (as in S5 Fig). (PNG) [file ppat.1014457.s007.png]

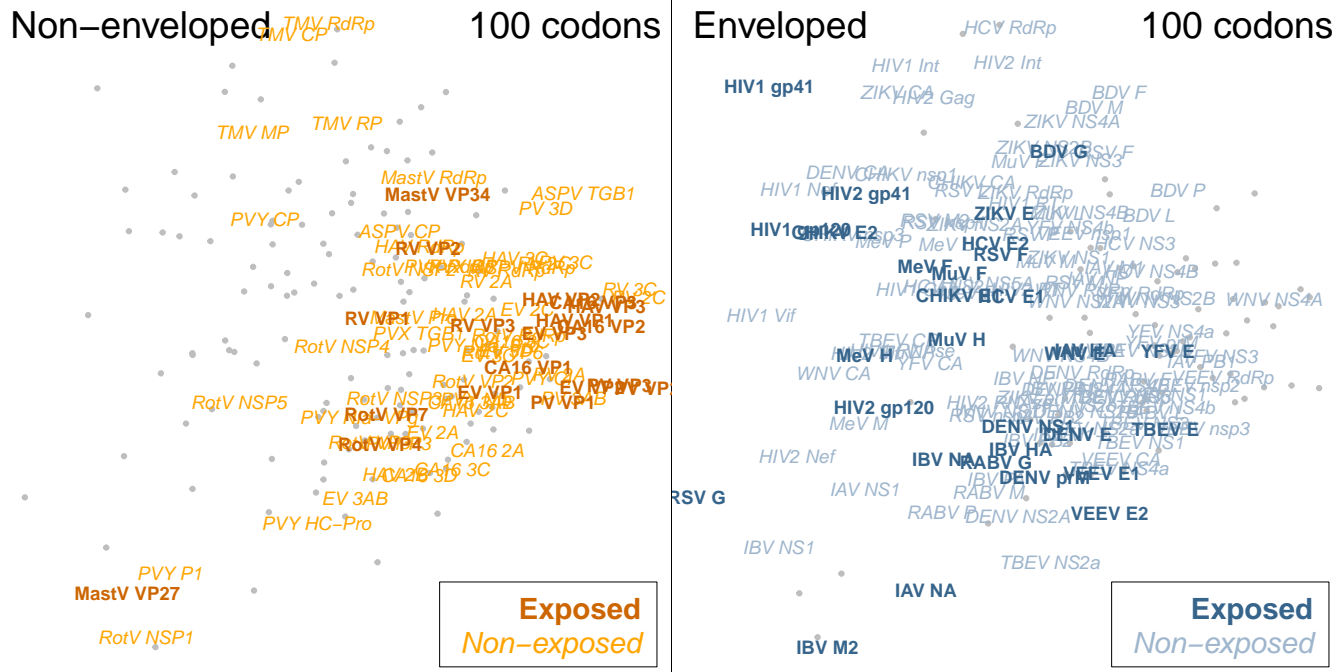

Supplement: S8 Fig — Each point represents the centroid of ten replicate samples of L = 100 codon sites from a gene alignment that was downsampled to normalize tree lengths. The x− and y-axes capture 51% and 15% of the variance, respectively. Replicate samples formed distinct clusters when visualizing the entire distance matrix (S6 Fig). Requiring a minimum of 100 codons excluded 41 (16.8%) out of 244 gene alignments from our analysis; the median alignment length was 255.5 (interquartile range, IQR: 130−466 codons; S1A Fig). Proteins from enveloped viruses are labeled on the left side, and non-enveloped viruses on the right. Each point is labeled with the respective virus and protein, and styled to indicate surface-exposed and non-exposed states as in Fig 4. Results from PERMANOVA are provided in Supporting Information (S2 Table). (PDF) [file ppat.1014457.s008.pdf]

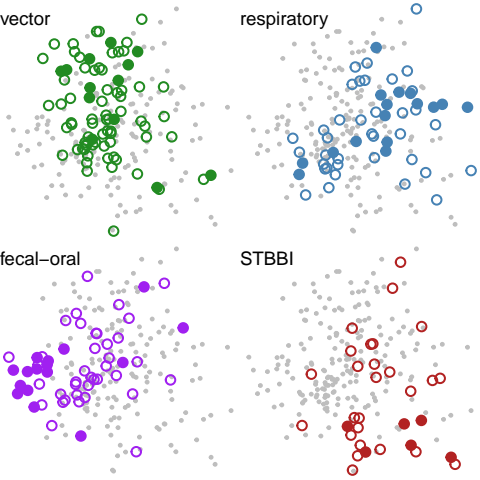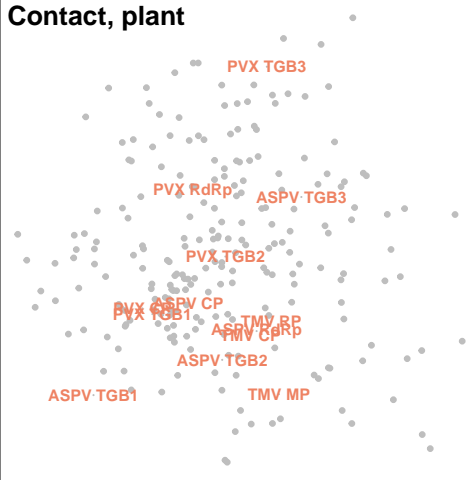

Supplement: S11 Fig — Each plot depicts the same MDS projection as Fig 4, except points are highlighted for proteins associated with viruses with different modes of transmission. The four plots on the left-hand side reproduce Fig 5, with filled circles for surface-exposed proteins and open circles otherwise. The remaining plot highlights the evolutionary fingerprints associated with proteins from plant viruses that are predominantly transmitted by direct contact, e.g., contaminated farm equipment or grafting. Fingerprints were not significantly different for this group versus all other data (PERMANOVA R2 < 0.01, P = 0.23). (PDF) [file ppat.1014457.s011.pdf]
