# Supplementary material for: Selection profiles in RNA viruses reflect the characteristics of viruses more than individual proteins
Source: PLoS Pathog. 2026 Jul 24;22(7):e1014457. doi: 10.1371/journal.ppat.1014457 (PMC13432152; doi:10.1371/journal.ppat.1014457)
Supplement: S9 Fig — Like S8 Fig, these plots depict the multidimensional scaling projection of the Wasserstein distance matrix for downsampled alignments. Each point represents the centroid of 10 random samples of 50 codon sites from each alignment, labeled with abbreviations for virus and gene product. Labels are highlighted in bold for surface-exposed proteins and italics for enveloped viruses. The distance matrices of centroids for L = 50 and L = 100 codons were significantly correlated (Mantel test, r = 0.96, P < 10-5). Results from PERMANOVA tests are provided in Supporting Information (S2 Table). (PDF) [file ppat.1014457.s009.pdf]

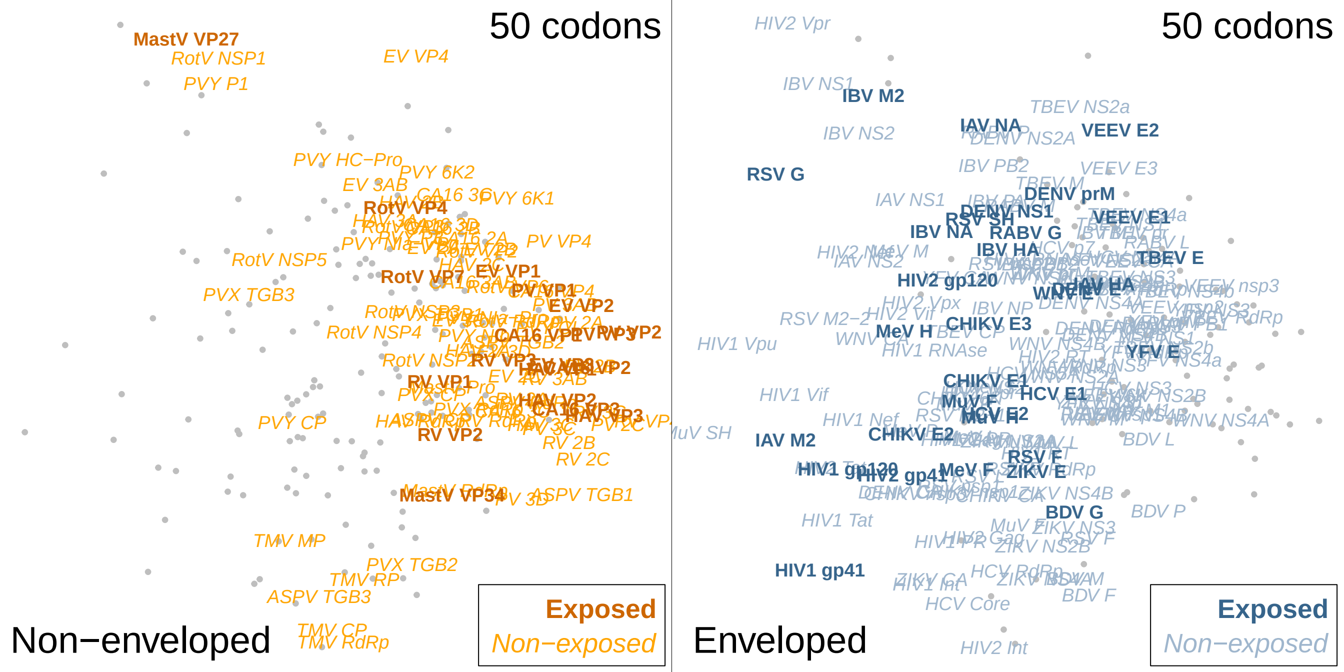

50 codons
Non−enveloped
Exposed
Non−exposed
MastV VP27
RotV NSP1
EV VP4
PVY P1
PVY HC−Pro
PVY 6K2
EV 3AB
PVY 6K1
RotV VP4
PV VP4
RotV NSP5
RotV VP7
EV VP1
PVX TGB3
PV VP1
EV VP2
RotV NSP4
PV VP2
RV VP1
PVY CP
RV VP2
RV 2B
RV 2C
MastV VP34
ASPV TGB1
TMV MP
PVX TGB2
TMV RP
ASPV TGB3
TMV CP
TMV RdRp
50 codons
Enveloped
Exposed
Non−exposed
HIV2 Vpr
IBV NS1
IBV M2
TBEV NS2a
IAV NA
VEEV E2
IBV NS2
DENV NS2A
RSV G
IBV PB2
VEEV E3
DENV prM
IAV NS1
DENV NS1
VEEV E1
RSV SH
IBV NA
RABV G
IBV HA
TBEV E
HIV2 gp120
VEEV nsp3
CHIKV E3
MeV H
RSV M2−2
HIV1 Vpu
YFV E
CHIKV E1
HCV E1
HIV1 Vif
MuV F
HCV E2
MuV H
HIV1 Nef
WNV NS4A
MuV SH
CHIKV E2
IAV M2
BDV L
RSV F
HIV1 gp120
HIV2 gp41
MeV F
ZIKV E
ZIKV NS4B
BDV G
BDV P
HIV1 Tat
ZIKV NS3
ZIKV NS2B
HIV1 gp41
HCV RdRp
BDV F
HCV Core
HIV2 Int
