# Supplementary material for: Selection profiles in RNA viruses reflect the characteristics of viruses more than individual proteins
Source: PLoS Pathog. 2026 Jul 24;22(7):e1014457. doi: 10.1371/journal.ppat.1014457 (PMC13432152; doi:10.1371/journal.ppat.1014457)
Supplement: S10 Fig — Each plot depicts the same MDS projection as Fig 4, except points are highlighted for proteins associated with viruses in one of the six families with multiple species in our data set. Points are filled for surface-exposed proteins, and open otherwise. (PDF) [file ppat.1014457.s010.pdf]

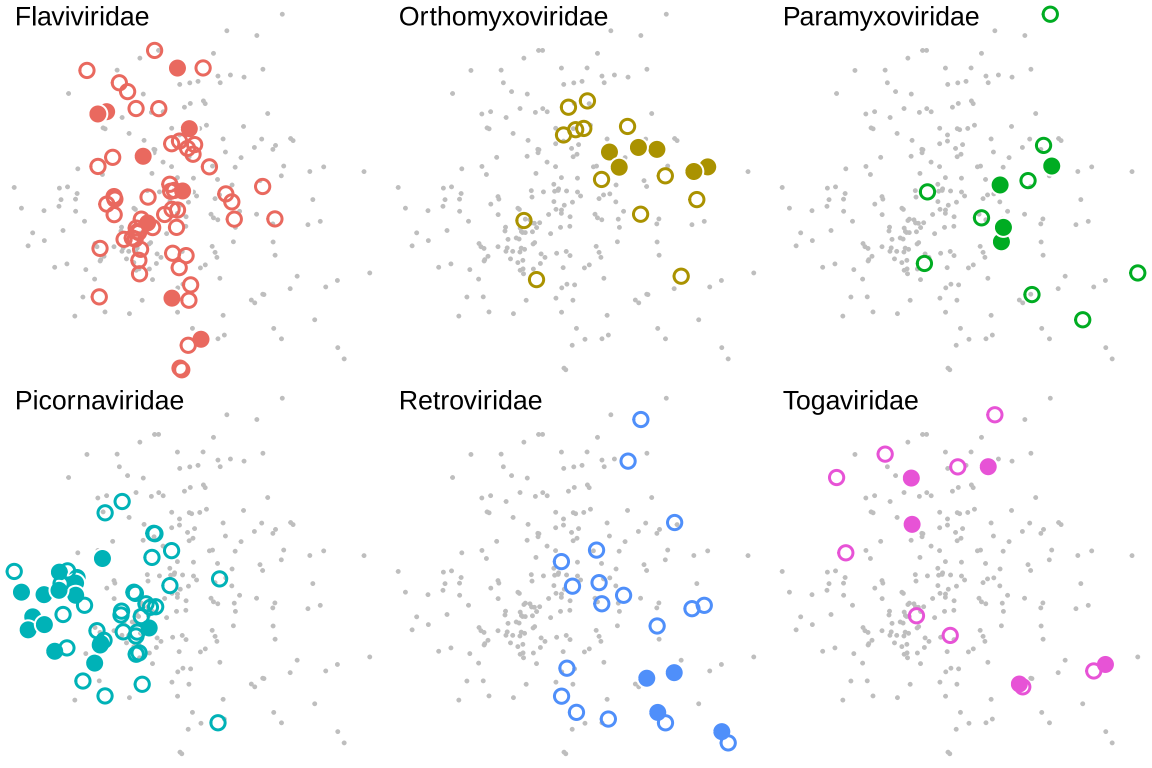

Flaviviridae
Orthomyxoviridae
Paramyxoviridae
Picornaviridae
Retroviridae
Togaviridae
