# Supplementary material for: Selection profiles in RNA viruses reflect the characteristics of viruses more than individual proteins
Source: PLoS Pathog. 2026 Jul 24;22(7):e1014457. doi: 10.1371/journal.ppat.1014457 (PMC13432152; doi:10.1371/journal.ppat.1014457)
Supplement: S1 Table — Virus = abbreviated virus name (see Table 1). Followed by the Genbank accession of the reference genome used for determining gene coordinates; if the genome is segmented, then the accessions are provided alongside the respective gene products (Protein). Abbrv = abbreviation of protein name for figures. Ex? = is the protein classified as surface-exposed? L = the number of codon sites prior to normalizing alignment lengths by random sampling. Coords = nucleotide coordinates in reference genome, determined by pairwise alignment of the consensus sequence of the curated data set. Multiple ranges are given for products of spliced exons, to remove indels with respect to the reference, or when an overlapping open reading frame was removed from the alignment. N = the number of sequences after normalizing tree length. TL = total tree length (expected substitutions per nucleotide site) after normalization by pruning. (PDF) [file ppat.1014457.s012.pdf]

S1 Table. Summary of viral protein names and characteristics.

Virus	Protein	Abbrv	Ex?	*L*	Coords	*N*	TL
PVX (NC_011620.1)	capsid protein	CP	No	237	5650-6360	236	1.86
	replication-associated protein	RdRp	No	1456	85-4452	227	1.96
	triple gene block 1	TGB1	No	220	4486-5145	236	1.94
	triple gene block 2	TGB2	No	88	5165-5425	236	1.65
	triple gene block 3	TGB3	No	49	5493-5636	235	1.38
MastV (PQ510858.1)	RdRp	RdRp	No	514	2793-4331	85	1.57
	serine-protease	Pro	No	956	85-2772	77	2.00
	capsid spike protein VP27	VP27	Yes	385	5599-6640, 6643-6687	54	2.79
	capsid core domain	VP34	Yes	394	4327-5508	54	1.49
ASPV (NC_003462.2)	coat protein	CP	No	396	7956-8018, 8073-9197	21	1.72
	RNA polymerase	RdRp	No	2180	60-6599	23	1.66
	triple gene block 1	TGB1	No	223	6711-7379	32	1.97
	triple gene block 2	TGB2	No	90	7384-7653	29	1.83
	triple gene block 3	TGB3	No	41	7745-7864	63	1.84
BDV (OR468838.1)	glycoprotein	G	Yes	475	2270-3691	92	0.84
	matrix protein	M	No	114	1840-2181	55	0.60
	nucleoprotein	F	No	370	1-1110	83	0.64
	phosphoprotein	P	No	130	1435-1821	67	0.65
	RNA dependent RNA polymerase	L	No	1692	3694-8766	90	0.90
DENV (NC_001474.2)	anchored capsid protein ancC	CA	No	114	97-438	341	1.62
	envelope protein	E	Yes	495	937-2421	733	1.75
	membrane glycoprotein precursor	prM	Yes	166	439-936	490	2.07
	nonstructural protein 1	NS1	Yes	352	2422-3477	671	1.85
	nonstructural protein 2A	NS2A	No	218	3478-4131	474	2.34
	nonstructural protein 2B	NS2B	No	130	4132-4521	424	2.06
	nonstructural protein 3	NS3	No	618	4522-6375	754	1.75
	nonstructural protein 4A	NS4A	No	127	6376-6756	239	1.59
	nonstructural protein 4B	NS4B	No	248	6826-7569	590	1.83
	RNA-dependent RNA polymerase	RdRp	No	901	7570-10272	823	1.83
HCV (NC_004102.1)	core protein	Core	No	30	825-914	196	3.04
	envelope glycoprotein 1	E1	Yes	192	915-1490	73	1.94
	envelope glycoprotein 2	E2	Yes	362	1494-2579	41	1.76
	non-structural protein 2	NS2	No	217	2769-3419	105	1.96
	non-structural protein 3	NS3	No	631	3420-5087, 5089-5312	74	1.84
	non-structural protein 4A	NS4A	No	54	5313-5474	138	1.88
	non-structural protein 4B	NS4B	No	261	5475-5773, 5775-6070, 6072-6257	74	1.76
	non-structural protein 5A	NS5A	No	446	6264-7601	73	1.95
	non-structural protein 5B	RdRp	No	591	7602-9374	98	1.91

	transmembrane protein	p7	No	63	2580-2768	87	1.88
TBEV (NC_001672.1)	core protein	CP	No	111	136-468	238	1.99
	envelope protein	E	Yes	496	973-2460	219	1.93
	matrix protein	M	No	75	748-972	219	2.13
	non-structural protein 1	NS1	No	352	2461-3516	221	1.98
	non-structural protein 2a	NS2a	No	230	3517-4206	276	2.79
	non-structural protein 2b	NS2b	No	131	4207-4599	219	1.80
	non-structural protein 3	NS3	No	621	4600-6462	221	1.97
	non-structural protein 4a	NS4a	No	149	6463-6909	219	2.10
	non-structural protein 4b	NS4b	No	252	6910-7665	216	1.87
	non-structural protein 5	RdRp	No	903	7666-10374	218	1.93
	pre protein	pr	No	93	469-747	224	1.96
WNV (NC_009942.1)	anchored capsid protein	CA	No	123	97-465	237	0.93
	envelope protein	E	Yes	501	967-2469	790	1.33
	membrane protein	M	No	75	742-966	136	0.98
	nonstructural protein 1	NS1	No	352	2470-3525	627	1.35
	nonstructural protein 2A	NS2A	No	229	3526-3586, 3590-3995, 3999-4218	510	1.58
	nonstructural protein 2B	NS2B	No	131	4219-4611	258	1.13
	nonstructural protein 3	NS3	No	619	4612-6468	685	1.22
	nonstructural protein 4A	NS4A	No	122	6469-6834	251	1.23
	nonstructural protein 4B	NS4B	No	255	6916-7680	506	1.49
	nonstructural protein 5	RdRp	No	905	7681-10395	842	1.21
	pre-membrane protein	pr	No	92	466-741	172	1.02
YFV (NC_002031.1)	anchored core protein	CA	No	121	6887-7636	60	1.25
	envelope protein	E	Yes	493	6440-6817	207	1.46
	M protein precursor	prM	No	164	4571-6439	107	1.49
	non-structural protein 1	NS1	No	352	4181-4570	153	1.33
	non-structural protein 2a	NS2a	No	224	3509-4180	146	1.63
	non-structural protein 2b	NS2b	No	130	2453-3508	96	1.37
	non-structural protein 3	NS3	No	623	974-2452	199	1.40
	non-structural protein 4a	NS4a	No	126	119-481	74	1.17
	non-structural protein 4b	NS4b	No	250	7637-10351	114	0.85
	RNA-dependent RNA polymerase	RdRp	No	905	482-973	95	1.06
ZIKV (NC_035889.1)	anchored capsid protein C	CA	No	122	108-473	76	0.57
	envelope protein	E	Yes	504	978-2489	254	0.79
	membrane glycoprotein precursor M	prM	No	168	474-977	135	0.95
	nonstructural protein 1	NS1	No	352	2490-3545	213	1.02
	nonstructural protein 2A	NS2A	No	226	3546-4223	160	0.81
	nonstructural protein 2B	NS2B	No	130	4224-4613	99	0.63
	nonstructural protein 3	NS3	No	617	4614-6464	234	0.69
	nonstructural protein 4A	NS4A	No	127	6465-6845	87	0.67
	nonstructural protein 4B	NS4B	No	251	6915-7667	158	0.74

	RNA-dependent RNA polymerase	RdRp	No	903	7668-10376	277	0.72
IAV	hemagglutinin (NC_004908.1)	HA	Yes	628	32-1711	271	2.02
	matrix protein 1 (NC_004907.1)	M1	No	234	33-734	364	1.98
	matrix protein 2 (NC_004907.1)	M2	Yes	82	33-58, 792-1011	378	1.81
	neuraminidase (NC_004909.1)	NA	Yes	510	1-1401	208	2.19
	nucleoprotein (NC_004905.2)	NP	No	511	40-1533	274	2.00
	nonstructural protein 1 (NC_004906.1)	NS1	No	167	27-527	336	2.22
	nonstructural protein 2 (NC_004906.1)	NS2	No	58	27-56, 721-861	323	2.05
	polymerase acidic protein (NC_004912.1)	PA	No	654	21-594, 781-2168	293	2.23
	polymerase basic protein 1 (NC_004911.1)	PB1	No	666	24-117, 391-2294	104	2.12
	polymerase basic protein 2 (NC_004910.1)	PB2	No	745	28-1894, 1937-2304	278	2.00
IBV	hemagglutinin (NC_002207.1)	HA	Yes	584	34-1785	1538	1.98
	matrix protein 1 (NC_002210.1)	M1	No	248	25-768	922	1.94
	matrix protein 2 (NC_002210.1)	M2	Yes	109	771-1097	442	1.86
	neuraminidase (NC_002209.1)	NA	Yes	368	351-1451	1203	1.66
	nucleoprotein (NC_002208.1)	NP	No	560	58-1737	1634	2.03
	nonstructural protein 1 (NC_002211.1)	NS1	No	229	43-729	1106	2.37
	nonstructural protein 2 (NC_002211.1)	NS2	No	70	55-75, 890-1063	511	2.12
	polymerase acidic protein (NC_002206.1)	PA	No	726	1-2178	1945	2.20
	polymerase basic protein 1 (NC_002204.1)	PB1	No	752	21-2276	1955	2.02
	polymerase basic protein 2 (NC_002205.1)	PB2	No	770	1-2310	1994	2.01
MeV (NC_001498.1)	fusion protein	F	Yes	550	5458-7109	229	0.78
	hemagglutinin protein	H	Yes	617	7271-9121	269	0.88
	large polymerase protein	L	No	2183	9234-15782	394	0.81
	matrix protein	M	No	335	3438-4442	197	0.98
	nucleocapsid protein	F	No	525	108-1682	250	0.88
	phosphoprotein	P	No	250	1807-1828, 2390-2490, 2701-3327	264	0.83
MuV (NC_002200.1)	fusion protein	F	Yes	538	4546-6159	180	0.84
	hemagglutinin	H	Yes	582	6614-8359	184	0.86
	large polymerase protein	L	No	2261	8438-15220	329	0.72
	matrix protein	M	No	375	3264-4388	150	0.73
	nucleocapsid protein	F	No	549	146-1792	169	0.80

	phosphoprotein	P	No	383	1979-2443, 2652-3149	149	0.87
	small hydrophobic protein	SH	No	57	6268-6438	38	0.69
CA16 (NC_001612.1)	nonstructural protein 2A	2A	No	150	3337-3786	231	1.97
	nonstructural protein 2B	2B	No	99	3787-4083	231	1.79
	nonstructural protein 2C	3C	No	329	4084-5070	251	2.09
	nonstructural protein 3AB	3AB	No	108	5071-5394	199	1.94
	nonstructural protein 3C	3C	No	183	5395-5943	228	2.03
	nonstructural protein 3D	3D	No	462	5944-7329	229	2.08
	structural protein 1	VP1	Yes	297	1719-1727, 2455-3336	233	1.99
	structural protein 2	VP2	Yes	254	958-1719	230	2.01
	structural protein 3	VP3	Yes	242	1720-2445	230	1.92
	structural protein 4	VP4	No	69	751-957	277	2.10
EV (NC_001612.1)	nonstructural protein 2A	2A	No	150	3337-3786	358	2.36
	nonstructural protein 2B	2B	No	99	3787-4083	435	2.28
	nonstructural protein 2C	2C	No	329	4084-5070	256	1.59
	nonstructural protein 3AB	3AB	No	108	5071-5394	221	1.60
	nonstructural protein 3C	3C	No	183	5395-5943	285	1.81
	nonstructural protein 3D	3D	No	462	5944-7329	343	2.02
	structural protein 1	VP1	Yes	297	2446-2471, 2473-3309, 3324-3336	450	2.11
	structural protein 2	VP2	Yes	254	958-1719	363	2.44
	structural protein 3	VP3	Yes	242	1720-2445	326	1.97
	structural protein 4	VP4	No	49	814-957	608	2.69
HAV (NC_001489.1)	structural protein 1B	VP2	Yes	222	804-1469	308	1.87
	structural protein 1C	VP3	Yes	246	1470-2207	333	2.06
	structural protein 1D	VP1	Yes	300	2208-3107	307	2.02
	nonstructural protein 2A	2A	No	183	3108-3656	305	1.66
	nonstructural protein 2B	2B	No	105	3675-3989	306	1.93
	nonstructural protein 2C	2C	No	335	3996-5000	331	2.25
	nonstructural protein 3A	3A	No	74	120	5001-5222	1.94
	nonstructural protein 3C	3C	No	215	306	5292-5936	1.43
	nonstructural protein 3D	RdRp	No	483	5949-7397	303	1.56
PV (NC_002058.3)	nonstructural protein 2A	2A	No	149	3386-3832	42	1.96
	nonstructural protein 2B	2B	No	97	3833-4123	36	1.95
	nonstructural protein 2C	2C	No	329	4124-5110	47	2.08
	nonstructural protein 3AB	3AB	No	109	5111-5437	41	2.08
	nonstructural protein 3C	3C	No	183	5438-5986	49	2.28
	nonstructural protein 3D	3D	No	461	5987-7369	66	2.16
	structural protein 1	VP1	Yes	302	2480-3385	71	2.00
	structural protein 2	VP2	Yes	272	950-1765	83	1.99
	structural protein 3	VP3	Yes	238	1766-2479	116	2.01
	structural protein 4	VP4	No	56	785-949	77	2.03
RV (NC_038311.1)	structural protein 1A	VP4	No	69	627-833	115	1.92
	structural protein 1B	VP2	Yes	264	834-1622	83	1.55
	structural protein 1C	VP3	Yes	238	1623-2336	83	1.54

	structural protein 1D	VP1	Yes	286	2337-2592, 2605-3203	83	1.59
	nonstructural protein 2A	2A	No	142	3198-3623	106	2.36
	nonstructural protein 2B	2B	No	95	3624-3908	85	1.63
	nonstructural protein 2C	2C	No	322	3909-4874	85	1.80
	nonstructural protein 3AB	3AB	No	98	4875-5168	56	1.80
	nonstructural protein 3C	3C	No	183	5169-5717	92	1.96
	nonstructural protein 3D	RdRp	No	460	5718-7097	97	2.06
RSV (NC_001803.1)	attachment protein	G	Yes	321	3233-4000	209	1.94
	fusion protein	F	Yes	574	4659-5506	339	0.87
	large protein	L	No	2165	5632-7353	492	0.73
	matrix protein	M	No	256	8468-14962	222	0.97
	matrix protein 2-1	M2-1	No	186	7576-8133	183	0.73
	matrix protein 2-2	M2-2	No	80	8162-8398	115	0.91
	nonstructural protein 1	nsp1	No	139	99-515	133	0.62
	nonstructural protein 2	nsp2	No	124	628-999	174	1.20
	nucleoprotein	F	No	391	1140-2312	257	0.76
	phosphoprotein	P	No	241	2348-3070	226	0.87
	small hydrophobic protein	SH	Yes	64	4274-4465	96	0.95
PVY (NC_001616.1)	6kD peptide 1	6K1	No	51	3503-3655	145	2.40
	6kD peptide 2	6K2	No	52	5558-5713	139	2.46
	cylindrical inclusion protein	CI	No	634	3656-5557	139	2.28
	coat protein	CP	No	267	8573-9373	411	1.98
	helper component proteinase	HC-Pro	No	456	1037-2404	639	1.71
	nuclear inclusion *a* proteinase	NIa-Pro	No	244	6278-7009	58	1.85
	nuclear inclusion *a* genome-linked protein	NIa-VPg	No	188	5714-6277	59	1.96
	nuclear inclusion *b* protein	NIb	No	521	7010-7144, 7151-7321, 7323-8578	96	2.02
	proteinase	P1	No	284	185-1036	532	2.01
	third protein	P3	No	291	2405-2913, 3136-3499	126	2.04
HIV1 (DQ676872.1)	group-specific antigen	Gag	No	429	1-1149, 1153-1290	51	1.73
	surface glycoprotein	gp120	Yes	421	6996-7615, 7913-8051	35	2.03
	transmembrane glycoprotein	gp41	Yes	253	5526-5831, 5925-6013, 6029-6623, 6699-6828, 6856-6995	35	1.36
	integrase	Int	No	270	8056-8117, 8130-8670	54	1.20
	negative effector	Nef	No	225	1500-1757	34	1.76
	protease	PR	No	87	3078-3437	54	1.17
	RNAse	RNAse	No	120	1758-3077	54	1.69
	reverse transcriptase	RT	No	440	3438-4247	54	1.63
	transactivator of transcription	Tat	No	40	5059-5175	45	1.79

	viral infectivity factor	Vif	No	155	4303-4764	56	2.11
	viral protein R	Vpr	No	70	4830-5036	64	2.07
	viral protein U	Vpu	No	52	5285-5440	32	1.93
HIV2 (NC_001722.1)	group-specific antigen	Gag	No	430	1103-2392	50	1.81
	surface glycoprotein	gp120	Yes	465	9288-9888	25	1.96
	transmembrane glycoprotein	gp41	Yes	215	6704-6767, 6771-7042, 7130-7279, 7301-8071, 8093-8233	25	1.41
	integrase	Int	No	270	8234-8860, 9104-9121	50	1.18
	negative effector protein	Nef	No	201	2671-2934	20	1.82
	protease	PR	No	89	2935-4251	50	1.45
	RNAse	RNAse	No	120	4612-5421	50	1.60
	reverse transcriptase	RT	No	439	4252-4611	50	1.76
	transactivator of transcription	Tat	No	24	6558-6626	30	1.98
	viral infectivity factor	Vif	No	135	5495-5896	32	1.87
	viral protein R	Vpr	No	54	6239-6400	41	1.99
	viral protein X	Vpx	No	56	6072-6191, 6195-6202, 6204-6237	41	2.19
RABV (NC_001542.1)	glycoprotein	G	Yes	524	3318-4889	313	1.72
	matrix protein	M	No	202	2496-3101	289	1.86
	nucleoprotein	F	No	452	71-1420	345	2.12
	phosphoprotein	P	No	297	1514-2404	317	1.95
	polymerase	L	No	2127	5418-11798	490	2.18
RotV	nonstructural protein 1 (NC_011500.2)	NSP1	No	486	31-490, 494-785, 789-858, 874-1155, 1157-1272, 1279-1457, 1461-1506	227	1.97
	nonstructural protein 2 (NC_011502.2)	NSP2	No	317	47-997	91	1.85
	nonstructural protein 3 (NC_011501.2)	NSP3	No	310	35-964	74	1.52
	nonstructural protein 4 (NC_011504.2)	NSP4	No	175	42-566	55	1.48
	nonstructural protein 5 (NC_011505.2)	NSP5	No	106	22-79, 356-615	161	2.69
	virus protein 1 (NC_011507.2)	RdRp	No	1088	19-3282	90	2.06
	virus protein 2 (NC_011506.2)	VP2	No	862	17-50, 63-76, 125-2662	70	1.61
	virus protein 3 (NC_011508.2)	VP3	No	835	50-2554	97	2.00
	virus protein 4 (NC_011510.2)	VP4	Yes	775	10-410, 414-2337	94	2.01

	virus protein 6 (NC_011509.2)	VP6	No	397	24-1214	100	1.90
	virus protein 7 (NC_011503.2)	VP7	Yes	326	49-1026	73	2.04
CHIKV (KY704002.1)	6K protein	6K	No	49	9772-9918	58	0.72
	capsid protein	CA	No	261	7528-8310	202	0.69
	envelope glycoprotein 1	E1	Yes	425	10000-11271, 298	0.84	
	envelope glycoprotein 2	E2	Yes	422	8503-9454, 9458-9510, 9514-9771	295	0.73
	envelope glycoprotein 3	E3	Yes	64	8311-8502	78	0.87
	mRNA-capping enzyme	nsp1	No	535	50-1654	338	0.73
	non-structural protein 3	nsp3	No	519	4049-5605	306	0.64
VEEV (NC_001449.1)	6kD protein	6K	No	46	9833-9970	33	1.65
	capsid protein	CA	No	281	7562-8392	95	1.98
	envelope glycoprotein 1	E1	Yes	427	10049-11326	109	1.90
	envelope glycoprotein 2	E2	Yes	423	8564-9832	189	1.94
	envelope glycoprotein 3	E3	No	55	8387-8551	40	1.99
	nonstructural protein 1	nsp1	No	535	45-1649	109	1.38
	nonstructural protein 2	nsp2	No	794	1650-4031	120	1.59
	nonstructural protein 3	nsp3	No	330	4032-5021	88	1.78
	nonstructural protein 4	RdRp	No	607	5700-7520	115	1.76
TMV (NC_001367.1)	coat protein	CP	No	159	5712-6188	52	0.64
	movement protein	MP	No	263	69-3416	63	0.76
	RdRp	RdRp	No	469	4921-5706	73	0.67
	replication protein	RP	No	1117	3495-4901	85	0.73
