# Supplementary material for: Selection profiles in RNA viruses reflect the characteristics of viruses more than individual proteins
Source: PLoS Pathog. 2026 Jul 24;22(7):e1014457. doi: 10.1371/journal.ppat.1014457 (PMC13432152; doi:10.1371/journal.ppat.1014457)
Supplement: S2 Table — Wasserstein distances were averaged between replicates for every pair of virus proteins to obtain a reduced distance matrix for the centroids. Tests were run for 10,000 permutations using the adonis2 function in the R package vegan. The term ‘exposed:enveloped’ represents the interaction between the respective factors. R2(%) represents the percentage of variation explained by each factor. Results are provided for Wasserstein distance matrices calculated for fingerprints on both log-offset and integer indexed coordinates on the grid of dS and dN rates. (PDF) [file ppat.1014457.s013.pdf]

S2 Table. PERMANOVA results for down-sampled datasets.

		log-offset		integer	
Codons	Term	R^2(%)	P	R^2(%)	P
50	exposed	0.32	0.42	0.31	0.44
	enveloped	10.8	$\leq 10^{-4}$	10.1	$\leq 10^{-4}$
	exposed:enveloped	1.36	0.0106	1.35	0.0093
50	family	16.5	$\leq 10^{-4}$	29.1	$\leq 10^{-4}$
100	exposed	0.55	0.245	0.50	0.281
	enveloped	9.17	$\leq 10^{-4}$	8.63	$\leq 10^{-4}$
	exposed:enveloped	2.55	0.0021	2.48	0.0011
100	family	32.5	$\leq 10^{-4}$	32.9	$\leq 10^{-4}$
