# Supplementary material for: Selection profiles in RNA viruses reflect the characteristics of viruses more than individual proteins
Source: PLoS Pathog. 2026 Jul 24;22(7):e1014457. doi: 10.1371/journal.ppat.1014457 (PMC13432152; doi:10.1371/journal.ppat.1014457)
Supplement: S3 Table — The following proteins were identified as viral suppressors of RNA silencing (VSRs) according to the literature: TGB1 for potato virus X [Aguilar et al. (2015) J Virol 89(4): 2090–2103], HC-Pro and NIa-VPg for potato virus Y [Cheng and Wang (2017) J Virol 91: e01478-16], RP for tobacco mosaic virus [Vogler et al. (2007) J Virol 81(19): 10379–10388], and CP for apple stem pitting virus [Ma et al. (2019) Virology J 16: 20]. Tests were run for 10,000 permutations on the residualized Wasserstein distance matrix using the adonis2 function in the R package vegan. The term ‘exposed:enveloped’ represents the interaction between the respective factors. R2(%) represents the percentage of variation explained by each factor. (PDF) [file ppat.1014457.s014.pdf]

S3 Table. PERMANOVA results for different treatments of proteins associated with plant viruses.

Treatment	Term	R^2(%)	P
No plant proteins exposed	exposed	0.51	0.029
	enveloped	3.18	$\leq 10^{-4}$
	exposed:enveloped	1.23	0.0011
Coat proteins are exposed	exposed	2.50	0.043
	enveloped	3.19	$\leq 10^{-4}$
	exposed:enveloped	0.94	0.0026
VSRs are exposed	exposed	0.53	0.028
	enveloped	3.19	$\leq 10^{-4}$
	exposed:enveloped	0.90	0.0042
Both coat and VSRs are exposed	exposed	0.49	0.035
	enveloped	3.22	$\leq 10^{-4}$
	exposed:enveloped	0.74	0.0092
No plant viruses	exposed	0.53	0.029
	enveloped	3.54	$\leq 10^{-4}$
	exposed:enveloped	1.15	0.0015
