# Supplementary material for: Selection profiles in RNA viruses reflect the characteristics of viruses more than individual proteins
Source: PLoS Pathog. 2026 Jul 24;22(7):e1014457. doi: 10.1371/journal.ppat.1014457 (PMC13432152; doi:10.1371/journal.ppat.1014457)
Supplement: Attachment — Submitted filename: response-letter.pdf [file ppat.1014457.s015.pdf]

Schulich
MEDICINE & DENTISTRY

Pathology and
Laboratory Medicine

May 7, 2026

Dear colleagues,

Thank you for considering our manuscript for PLOS Pathogens. We appreciate the time that the editors and reviewers have volunteered to critically evaluate our work. Please see below itemized lists of the comments that we received from the editor and reviewers (quoted and italicized), accompanied by our response to each comment, including a description of the specific revisions to the manuscript.

Comments from Editor

1. *"I thank the authors for submitting their manuscript to Plos Pathogens. Three reviewers have now shared their evaluations, and all three commended the manuscript. The reviewers particularly appreciated the importance of the question and the care taken to curate the data, including the care taken to work with good alignments. This is the basis of the kind of analysis conducted by the authors, yet it is too often neglected."*

   Thank you for this supportive feedback. Curation of the data turned out to be the most time-consuming and challenging part of the study.

2. *"The reviewers (1 and 2 especially) make extensive recommendations to improve the statistical approaches used, and raise concerns that I share about a number of statistical limitations. First, reviewers 1 and 3 point out the confusion between negative and positive selection, and reviewer 1 suggests the solution of using the RELAX test of relaxation. This is important since differences in positive selection could be masked when they occurred on top of different backgrounds of negative selection. I agree with the reviewer that it would clarify the conclusions of the manuscript."*

   Thank you for raising this point. We acknowledge that it was incorrect to characterize the observed difference between mean fingerprints as 'relaxed purifying selection'. We have removed this statement from the manuscript. The problem with using the RELAX method in HyPhy is that it requires the user to partition the tree into 'foreground' and 'background' sets of branches. In other words, this test is designed to look for a change in selection at an internal branch in the tree, which is propagated to its descendants. Unfortunately, we cannot apply this method to the present study because we are measuring the selective regimen over the entire gene tree for a virus protein, and comparing that regimen to other proteins and viruses.

   Reviewer 1 also suggested an alternative to the RELAX method, which is to perform a formal distributional test on the evolutionary fingerprints obtained for surface-exposed proteins from enveloped and non-enveloped viruses. We propose that the PERMANOVA method recommended by Reviewer 2 is a more direct test of the hypothesis that these groups tend to have different fingerprints. In addition, we are retracting the claim that the difference can be attributed to relaxed purifying selection, as it is not a core result of the study.

3. *"Second, and I believe that this is an extremely important point raised by reviewer 1, the sub-sampling used could have indeed killed the statistical power to discern differences between classes of proteins. I agree with reviewer 1, and it is also my own experience that alignments of less than 100 codons typically result in very high variance, hence low discriminatory power. The authors should explore the suggestion to add the confounder of gene length as a covariate."*

   We concur that randomly down-sampling alignments would have reduced our ability to resolve detailed patterns of selection. However, we previously demonstrated that the variance induced by down-sampling did not obscure the underlying signal — for instance, the replicate samples formed tight clusters in the MDS projection for each virus protein (Figure S5 in original version). Nevertheless, we have followed the procedure suggested by Reviewer 1 by regressing the MDS coordinates on the covariates of alignment length and tree length, and reconstructing a new distance matrix from the residuals. We found that the resulting distance matrix was significantly correlated with the distance matrices in the original manuscript, and the subsequent analytical results were qualitatively unchanged. Because this 'residualization' approach recommended by Reviewer 1 allowed us to analyze more gene alignments than our down-sampling approach, we decided to present these new results in the main text of the revised manuscript, and we have moved our previous results from down-sampling to Supplementary Material.

4. *"Reviewers 1 and 2 make a number of other important recommendations about the statistical choices made. In their revision, the authors can implement these recommendations. If they succeed, the authors can modify their manuscript accordingly. If they fail, the authors can discuss that they tried them, and explain why they think they failed (limitations of the data, etc.). If the reviewers missed something that constitutively prevents the authors from being able to enact specific recommendations, the authors can add a detailed explanation to the discussion, or wherever appropriate in the revised manuscript. I look forward to receiving the revision."*

   We thank the reviewers for providing these insightful recommendations. We have implemented these recommendations in our revisions, including the replacement of k-nearest neighbours with PERMANOVA to test for associations between pairwise distances and groups, and testing for potential phylogenetic confounding at the level of virus families. In other cases, the reviewer's recommendations stemmed from a lack of clarity in our original manuscript, and we have revised the text accordingly.

Reviewer 1

1. *" The authors make a specific, implicitly strong claim in the abstract: 'we show that this pattern is more consistent with relaxed purifying selection than adaptive evolution in proteins associated with viral envelopes'. However, the only arguments to this effect appeal to visual inspections of density plots and heatmaps, and are not supported by formal statistical tests. The claim in the abstract overstates the extent of analytical support for this attribution. Specific, formal tests for the relaxation of selection exist (e.g., the RELAX model in the HyPhy suite), or the authors must provide formal distributional tests on the dN/dS fingerprints to support this claim."*

   (Note this point was raised again under Minor Issues.)

   We apologize for this. The reviewer is correct that the phrase 'relaxed purifying selection' has a specific meaning in molecular evolution, in that there is a shift in the selective regime over time such that some sites are no longer under significant purifying selection. We were trying

to describe the observed difference between mean fingerprints for surface-exposed proteins from enveloped and non-enveloped viruses. Specifically, we observed higher posterior probabilities at $dN < dS$ grid points for non-enveloped viruses. There was no statistical test of this observation, however. Moreover, the observed difference does not directly support a hypothesis of relaxed purifying selection. We have removed this claim from the revised manuscript. In addition, we have removed the associated supplementary figure, as these results have been superseded by the revised analysis.

2. *"The authors (correctly) identified that dN/dS estimates have dataset-specific variance (heteroscedasticity). An estimate derived from a short and low-diversity alignment is highly uncertain, while an estimate from a long alignment derived over a larger tree is much more precise (in general). However, by randomly subsampling alignments down to 50 or 100 codons and aggressively pruning the terminal branches of phylogenetic trees, this approach equalized the variance by maximizing the noise across all datasets, effectively destroying the evolutionary signal that was the object of detection. I really struggle to understand the logic here. Averaging over high-variance estimates is not going to properly reduce the variance of the resulting estimate; it is going to reduce its resolution (make it 'flatter'). Differential resolution is a feature that depends on the properties of the alignment, not a bug. If the concern is that clustering will reflect something about the length or the divergence of a gene, these covariates should be included directly in the statistical model rather than physically degrading the data."*

   We thank the reviewer for raising this issue and proposing a solution. Following their recommendation, we have repeated our analysis by regressing out the effects of these covariates (alignment length and tree length) from the MDS coordinates for the Wasserstein distance matrix, and then regenerated a new distance matrix from the residuals. Next, we ran permutation tests on the distribution of labels with respect to this 'residualized' distance matrix, and visualized these distances using a new MDS projection. The residualized distance matrix was significantly correlated with the distance matrix obtained from down-sampled alignments (Mantel test, $P < 10^{-4}$). Moreover, the clustering results were qualitatively the same as what we obtained from the down-sampling analysis. A major advantage of the residualization method is that we can retain all gene alignments, instead of discarding those that fall below thresholds for alignment and tree lengths. Thus, our revised manuscript now presents results from the residualization method in the main text, and results from the downsampling method have been moved to supplementary materials.

3. *"A technical question regarding the Figure S1 simulation: If you simulate under INDELible (which uses a slightly different substitution model, e.g., GY94 with possibly different equilibrium frequencies) and infer with FUBAR, what exactly are you comparing via RMSE? FUBAR was not really designed to estimate true point values of dN and dS at a site super-accurately; it is meant to estimate the posterior probability of a site belonging to a specific rate class on a grid. A much more sensible approach would be to calculate the Wasserstein distance between the simulated distribution of dN/dS and the inferred FUBAR grid density."*

   Please note that this issue is somewhat deprecated because our previous results from down-sampling alignments have been replaced in the main text with a new set of results based on the residualization method (see previous comment).

   We were initially surprised by the reviewer's statement that FUBAR is not designed to accurately estimate site-specific dN and dS rates. It is presented as one of the methods for measuring site-specific selection in HyPhy version 2.5 (Pond *et al.* 2020 MBE), and remains the recom-

mended option on the Datamonkey webserver for detecting pervasive (non-episodic) site-specific selection. However, we inquired with one of the developers of the method and learned that the reviewer is indeed correct — it was designed more for testing whether $dN/dS > 1$ at a site than for accurately estimating the specific dN and dS rates. We thank the reviewer for this astute observation.

In light of this, we repeated our analysis using FEL; these results are presented alongside the results using FUBAR. Given the sensitivity of evolutionary fingerprints to tree lengths and the updating of our results with a new approach, we did not calculate Wasserstein distances between simulations and FUBAR grid estimates.

4. *"Phylogenetic Confounding and Data Leakage. The supervised learning analysis (k-Nearest Neighbors) claims to distinguish surface proteins of enveloped versus non-enveloped viruses, but it is heavily confounded by viral taxonomy. The 'non-enveloped' dataset is overwhelmingly dominated by a single viral family (Picornaviridae), with every other virus coming from its own family. The 'enveloped' group is similarly dominated by Flaviviridae. By using standard Leave-One-Out Cross-Validation (LOOCV), the authors introduce significant possible data 'leakage'. When predicting the envelope status of a Dengue virus protein, the model can draw upon the relatively closely related Zika and West Nile proteins in the training set. The classifier could simply be capturing family-specific phylogenetic baseline traits rather than a generalizable 'envelope' signature. A strict Leave-One-Family-Out cross-validation would be much more robust."*

   Thank you for raising this issue. The reviewer is correct that the relationship between surface-exposure and evolutionary fingerprints is confounded by viral taxonomy. A similar point was also raised by Reviewer #2. These comments prompted us to revisit the distribution of fingerprints with respect to virus families. We discovered that the evolutionary fingerprints from the same virus family were significantly clustered with respect to the residualized distances (PERMANOVA, $P < 10^{-4}$). This observation motivated us to test whether there was an association between modes of transmission and fingerprints. Indeed, fingerprints for groups of viruses with different modes of transmission are significantly different. We acknowledge that interpreting these results is difficult because transmission modes are confounded with taxonomic groupings, and discuss this issue in detail in the revised manuscript. However, there are notable cases where fingerprints are more consistent with transmission groups than taxonomy, particularly for proteins associated with hepatitis C virus. In the Discussion section, we also expand on the issue of phylogenetic confounding.

5. *"Calculating the Wasserstein distance using a linear Euclidean norm on a grid of dN/dS values misrepresents biological reality. The dN/dS scale is non-linear: the evolutionary "work" required to shift a site's rate from 0.0 (perfect conservation) to 0.1 (strong conservation) is vastly different from shifting it from 5.1 to 5.2 (very strong diversification to very strong diversification). A linear cost function likely overweights noisy variance in the high-rate tails."*

   We apologize for this misunderstanding. The mass distributions for calculating the Wasserstein distance were calculated on the integer indices of the grid, and not the dN or dS values associated with those grid points. As a result, the amount of work required to move a mass from 0.0 to 2.0×10^{-5} was equivalent to moving the same mass from 3.8 to 5.0. In contrast, moving from 0.0 to 0.1 requires far more work, as this crosses roughly six grid points. We have clarified this in the Methods section with additional text and a mathematical formula that makes this use of grid indices more explicit.

6. *“The binomial regression (Figure 1B) is severely confounded by the statistical power of the selection inference method. The authors regress the log-odds of diversifying selection against the proportion of purifying sites. Both n+ and n- are counts of statistically significant sites, not true underlying biological states. Reaching significance is highly dependent on alignment-wide genetic variation (ie tree length). The observed negative correlation could very well be an artifact of the inference method losing statistical power (since negative selection is actually easier to detect for a given amount of sequence variation than positive selection).”*

   We agree that these regression analyses may have been hampered by our statistical power in detecting statistically significant sites. Our revised analysis removes the restriction on tree length and the size of the alignment. As a result, the number of sites under significant positive selection ($n+$) were substantially increased, and the negative correlation with the number of sites under negative selection ($n-$) disappeared, which confirms the reviewer's conjecture. This figure and accompanying text in our manuscript have both been updated with the new analysis.

7. *“The outliers discussed in the text (Rotavirus VP4/VP7 and Astrovirus VP27) are non-enveloped but were ”misclassified” as Enveloped. Picornaviridae (which make up the bulk of the non-enveloped set) have rigid ”canyon” capsids. Rotavirus and Astrovirus (the ”misclassified” non-enveloped viruses) have protruding spikes, structurally similar to enveloped glycoproteins. This suggests a highly plausible biological explanation: the clustering is not driven by the presence of a lipid ”Envelope,” but rather by ”Structural Protrusion / Spike Architecture.””*

   (Note this point was raised again under Minor Issues.)

   We agree completely with the reviewer. Our intent was to raise exactly this point in the Discussion section, *i.e.*, that rotavirus VP4 and mamastrovirus VP27 are both spike proteins (page 23, lines 6-17 in the original manuscript). Obviously, we did not convey this point adequately, and we apologize for this shortcoming. We have revised this section of the Discussion to make it clearer that the protrusion of these spike proteins is a likely explanation for the unusual placement of the respective evolutionary fingerprints, relative to other surface-exposed proteins from non-enveloped viruses.

8. *“The authors excluded plant viruses from the ‘surface-exposed’ category because ‘plants do not have an adaptive immune system.’ This is an oversimplification. Plants have robust RNA interference (RNAi) and R-gene mediated immunity that exert strong diversifying selection on viral coat proteins and viral suppressors of RNA silencing, even though the targets and mechanisms are distinct from vertebrate humoral immunity. They should be included or treated as a distinct, formal control group.”*

   (Note this point was raised again under Minor Issues.)

   We apologize for this oversimplification. In the revised manuscript, we have added some paragraphs to the Discussion section to provide a more accurate characterization of immunity in plants. Additionally, we repeated our analysis with five different treatments of proteins from plant viruses: (1) with no proteins from plant viruses labeled as exposed; (2) coat proteins labeled as surface-exposed; (3) with viral suppressors of RNA silencing (VSR) proteins as exposed; (4) with both coat and VSR proteins as exposed; and (5) with all proteins from plant viruses excluded (at the request of Reviewer 3). These modifications to the metadata had very little impact on the PERMANOVA test of differences between groupings of proteins with respect to surface exposure and enveloped status — these results are now included as a Supplementary Table.

Reviewer 2

1. *"Poor performance of a machine learning classifier does not feel like strong evidence of a true non-separation between two groups. As we understood the role of KNN in this paper, this was the primary approach to quantify the differences qualitatively examined in MDS space under the assumption that high performing classifiers corresponded to true differences between groups and poor classifiers corresponded to no true difference between groups. However, there are many factors that can make a classifier perform poorly and comparing a series of ML performance attributes between different models did not feel like a straightforward way to assess the authors' central question. For instance, the performance of leave one out cross validation with KNN framework is highly sensitive class imbalance (i.e., for comparing 'Surface vs non-exposed' and 'Polymerase vs. non-polymerase', Table 2). While the authors briefly mentioned that the high accuracy and low F1 metric is likely due to class imbalance, it would be helpful to evaluate and interpret the performance with concrete numbers of class size for each task. More importantly, there is no null hypothesis that can be straightforwardly rejected (see below to major comment 2), so we are left guessing at what a poor classifier actually means. When is a classifier 'good enough' that the authors would be convinced that there's true separation?"*

   We acknowledge that the performance of the k-nearest neighbour (kNN) classifier is not strong evidence of non-separation between groups. Following the reviewer's subsequent comment, we have replaced the kNN analysis with PERMANOVA tests of associations between pairwise distances and groups (see following point).

2. *"Relatedly, is there a reason that the authors did not use a formal hypothesis testing framework instead of fitting a ML model? It would be useful to report a version of Table 2 in which the authors examine the same classification tasks via a hypothesis testing framework with a null that can be rejected. PERMANOVA seems like a more straightforward way to test for associations between pairwise distances and groups, although it is possible that there are some subtleties of the data that we are missing. If PERMANOVA isn't appropriate, it seems possible that even a permutation test on average pairwise distance within versus between sets of genes that share a property against randomly reshuffled labels could help contextualize both the magnitude and significance of the separation."*

   We thank the reviewers for this excellent suggestion. In the revised manuscript, we have replaced the kNN classifier results with a more extensive set of results using PERMANOVA to test the associations between different groupings and the pairwise distance matrix.

3. *"Discussion of previous work could be described more clearly ('Comparison to previous work'). We really appreciated the efforts of the authors to contextualize their findings with respect to Kistler & Bedford, the two studies by Bhatt et al and Barrat-Charlaix & Neher, but we found this section somewhat challenging to follow logically. It might be useful to briefly report the findings from these studies (and this study) in some sort of comparative table showing what is similar and what differs between the different analyses? Part of the challenge is that the section jumps around chronologically and it was unclear to us in several places what studies or observations certain pronouns ('they' and 'their') referred to. Shorter paragraphs with more declarative topic sentences might help us follow the argument better. How do the authors explain the findings of Kistler & Bedford if these reference-specific reversions are neutral?"*

   We apologize that this section was not written clearly. In the revised manuscript, we rewrote this

entire section from scratch, focusing on reviewing previous work in chronological order, and in separate paragraphs. Additionally, we removed the statement about neutral evolution. Although the recent findings from Barrat-Charlaix and Neher are fascinating, we have not had time to explore the implications in depth, *i.e.*, in the context of Kistler and Bedford's work. Hence, this part has been omitted.

4. *"Did the distance metric used in MDS differ from Pond 2010? It might be useful to add a note that synchronizes language/terminology between the two papers."*

   The distance metric was the same. Unfortunately, several names have been used for this distance, including the Monge-Kantorovich distance (as used by Pond *et al.*), the Kantorovich-Rubinstein distance, the earth mover's distance, and the Wasserstein distance. We adopted the latter terminology to be consistent with the *transport* R package implementing this distance function, and because it is preferentially used in the literature. For example, there are currently about 42,800 hits for searches combining 'machine learning' with 'Wasserstein distance' and 'machine learning' in Google Scholar, about 3,170 hits with 'Kantorovich-Rubinstein', and about 2,900 with 'Monge-Kantorovich'. Similar results were obtained in combination with the keyword 'biology' instead of 'machine learning'. We have added a note to the Methods section clarifying that 'Monge-Kantorovich' and 'Wasserstein' refer to the same distance, with reference to the Pond *et al.* paper.

5. *"In Figures 1 and 3, it would be great to add visual captions that help explain what the different colors, symbols, fill statuses are beyond the written figure caption."*

   Thank you for this excellent suggestion. We have added colour and symbol keys to these figures, as well as similar figures in the Supplementary Materials.

6. *"One thing we wondered about was how phylogenetic correlations among the viruses you examine affect your output. For example, do you attribute the MDS clustering of enterovirus capsid proteins to their identity as capsid proteins, enterovirus proteins or the interaction between the two factors? Is there a way to get at this systematically? How widespread is EF clustering by viral family rather than protein category? While we feel this would be interesting/useful to analyze, it does not seem strictly critical if the authors consider it out of the scope of the work. If that is the case, we recommend discussing this point in the discussion."*

   Thank you for raising this point. To address this question, we used PERMANOVA to evaluate the association between virus families and the residualized Wasserstein distances. This analysis revealed that groups of evolutionary fingerprints corresponding to different virus families are significantly different. We also found that fingerprints from surface-exposed capsid proteins *within* the family Picornaviridae were significantly different from the other proteins from the same family. In other words, there is an interaction between these two factors: proteins from Picornaviridae (mostly enteroviruses) are different from other families; within Picornaviridae, the surface-exposed proteins are different from non-exposed proteins; and there was no significant difference between exposed and non-exposed proteins within other virus families. These new results have been incorporated into the revised manuscript.

Reviewer 3

1. *"The authors state they will 'focus on testing the hypothesis that surface-exposed proteins undergo more positive selection than other viral proteins', but evolutionary fingerprinting doesn't focus exclusively on positive selection, rather the entire selective regime. Further, an increase in mean dN/dS could be explained both an increase in diversifying selection and a decrease in purifying selection (as later noted by the authors in the Discussion). Overall, I think the framing of the question driving this study could benefit from increased focus."*

   We apologize for this poor choice of wording. The reviewer is correct that differences in both diversifying and purifying selection are important, and both are quantified by evolutionary fingerprints. Our original motivation for the study is that there are several examples of surface-exposed virus proteins with sites under significant diversifying selection. We wanted to know whether surface-exposed proteins tend to have different evolutionary fingerprints than other virus proteins. As it turns out, the answer was much more complicated than we expected — however, the results have also been quite fascinating. We have rewritten this paragraph to try to communicate our rationale more accurately.

2. *"Given the focus on surface proteins versus non-surface proteins, it is not clear why this analysis was not restricted to viruses that infect vertebrates with adaptive immune systems. The inclusion of plant viruses could bias the comparison of vertebrate virus surface and non-surface proteins. At a minimum, I would be interested in seeing a sensitivity analysis where all plant virus proteins were excluded."*

   As noted in our response to Reviewer 1, we have repeated our permutation test with the exclusion of all plant virus proteins.

3. *"What qualifies as a sufficient number of publicly available full-length genomes? How much divergence is sufficient for inclusion?"*

   As noted in the Methods section, we set a minimum requirement of 100 genome sequences as a starting criterion for data collection, as it was unlikely that viruses represented by substantially fewer sequences (*i.e.*, on the order of ten) would have sufficient genetic variation when many sequences are near identical. We subsequently required that the phylogeny relating sequences in a gene alignment had a minimum tree length (as a measure of divergence) of 0.5 expected substitutions per site. However, the results associated with these criteria have been replaced in the main text with updated results without reductions in tree lengths.

4. *"What criteria were used to define excessively long terminal branches to identify outlier sequences? Please quantify this statement."*

   We apologize for neglecting to provide this methodological detail. In the revised Methods section, we now note that outlier sequences are identified as having a branch length exceeding the mean length by two standard deviations for all terminal branches in the phylogeny.

5. *"Regarding the use of Wasserstein distance, it seems overly computationally burdensome for the output form FUBAR. The original evolutionary fingerprinting approach, co-authored by this manuscript's senior author, made use of the Wasserstein distance because the dN/dS values estimated in that paper were discrete. However, FUBAR provides a [more] continuous picture of the selection landscape, where the distance between the fingerprint between any two genes can determined as 1 minus the correlation between the dN/dS matrices."*

While the distance described by the reviewer is a valid method for comparing fingerprints, it has an important limitation in that each 'bin' representing a combination of dN and dS rates is treated as an independent observation without context. The fact that a bin is adjacent to another bin is not communicated through the correlation statistic. We have added the following text to the revised manuscript to explain the difference between these distance measures:

> An important advantage of the Wasserstein distance over alternatives such as the angular distance (related to the Pearson correlation coefficient) is that the latter penalizes differences in probability masses irrespective of where the masses are located. For example, consider three fingerprints (A, B and C) that respectively have 90% probability at grid points (1, 9), (1, 10) and (20, 20), and the remaining probability is otherwise uniformly distributed among points. The angular distance between A and B will be exactly the same as A and C, but the Wasserstein distance (W) recognizes that reshaping B to A requires much less work than C to A, resulting in $W_{AB} < W_{AC}$.

6. *"The phrase 'internal node that rooted a monophyletic clade' is multiply redundant. If there is not characteristic of this group, every internal node gives rise to a 'clade' of sorts. And a clade is, by definition, monophyletic."*

   We apologize for this misuse of terminology. In infectious disease research, the term 'clade' is used informally to refer to any contiguous subset of branches in the tree, *i.e.*, a subset tree. For example, the influenza A virus nomenclature system defines many clades that are not monophyletic. Nevertheless, in acknowledgment of this term's origin in the discipline of cladistics, we have replaced the phrase 'monophyletic clade' with 'monophyletic group'.

7. *"Figure 3. It would be helpful to have a key to indicate the meaning of color (rather than only supplying this information in the legend itself)."*

   A similar point was raised by Reviewer 2, and we have added a key as requested for this figure.

Sincerely,

Art F. Y. Poon, MSc PhD
Professor and Chair of Research-Based
Graduate Education

Department of Pathology and Laboratory Medicine
Schulich School of Medicine & Dentistry, Western University
Dental Sciences Building, Room 4144, London ON, Canada N6A 5C1
t: 519-661-2111 ext. 87978 e: apoon42@uwo.ca

Western
