# Supplementary material for: Selection profiles in RNA viruses reflect the characteristics of viruses more than individual proteins
Source: PLoS Pathog. 2026 Jul 24;22(7):e1014457. doi: 10.1371/journal.ppat.1014457 (PMC13432152; doi:10.1371/journal.ppat.1014457)
Supplement: Attachment — Submitted filename: response-letter-R2.pdf [file ppat.1014457.s017.pdf]

Pathology and
Laboratory Medicine

July 2, 2026

Dear colleagues,

Thank you for the opportunity to make further improvements to our manuscript based on the excellent feedback from Reviewer 1. We have reproduced each comment raised by the reviewer (along with a minor comment from Reviewer 2), along with our specific response, in the following itemized list.

Reviewer 1

1. *"To control for sequence and tree length, the authors ran a 2D MDS on their raw Wasserstein distances first, did a linear regression on those coordinates, and then calculated a new distance matrix 'dmx' from the residuals. While I appreciate the effort to control for these confounders, doing regression on low-dimensional coordinates like this is propbably not the best approach. By projecting everything to 2D before regressing, you throw away all the higher-dimensional variance where these confounders are still active. Tree length doesn't just affect the first two axes of variation; its effects are spread across the whole manifold. Additionally, this approach restricts the rank of the final distance matrix to at most 2, which ignores the actual high-dimensional shape of the Wasserstein distances.*
   A much simpler and standard way to do this is to just put the technical covariates directly into a sequential (Type I) PERMANOVA model of the original, unprojected Wasserstein distance matrix:
   *adonis2(wdist ∼ log(ncod) + log(treelen) + family + exposed * enveloped, data=mdat, by='terms')*
   This way, the model attributes variance to log(ncod) and log(treelen) first, controlling for them before testing the biological factors, all while using the full distance matrix."

   Thank you for pointing this out. The reviewer is absolutely correct that running PERMANOVA directly on the Wasserstein distance matrix makes more sense. Thus, we have incorporated the reviewer's recommended analysis into the revised manuscript. However, our previous approach of regressing out confounders and regenerating a new distance matrix enables us to visualize the results with the technical confounders largely suppressed. In contrast, it is more difficult to visualize differences between groups in the high dimensional space induced by the original Wasserstein distance matrix. Hence, we have replaced the statistical results from the previous PERMANOVA analysis with the reviewer's approach, but we needed to retain the previous regression method for generating some figures. This important distinction in handling the data is made explicit in the revised manuscript.

2. *"Right now, the authors use arbitrary integer grid indices (1 to 20) as coordinates for the Wasserstein distance calculation. A cleaner, more continuous approach is to use the actual*

FUBAR rate coordinates (alpha and beta) and apply a variance-stabilizing transformation like log(rate + 0.05).”

We thank the reviewer for providing an R script so that we were able to reproduce their demonstration using the continuous log-transformed coordinates. Following their recommendation, we have replaced the previous results with this approach, and we also added a note that qualitatively similar results were obtained using an integer coordinate system.

3. *“The authors found that Picornaviridae was the only family showing a significant difference between exposed and non-exposed proteins (R2 = 0.207, P = 0.0044). We should keep in mind that Picornaviridae is the largest family in the dataset (n = 48). The lack of significance in other families is likely just a lack of statistical power rather than a real biological difference, and this should be discussed.”*

   This is an excellent point, although we note that the largest family in the dataset is Flaviviridae, not Picornaviridae. We now acknowledge in association with this result that differences in sample size (*i.e.*, number of viruses per family) is an issue.

4. *“There are a few minor technical issues that make it hard to replicate the findings: The sequence alignments for Influenza B Virus (IBV) are missing from Zenodo, and the JSON grid files are missing from data/iss135/.”*

   We apologize for this. The IBV files were mistakenly omitted for the step3 data files, and have been restored in an update to the Zenodo repository. We have also uploaded the JSON files used in the previous revisions (iss135) to the same Zenodo repository as a tarball (`step4_json.tar.gz`).

5. *“The R script ‘iss135.R‘ fail[s] because reordering the metadata rows results in NA values for 36 proteins due to naming mismatches and for 5 proteins due to missing rows in stats files. When glm drops these, the 205 residuals mismatch the 246-element vectors in the plotting functions, which halts execution.”*

   We apologize for this inconvenience. We have updated the metadata CSV file and uploaded the JSON files required to reproduce the full analysis to the Zenodo repository, as noted above.

6. *“Researchers have to manually filter the dataset to the 205 valid proteins to run the script. The authors should clean up these naming mismatches and provide an executable script. To make this easy for the authors, I have attached a modified reproduction script ‘iss135_reproduce.R‘ which resolves all apparent name-matching errors and implements the log-rate coordinate toggle. Note that this analysis was conducted using the code and datasets on the git branch ‘iss135‘ (where the new revision data seems to be located, at least based on the metadata and modification history), as these files are not present on the ‘main‘ branch.”*

   Again, we apologize for this inconvenience. We now provide a script for reproducing the results in the study (`paper.R`) in the main branch of the GitHub repository. This script uses a network connection to pull the required datasets from the online GitHub and Zenodo repositories to make it easier for users to reproduce the analyses.

Reviewer 2

1. *"They should make sure to add a description of what color means in Figure 3 to the figure caption."*

   We apologize for this omission. A sentence has been added to describe the use of colour in the Figure in the caption as requested.

Sincerely,

Art F. Y. Poon, MSc PhD
Professor and Chair of Research-Based Graduate Education

Department of Pathology and Laboratory Medicine
Schulich School of Medicine & Dentistry, Western University
Dental Sciences Building, Room 4144, London ON, Canada N6A 5C1
t: 519-661-2111 ext. 87978 e: apoon42@uwo.ca

Western
