# Supplementary material for: Selection profiles in RNA viruses reflect the characteristics of viruses more than individual proteins
Source: PLoS Pathog. 2026 Jul 24;22(7):e1014457. doi: 10.1371/journal.ppat.1014457 (PMC13432152; doi:10.1371/journal.ppat.1014457)
Supplement: Peer review history

# Peer review: Decision Letter 0

**DOI:** 10.1371/journal.ppat.1014457.r001

**Authors:** Ronald Swanstrom, David Enard

4 Mar 2026

-->PPATHOGENS-D-26-00027

Evolutionary fingerprinting identifies divergent patterns of selection between enveloped and non-enveloped RNA viruses in surface-exposed proteins

PLOS Pathogens

Dear Dr. Poon,

Thank you for submitting your manuscript to PLOS Pathogens. After careful consideration, we feel that it has merit but does not fully meet PLOS Pathogens's publication criteria as it currently stands. Therefore, we invite you to submit a revised version of the manuscript that addresses the points raised during the review process.

Please submit your revised manuscript by May 03 2026 11:59PM. If you will need more time than this to complete your revisions, please reply to this message or contact the journal office at plospathogens@plos.org. When you're ready to submit your revision, log on to https://www.editorialmanager.com/ppathogens/ and select the 'Submissions Needing Revision' folder to locate your manuscript file.

Please include the following items when submitting your revised manuscript:

* A letter that responds to each point raised by the editor and reviewer(s). You should upload this letter as a separate file labeled 'Response to Reviewers'. This file does not need to include responses to any formatting updates and technical items listed in the 'Journal Requirements' section below.

* A marked-up copy of your manuscript that highlights changes made to the original version. You should upload this as a separate file labeled 'Revised Manuscript with Track Changes'.

* An unmarked version of your revised paper without tracked changes. You should upload this as a separate file labeled 'Manuscript'.

If you would like to make changes to your financial disclosure, competing interests statement, or data availability statement, please make these updates within the submission form at the time of resubmission. Guidelines for resubmitting your figure files are available below the reviewer comments at the end of this letter.

We look forward to receiving your revised manuscript.

Kind regards,

David Enard, PhD

Guest Editor

PLOS Pathogens

Ronald Swanstrom

Section Editor

PLOS Pathogens

Sumita Bhaduri-McIntosh

Editor-in-Chief

PLOS Pathogens

orcid.org/0000-0003-2946-9497

Michael Malim

Editor-in-Chief

PLOS Pathogens

orcid.org/0000-0002-7699-2064

**Additional Editor Comments :**

I thank the authors for submitting their manuscript to Plos Pathogens. Three reviewers have now shared their evaluations, and all three commended the manuscript. The reviewers particularly appreciated the importance of the question and the care taken to curate the data, including the care taken to work with good alignments. This is the basis of the kind of analysis conducted by the authors, yet it is too often neglected.

The reviewers (1 and 2 especially) make extensive recommendations to improve the statistical approaches used, and raise concerns that I share about a number of statistical limitations. First, reviewers 1 and 3 point out the confusion between negative and positive selection, and reviewer 1 suggests the solution of using the RELAX test of relaxation. This is important since differences in positive selection could be masked when they occurred on top of different backgrounds of negative selection.I agree with the reviewer that it would clarify the conclusions of the manuscript. Second, and I believe that this is an extremely important point raised by reviewer 1, the sub-sampling used could have indeed killed the statistical power to discern differences between classes of proteins. I agree with reviewer 1, and it is also my own experience that alignments of less than 100 codons typically result in very high variance, hence low discriminatory power. The authors should explore the suggestion to add the confounder of gene length as a covariate. Reviewers 1 and 2 make a number of other important recommendations about the statistical choices made. In their revision, the authors can implement these recommendations. If they succeed, the authors can modify their manuscript accordingly. If they fail, the authors can discuss that they tried them, and explain why they think they failed (limitations of the data, etc.). If the reviewers missed something that constitutively prevents the authors from being able to enact specific recommendations, the authors can add a detailed explanation to the discussion, or wherever appropriate in the revised manuscript. I look forward to receiving the revision.

**Journal Requirements:**

1) Please ensure that the CRediT author contributions listed for every co-author are completed accurately and in full.

At this stage, the following Authors/Authors require contributions: Art F. Y. Poon. Please ensure that the full contributions of each author are acknowledged in the "Add/Edit/Remove Authors" section of our submission form.

The list of CRediT author contributions may be found here: https://journals.plos.org/plospathogens/s/authorship#loc-author-contributions

2) We ask that a manuscript source file is provided at Revision. Please upload your manuscript file as a .doc, .docx, .rtf or .tex. If you are providing a .tex file, please upload it under the item type u2018LaTeX Source Fileu2019 and leave your .pdf version as the item type u2018Manuscriptu2019.

3) Please provide an Author Summary. This should appear in your manuscript between the Abstract (if applicable) and the Introduction, and should be 150-200 words long. The aim should be to make your findings accessible to a wide audience that includes both scientists and non-scientists. Sample summaries can be found on our website under Submission Guidelines:

https://journals.plos.org/plospathogens/s/submission-guidelines#loc-parts-of-a-submission

4) Please upload all main figures as separate Figure files in .tif or .eps format. For more information about how to convert and format your figure files please see our guidelines:

https://journals.plos.org/plospathogens/s/figures

5) We notice that your supplementary Figures, and Table are included in the manuscript file. Please remove them and upload them with the file type 'Supporting Information'. Please ensure that each Supporting Information file has a legend listed in the manuscript after the references list.

6) Please amend your detailed Financial Disclosure statement. This is published with the article. It must therefore be completed in full sentences and contain the exact wording you wish to be published.

1) State what role the funders took in the study. If the funders had no role in your study, please state: "The funders had no role in study design, data collection and analysis, decision to publish, or preparation of the manuscript.".

7) Please ensure that the funders and grant numbers match between the Financial Disclosure field and the Funding Information tab in your submission form. Note that the funders must be provided in the same order in both places as well.

Note: If the reviewer comments include a recommendation to cite specific previously published works, please review and evaluate these publications to determine whether they are relevant and should be cited. There is no requirement to cite these works unless the editor has indicated otherwise.

**Reviewers' Comments:**

Reviewer's Responses to Questions

**Part I - Summary**

Please use this section to discuss strengths/weaknesses of study, novelty/significance, general execution and scholarship.

Reviewer #1: In this manuscript, Muñoz-Baena et al. obtain and curate a large collection of enveloped and non-enveloped RNA viral genes, and perform an evolutionary fingerprinting analysis to associate selection pressure "signatures" with morphological, functional, and taxonomic features of the genes/viruses. The results are largely negative, claiming that the chosen "evolutionary descriptors" of gene evolution are unable to discriminate between proteins, including the surprising finding that surface-exposed and non-surface-exposed proteins could not be distinguished. The only signal reported by the authors is the ability to discriminate between surface proteins in enveloped and non-enveloped viruses, which the authors attribute to relaxed selection. This is a rather disappointing finding, which effectively states that from the standpoint of dN/dS-based metrics, viral proteins form an amorphous soup rather than following the commonly assumed differentiation between more conserved (e.g., polymerase/structural) and less conserved / more adaptively selected (surface, glyco) proteins.

The authors did a commendable job putting together an impressive collection of high-quality viral alignments and performing a sensible series of analyses. However, I have serious reservations about how the data were processed and analyzed. I believe that the lack of resolution reported in the paper is more likely the result of subsampling and other artifacts of the analytical process, rather than genuine biological reality. Below I explain my reservations and suggest why some of the decisions made by the authors are problematic. I recommend a complete re-analysis without the artificial subsampling and tree pruning. The statistical models must also be corrected for confounding and phylogenetic non-independence.

Reviewer #2: I reviewed this paper together with a graduate student with permission from the editor.

Muñoz-Baena et al examined how different viral properties (i.e., enveloped or not) and viral protein properties (i.e., surface-exposed or not) affected selection on viral proteins. To do so, they curated a collection of nearly 300 genes from 28 RNA viruses into a nice comparison set. To compare selective pressures, they relied on Pond 2010’s evolutionary fingerprinting (EF) method for inferring a posterior distribution over site-specific dn and ds values, and came up with novel ways of using the distance between these fingerprints to show similarities and differences among different groups. They also did some nice sensitivity analysis to diagnose challenges with the EF approach with respect to factors like alignment length and number of sequences. Using MDS, they found that surface-exposed and non-surface exposed EFs did not clearly differ, but that among surface-exposed proteins, enveloped and non-enveloped proteins clustered in seemingly distinct regions of the MDS space. They used a supervised learning approach, k nearest neighbors (KNN), to classify differences between EFs on a variety of tasks and discuss the results.

At a high level, we appreciated the curation of the data, the importance of the question, and the care that the authors took to validate potential challenges using the EF metric. The MDS plots were a useful vehicle to visualize these patterns at scale, and this approach is likely to be generalizable. The most substantial challenge we encountered was the general question of why the authors chose to use a machine learning KNN approach instead of a more standard statistical framework for hypothesis testing, and a section of the discussion contextualizing the results with respect to the literature.

Reviewer #3: Munoz-Baena and colleagues present a comprehensive investigation into the evolutionary fingerprints of surface and non-surface proteins of RNA viruses. This manuscript employs robust methodology, providing improved robustness for existing evolutionary fingerprinting techniques (i.e., accounting for datasets with varying degrees of genetic diversity). Although the authors do not find evidence for their initial hypothesis (surface exposed vs. non-exposed proteins), the authors report an intriguing difference between enveloped and non-enveloped viruses. Overall, I enjoyed the thoroughness of this investigation and its broadening of the discussion on selection on viral genes.

**********

**Part II – Major Issues: Key Experiments Required for Acceptance**

Please use this section to detail the key new experiments or modifications of existing experiments that should be absolutely required to validate study conclusions.

Generally, there should be no more than 3 such required experiments or major modifications for a "Major Revision" recommendation. If more than 3 experiments are necessary to validate the study conclusions, then you are encouraged to recommend "Reject".

Reviewer #1: 0. The authors make a specific, implicitly strong claim in the abstract: "we show that this pattern is more consistent with relaxed purifying selection than adaptive evolution in proteins associated with viral envelopes". However, the only arguments to this effect appeal to visual inspections of density plots and heatmaps, and are not supported by formal statistical tests. The claim in the abstract overstates the extent of analytical support for this attribution. Specific, formal tests for the relaxation of selection exist (e.g., the RELAX model in the HyPhy suite), or the authors must provide formal distributional tests on the dN/dS fingerprints to support this claim.

1. The authors (correctly) identified that dN/dS estimates have dataset-specific variance (heteroscedasticity). An estimate derived from a short and low-diversity alignment is highly uncertain, while an estimate from a long alignment derived over a larger tree is much more precise (in general). However, by randomly subsampling alignments down to 50 or 100 codons and aggressively pruning the terminal branches of phylogenetic trees, this approach equalized the variance by maximizing the noise across all datasets, effectively destroying the evolutionary signal that was the object of detection. I really struggle to understand the logic here. Averaging over high-variance estimates is not going to properly reduce the variance of the resulting estimate; it is going to reduce its resolution (make it "flatter"). Differential resolution is a feature that depends on the properties of the alignment, not a bug. If the concern is that clustering will reflect something about the length or the divergence of a gene, these covariates should be included directly in the statistical model rather than physically degrading the data.

A technical question regarding the Figure S1 simulation: If you simulate under INDELible (which uses a slightly different substitution model, e.g., GY94 with possibly different equilibrium frequencies) and infer with FUBAR, what exactly are you comparing via RMSE? FUBAR was not really designed to estimate true point values of dN and dS at a site super-accurately; it is meant to estimate the posterior probability of a site belonging to a specific rate class on a grid. A much more sensible approach would be to calculate the Wasserstein distance between the simulated distribution of dN/dS and the inferred FUBAR grid density.

2. Phylogenetic Confounding and Data Leakage.The supervised learning analysis (k-Nearest Neighbors) claims to distinguish surface proteins of enveloped versus non-enveloped viruses, but it is heavily confounded by viral taxonomy. The "non-enveloped" dataset is overwhelmingly dominated by a single viral family (Picornaviridae), with every other virus coming from its own family. The "enveloped" group is similarly dominated by Flaviviridae. By using standard Leave-One-Out Cross-Validation (LOOCV), the authors introduce significant possible data "leakage". When predicting the envelope status of a Dengue virus protein, the model can draw upon the relatively closely related Zika and West Nile proteins in the training set. The classifier could simply be capturing family-specific phylogenetic baseline traits rather than a generalizable "envelope" signature. A strict Leave-One-Family-Out cross-validation would be much more robust.

3. Calculating the Wasserstein distance using a linear Euclidean norm on a grid of dN/dS values misrepresents biological reality. The dN/dS scale is non-linear: the evolutionary "work" required to shift a site's rate from 0.0 (perfect conservation) to 0.1 (strong conservation) is vastly different from shifting it from 5.1 to 5.2 (very strong diversification to very strong diversification). A linear cost function likely overweights noisy variance in the high-rate tails.

4. The binomial regression (Figure 1B) is severely confounded by the statistical power of the selection inference method. The authors regress the log-odds of diversifying selection against the proportion of purifying sites. Both n+ and n- are counts of statistically significant sites, not true underlying biological states. Reaching significance is highly dependent on alignment-wide genetic variation (ie tree length). The observed negative correlation could very well be an artifact of the inference method losing statistical power (since negative selection is actually easier to detect for a given amount of sequence variation than positive selection).

5. The outliers discussed in the text (Rotavirus VP4/VP7 and Astrovirus VP27) are non-enveloped but were "misclassified" as Enveloped. Picornaviridae (which make up the bulk of the non-enveloped set) have rigid "canyon" capsids. Rotavirus and Astrovirus (the "misclassified" non-enveloped viruses) have protruding spikes, structurally similar to enveloped glycoproteins. This suggests a highly plausible biological explanation: the clustering is not driven by the presence of a lipid "Envelope," but rather by "Structural Protrusion / Spike Architecture."

6. The authors excluded plant viruses from the "surface-exposed" category because "plants do not have an adaptive immune system." This is an oversimplification. Plants have robust RNA interference (RNAi) and R-gene mediated immunity that exert strong diversifying selection on viral coat proteins and viral suppressors of RNA silencing, even though the targets and mechanisms are distinct from vertebrate humoral immunity. They should be included or treated as a distinct, formal control group.

Reviewer #2: 1. Poor performance of a machine learning classifier does not feel like strong evidence of a true non-separation between two groups. As we understood the role of KNN in this paper, this was the primary approach to quantify the differences qualitatively examined in MDS space under the assumption that high performing classifiers corresponded to true differences between groups and poor classifiers corresponded to no true difference between groups. However, there are many factors that can make a classifier perform poorly and comparing a series of ML performance attributes between different models did not feel like a straightforward way to assess the authors’ central question. For instance, the performance of leave one out cross validation with KNN framework is highly sensitive class imbalance (i.e., for comparing “Surface vs non-exposed” and “Polymerase vs. non-polymerase”, Table 2). While the authors briefly mentioned that the high accuracy and low F1 metric is likely due to class imbalance, it would be helpful to evaluate and interpret the performance with concrete numbers of class size for each task. More importantly, there is no null hypothesis that can be straightforwardly rejected (see below to major comment 2), so we are left guessing at what a poor classifier actually means. When is a classifier "good enough" that the authors would be convinced that there's true separation?

2. Relatedly, is there a reason that the authors did not use a formal hypothesis testing framework instead of fitting a ML model? It would be useful to report a version of Table 2 in which the authors examine the same classification tasks via a hypothesis testing framework with a null that can be rejected. PERMANOVA seems like a more straightforward way to test for associations between pairwise distances and groups, although it is possible that there are some subtleties of the data that we are missing. If PERMANOVA isn’t appropriate, it seems possible that even a permutation test on average pairwise distance within versus between sets of genes that share a property against randomly reshuffled labels could help contextualize both the magnitude and significance of the separation.

3. Discussion of previous work could be described more clearly (“Comparison to previous work”). We really appreciated the efforts of the authors to contextualize their findings with respect to Kistler & Bedford, the two studies by Bhatt et al and Barrat-Charlaix & Neher, but we found this section somewhat challenging to follow logically. It might be useful to briefly report the findings from these studies (and this study) in some sort of comparative table showing what is similar and what differs between the different analyses? Part of the challenge is that the section jumps around chronologically and it was unclear to us in several places what studies or observations certain pronouns (“they” and “their”) referred to. Shorter paragraphs with more declarative topic sentences might help us follow the argument better. How do the authors explain the findings of Kistler & Bedford if these reference-specific reversions are neutral?

Reviewer #3: The authors state they will “focus on testing the hypothesis that surface-exposed proteins undergo more positive selection than other viral proteins”, but evolutionary fingerprinting doesn’t focus exclusively on positive selection, rather the entire selective regime. Further, an increase in mean dN/dS could be explained both an increase in diversifying selection and a decrease in purifying selection (as later noted by the authors in the Discussion). Overall, I think the framing of the question driving this study could benefit from increased focus.

Given the focus on surface proteins versus non-surface proteins, it is not clear why this analysis was not restricted to viruses that infect vertebrates with adaptive immune systems. The inclusion of plant viruses could bias the comparison of vertebrate virus surface and non-surface proteins. At a minimum, I would be interested in seeing a sensitivity analysis where all plant virus proteins were excluded.

**********

**Part III – Minor Issues: Editorial and Data Presentation Modifications**

Please use this section for editorial suggestions as well as relatively minor modifications of existing data that would enhance clarity.

Reviewer #1: 1. The authors include a specific, implicitly strong claim "we show that this pattern is more consistent with relaxed purifying selection than adaptive evolution in proteins associated with viral envelopes". However the only arguments to this affect appeal to visual inspections of density plots, and are not supported by statistical claims. The claim in the abstract misstates the extent of analytical support for this attribution. Specific tests for relaxation of selection exist, for example, or even formal distributional tests on dN/dS fingerprint

2. The outliers you discuss (Rotavirus VP4/VP7 and Astrovirus VP27) are non-enveloped but "misclassified" as Enveloped. Picornaviridae (the bulk of your non-enveloped set) have rigid "canyon" capsids. Rotavirus and Astrovirus (the "misclassified" non-enveloped viruses) have protruding spikes, similar to Enveloped glycoproteins. This suggests a possible explanation for your clustering is not "Envelope" but "Structural Protrusion / Spike Architecture."

3. You excluded plant viruses from the "surface-exposed" category because "plants do not have an adaptive immune system." This is an oversimplification. Plants have robust RNA interference (RNAi) and R-gene mediated immunity that exert strong diversifying selection on viral coat proteins, even though the targets and mechanisms are distinct. They should be included or treated as a distinct control group.

Reviewer #2: 1. Did the distance metric used in MDS differ from Pond 2010? It might be useful to add a note that synchronizes language/terminology between the two papers.

2. In Figures 1 and 3, it would be great to add visual captions that help explain what the different colors, symbols, fill statuses are beyond the written figure caption.

3. One thing we wondered about was how phylogenetic correlations among the viruses you examine affect your output. For example, do you attribute the MDS clustering of enterovirus capsid proteins to their identity as capsid proteins, enterovirus proteins or the interaction between the two factors? Is there a way to get at this systematically? How widespread is EF clustering by viral family rather than protein category? While we feel this would be interesting/useful to analyze, it does not seem strictly critical if the authors consider it out of the scope of the work. If that is the case, we recommend discussing this point in the discussion.

Reviewer #3: Minor Comments

What qualifies as a sufficient number of publicly available full-length genomes? How much divergence is sufficient for inclusion?

What criteria were used to define excessively long terminal branches to identify outlier sequences? Please quantify this statement.

Regarding the use of Wasserstein distance, it seems overly computationally burdensome for the output form FUBAR. The original evolutionary fingerprinting approach, co-authored by this manuscript’s senior author, made use of the Wasserstein distance because the dN/dS values estimated in that paper were discrete. However, FUBAR provides a [more] continuous picture of the selection landscape, where the distance between the fingerprint between any two genes can determined as 1 minus the correlation between the dN/dS matrices.

The phrase “internal node that rooted a monophyletic clade” is multiply redundant. If there is not characteristic of this group, every internal node gives rise to a “clade” of sorts. And a clade is, by definition, monophyletic.

Figure 3. It would be helpful to have a key to indicate the meaning of color (rather than only supplying this information in the legend itself).

**********

PLOS authors have the option to publish the peer review history of their article (what does this mean?). If published, this will include your full peer review and any attached files.

If you choose “no”, your identity will remain anonymous but your review may still be made public.

**Do you want your identity to be public for this peer review?**  For information about this choice, including consent withdrawal, please see our Privacy Policy.

Reviewer #1: No

Reviewer #2: No

Reviewer #3: No

[NOTE: If reviewer comments were submitted as an attachment file, they will be attached to this email and accessible via the submission site. Please log into your account, locate the manuscript record, and check for the action link "View Attachments". If this link does not appear, there are no attachment files.]

**Figure resubmission:**

-->While revising your submission, we strongly recommend that you use PLOS’s NAAS tool (https://ngplosjournals.pagemajik.ai/artanalysis) to test your figure files. NAAS can convert your figure files to the TIFF file type and meet basic requirements (such as print size, resolution), or provide you with a report on issues that do not meet our requirements and that NAAS cannot fix.

After uploading your figures to PLOS’s NAAS tool - https://ngplosjournals.pagemajik.ai/artanalysis, NAAS will process the files provided and display the results in the "Uploaded Files" section of the page as the processing is complete. If the uploaded figures meet our requirements (or NAAS is able to fix the files to meet our requirements), the figure will be marked as "fixed" above. If NAAS is unable to fix the files, a red "failed" label will appear above. When NAAS has confirmed that the figure files meet our requirements, please download the file via the download option, and include these NAAS processed figure files when submitting your revised manuscript.

**Reproducibility:**

To enhance the reproducibility of your results, we recommend that authors of applicable studies deposit laboratory protocols in protocols.io, where a protocol can be assigned its own identifier (DOI) such that it can be cited independently in the future. Additionally, PLOS ONE offers an option to publish peer-reviewed clinical study protocols. Read more information on sharing protocols at https://plos.org/protocols?utm_medium=editorial-email&utm_source=authorletters&utm_campaign=protocols

# Author comment: Author response to Decision Letter 1

**DOI:** 10.1371/journal.ppat.1014457.r002

11 May 2026

Attachment Submitted filename: response-letter.pdf

# Peer review: Decision Letter 1

**DOI:** 10.1371/journal.ppat.1014457.r003

**Authors:** Ronald Swanstrom, David Enard

18 Jun 2026

PPATHOGENS-D-26-00027R1

Selection profiles in RNA viruses reflect the characteristics of viruses more than individual proteins

PLOS Pathogens

Dear Dr. Poon,

Thank you for submitting your manuscript to PLOS Pathogens. After careful consideration, we feel that it has merit but does not fully meet PLOS Pathogens's publication criteria as it currently stands. Therefore, we invite you to submit a revised version of the manuscript that addresses the points raised during the review process.

Please submit your revised manuscript by Aug 17 2026 11:59PM. If you will need more time than this to complete your revisions, please reply to this message or contact the journal office at plospathogens@plos.org. When you're ready to submit your revision, log on to https://www.editorialmanager.com/ppathogens/ and select the 'Submissions Needing Revision' folder to locate your manuscript file.

Please include the following items when submitting your revised manuscript:

* A letter that responds to each point raised by the editor and reviewer(s). You should upload this letter as a separate file labeled 'Response to Reviewers'. This file does not need to include responses to any formatting updates and technical items listed in the 'Journal Requirements' section below.

* A marked-up copy of your manuscript that highlights changes made to the original version. You should upload this as a separate file labeled 'Revised Manuscript with Track Changes'.

* An unmarked version of your revised paper without tracked changes. You should upload this as a separate file labeled 'Manuscript'.

If you would like to make changes to your financial disclosure, competing interests statement, or data availability statement, please make these updates within the submission form at the time of resubmission. Guidelines for resubmitting your figure files are available below the reviewer comments at the end of this letter.

As the corresponding author, your ORCID iD is verified in the submission system and will appear in the published article. PLOS supports the use of ORCID, and we encourage all coauthors to register for an ORCID iD and use it as well. Please encourage your coauthors to verify their ORCID iD within the submission system before final acceptance, as unverified ORCID iDs will not appear in the published article. Only the individual author can complete the verification step; PLOS staff cannot verify ORCID iDs on behalf of authors.

We look forward to receiving your revised manuscript.

Kind regards,

David Enard, PhD

Guest Editor

PLOS Pathogens

Ronald Swanstrom

Section Editor

PLOS Pathogens

Sumita Bhaduri-McIntosh

Editor-in-Chief

PLOS Pathogens

orcid.org/0000-0003-2946-9497

Michael Malim

Editor-in-Chief

PLOS Pathogens

orcid.org/0000-0002-7699-2064

**Additional Editor Comments (if provided):**

All reviewers strongly praised the authors for their extensive effort revising their manuscript. One reviewer is still requiring a few more improvements related to the new methods introduced. Before I can make a final decision, I encourage the authors to implement the suggested improvements so that the manuscript becomes even stronger. I am aware that asking the authors to go through multiple revisions can be a pain, but in that case one reviewer has made additional suggestions that I do think will improve the manuscript to make it very strong. I look forward to receiving the revision.

**Reviewers' Comments:**

Reviewer's Responses to Questions

**Part I - Summary**

Please use this section to discuss strengths/weaknesses of study, novelty/significance, general execution and scholarship.

Reviewer #1: Overall, the revised manuscript by Munoz-Baena et al. is a really solid step forward compared to their first submission. The authors did a great job responding to the initial round of feedback, particularly by swapping out their original machine learning framework for a proper hypothesis-testing PERMANOVA model. They also cleaned up some of their unsupported selection relaxation claims and added helpful discussions on taxonomic confounding and plant immunity. Plus, their commitment to open science—making the data available on Zenodo and sharing their code on GitHub—is fantastic and sets a high bar for the field. Most of my previous concerns are addressed here. That said, the new 2D residualization method they came up with to control for sequence and tree length is a bit unorthodox, and there are some minor data-matching bugs in their R script that make the analysis fail when run from scratch.

Reviewer #2: I thank the authors for their thoughtful and thorough re-analysis and re-writing, and have no major outstanding concerns about the manuscript as submitted.

Reviewer #3: I have no further suggestions or concerns

**********

**Part II – Major Issues: Key Experiments Required for Acceptance**

Please use this section to detail the key new experiments or modifications of existing experiments that should be absolutely required to validate study conclusions.

Generally, there should be no more than 3 such required experiments or major modifications for a "Major Revision" recommendation. If more than 3 experiments are necessary to validate the study conclusions, then you are encouraged to recommend "Reject".

Reviewer #1: 1. Methodological Critique of the New Residualization Method

To control for sequence and tree length, the authors ran a 2D MDS on their raw Wasserstein distances first, did a linear regression on those coordinates, and then calculated a new distance matrix `dmx` from the residuals. While I appreciate the effort to control for these confounders, doing regression on low-dimensional coordinates like this is propbably not the best approach. By projecting everything to 2D before regressing, you throw away all the higher-dimensional variance where these confounders are still active. Tree length doesn't just affect the first two axes of variation; its effects are spread across the whole manifold. Additionally, this approach restricts the rank of the final distance matrix to at most 2, which ignores the actual high-dimensional shape of the Wasserstein distances.

A much simpler and standard way to do this is to just put the technical covariates directly into a sequential (Type I) PERMANOVA model of the original, unprojected Wasserstein distance matrix:

adonis2(wdist ~ log(ncod) + log(treelen) + family + exposed * enveloped, data=mdat, by='terms')

This way, the model attributes variance to log(ncod) and log(treelen) first, controlling for them before testing the biological factors, all while using the full distance matrix.

When I run this sequential model on the 205 valid proteins, log(ncod) explains 58.3% of the variance, log(treelen) explains 2.8%, and family explains 12.9%. The biological factors explain a very small slice: exposure explains 0.36% (marginally significant, P = 0.082) and the interaction term explains 0.56% (statistically significant, P = 0.025).

As it turns out, the authors' 2D residualization method inflates the apparent effect size of the biological interaction by about 2.5-fold. In their 2D residualized space, the interaction term explains 3.7% of the variance, but in the full space, it only explains 1.44% of the remaining variance after controlling for covariates. This happens because the pre-regression MDS projection discards 71.7% of the total variance, which artificially shrinks the denominator. Running the sequential model on the full distance matrix gives a much more transparent and mathematically rigorous look at the actual effect size.

2. Log-Transformed Actual Rates vs. Integer Grid Indices

Right now, the authors use arbitrary integer grid indices (1 to 20) as coordinates for the Wasserstein distance calculation. A cleaner, more continuous approach is to use the actual FUBAR rate coordinates (alpha and beta) and apply a variance-stabilizing transformation like log(rate + 0.05). I re-ran the entire analysis using log-rate coordinates, and here is what happens:

- The new distance matrix correlates very strongly with the index-based one (r = 0.966), showing the overall geometry is preserved.

- However, in the sequential PERMANOVA, using the continuous log-rate coordinates actually reduces the variance explained by the technical confounder log(ncod) from 58.3% to 51.4%, while increasing the biological interaction term from 0.56% to 0.86% (and making it more significant: P = 0.0089 vs P = 0.025).

- In their 2D residualized space, the interaction term explains 5.5% of the variance (compared to 3.7% using indices) with a highly significant P-value of 0.0002.

This tells us that the grid discretization indices introduce artifacts that actually compound the confounding and weaken the biological signal. The authors should consider switching to continuous log-transformed rate coordinates.

Reviewer #2: (No Response)

Reviewer #3: (No Response)

**********

**Part III – Minor Issues: Editorial and Data Presentation Modifications**

Please use this section for editorial suggestions as well as relatively minor modifications of existing data that would enhance clarity.

Reviewer #1: 1. Family-Wise Power:

The authors found that Picornaviridae was the only family showing a significant difference between exposed and non-exposed proteins (R2 = 0.207, P = 0.0044). We should keep in mind that Picornaviridae is the largest family in the dataset (n = 48). The lack of significance in other families is likely just a lack of statistical power rather than a real biological difference, and this should be discussed.

2. Reproducibility Issues:

There are a few minor technical issues that make it hard to replicate the findings:

- The sequence alignments for Influenza B Virus (IBV) are missing from Zenodo, and the JSON grid files are missing from data/iss135/.

- The R script `iss135.R` fail because reordering the metadata rows results in NA values for 36 proteins due to naming mismatches and for 5 proteins due to missing rows in stats files. When glm drops these, the 205 residuals mismatch the 246-element vectors in the plotting functions, which halts execution.

- Researchers have to manually filter the dataset to the 205 valid proteins to run the script. The authors should clean up these naming mismatches and provide an executable script.

- - To make this easy for the authors, I have attached a modified reproduction script `iss135_reproduce.R` which resolves all apparent name-matching errors and implements the log-rate coordinate toggle. Note that this analysis was conducted using the code and datasets on the git branch `iss135` (where the new revision data seems to be located, at least based on the metadata and modification history), as these files are not present on the `main` branch.

Reviewer #2: They should make sure to add a description of what color means in Figure 3 to the figure caption.

Reviewer #3: (No Response)

**********

PLOS authors have the option to publish the peer review history of their article (what does this mean?). If published, this will include your full peer review and any attached files.

If you choose “no”, your identity will remain anonymous but your review may still be made public.

**Do you want your identity to be public for this peer review?**  For information about this choice, including consent withdrawal, please see our Privacy Policy.

Reviewer #1: No

Reviewer #2: No

Reviewer #3: No

[NOTE: If reviewer comments were submitted as an attachment file, they will be attached to this email and accessible via the submission site. Please log into your account, locate the manuscript record, and check for the action link "View Attachments". If this link does not appear, there are no attachment files.]

**Figure resubmission:**

-->While revising your submission, we strongly recommend that you use PLOS’s NAAS tool (https://ngplosjournals.pagemajik.ai/artanalysis) to test your figure files. NAAS can convert your figure files to the TIFF file type and meet basic requirements (such as print size, resolution), or provide you with a report on issues that do not meet our requirements and that NAAS cannot fix.

After uploading your figures to PLOS’s NAAS tool - https://ngplosjournals.pagemajik.ai/artanalysis, NAAS will process the files provided and display the results in the "Uploaded Files" section of the page as the processing is complete. If the uploaded figures meet our requirements (or NAAS is able to fix the files to meet our requirements), the figure will be marked as "fixed" above. If NAAS is unable to fix the files, a red "failed" label will appear above. When NAAS has confirmed that the figure files meet our requirements, please download the file via the download option, and include these NAAS processed figure files when submitting your revised manuscript.

**Reproducibility:**

To enhance the reproducibility of your results, we recommend that authors of applicable studies deposit laboratory protocols in protocols.io, where a protocol can be assigned its own identifier (DOI) such that it can be cited independently in the future. Additionally, PLOS ONE offers an option to publish peer-reviewed clinical study protocols. Read more information on sharing protocols at https://plos.org/protocols?utm_medium=editorial-email&utm_source=authorletters&utm_campaign=protocols

Attachment Submitted filename: iss135_reproduce.R

# Author comment: Author response to Decision Letter 2

**DOI:** 10.1371/journal.ppat.1014457.r004

7 Jul 2026

Attachment Submitted filename: response-letter-R2.pdf

# Editor report: Decision Letter 2

**DOI:** 10.1371/journal.ppat.1014457.r005

**Authors:** Ronald Swanstrom, David Enard

9 Jul 2026

Dear Dr. Poon,

We are pleased to inform you that your manuscript 'Selection profiles in RNA viruses reflect the characteristics of viruses more than individual proteins' has been provisionally accepted for publication in PLOS Pathogens.

Before your manuscript can be formally accepted you will need to complete some formatting changes, which you will receive in a follow up email. A member of our team will be in touch with a set of requests.

Please note that your manuscript will not be scheduled for publication until you have made the required changes, so a swift response is appreciated.

IMPORTANT: The editorial review process is now complete. PLOS will only permit corrections to spelling, formatting or significant scientific errors from this point onwards. Requests for major changes, or any which affect the scientific understanding of your work, will cause delays to the publication date of your manuscript.

Should you, your institution's press office or the journal office choose to press release your paper, you will automatically be opted out of early publication. We ask that you notify us now if you or your institution is planning to press release the article. All press must be co-ordinated with PLOS.

Thank you again for supporting Open Access publishing; we are looking forward to publishing your work in PLOS Pathogens.

Best regards,

David Enard, PhD

Guest Editor

PLOS Pathogens

Ronald Swanstrom

Section Editor

PLOS Pathogens

Sumita Bhaduri-McIntosh

Editor-in-Chief

PLOS Pathogens

orcid.org/0000-0003-2946-9497

Michael Malim

Editor-in-Chief

PLOS Pathogens

orcid.org/0000-0002-7699-2064

***********************************************************

The authors have now thoroughly addressed all reviewer's comments. The manuscript is now very strong and the authors should be praised for this very interesting work. It is a very valuable contribution.

Reviewer Comments (if any, and for reference):

# Editor report: Acceptance letter

**DOI:** 10.1371/journal.ppat.1014457.r006

**Authors:** Ronald Swanstrom, David Enard

Dear Dr. Poon,

We are delighted to inform you that your manuscript, "Selection profiles in RNA viruses reflect the characteristics of viruses more than individual proteins," has been formally accepted for publication in PLOS Pathogens.

We have now passed your article onto the PLOS Production Department who will complete the rest of the pre-publication process. All authors will receive a confirmation email upon publication.

The corresponding author will soon be receiving a typeset proof for review, to ensure errors have not been introduced during production. Please review the PDF proof of your manuscript carefully, as this is the last chance to correct any scientific or type-setting errors. Please note that major changes, or those which affect the scientific understanding of the work, will likely cause delays to the publication date of your manuscript. Note: Proofs for Front Matter articles (Pearls, Reviews, Opinions, etc...) are generated on a different schedule and may not be made available as quickly.

Soon after your final files are uploaded, the early version of your manuscript, if you opted to have an early version of your article, will be published online. The date of the early version will be your article's publication date. The final article will be published to the same URL, and all versions of the paper will be accessible to readers.

For Research Articles, you will receive an invoice from PLOS for your publication fee after your manuscript has reached the completed accept phase. If you receive an email requesting payment before acceptance or for any other service, this may be a phishing scheme. Learn how to identify phishing emails and protect your accounts at https://explore.plos.org/phishing.

Thank you again for supporting open-access publishing; we are looking forward to publishing your work in PLOS Pathogens.

Best regards,

Sumita Bhaduri-McIntosh

Editor-in-Chief

PLOS Pathogens

orcid.org/0000-0003-2946-9497

Michael Malim

Editor-in-Chief

PLOS Pathogens

orcid.org/0000-0002-7699-2064
